# Disinfection of SARS-CoV-2 by UV-LED 267 nm: comparing different variants

**DOI:** 10.1038/s41598-023-35247-9

**Published:** 2023-05-22

**Authors:** Nofar Atari, Hadas Mamane, Alon Silberbush, Neta Zuckerman, Michal Mandelboim, Yoram Gerchman

**Affiliations:** 1 Central Virology Laboratory, Ministry of Health, Chaim Sheba Medical Center, Tel-Hashomer, Ramat-Gan, Israel; 2 School of Mechanical Engineering, Faculty of Engineering, Tel Aviv University, 69978 Tel Aviv, Israel; 3 Department of Biology and Environment, Faculty of Natural Sciences, University of Haifa-Oranim, Kiryat Tiv’on, Israel; 4 Department of Epidemiology and Preventive Medicine, School of Public Health, Tel-Aviv University, Tel Aviv, Israel; 5 The Institute of Evolution, University of Haifa, Haifa, Israel; 6 Oranim College, 3600600 Tivon, Israel

**Keywords:** SARS-CoV-2, Viral infection

## Abstract

UV irradiation is an efficient tool for the disinfection of viruses in general and coronavirus specifically. This study explores the disinfection kinetics of SARS-CoV-2 variants *wild type* (similar to the Wuhan strain) and three variants (*Alpha, Delta, and Omicron*) by 267 nm UV-LED. All variants showed more than 5 logs average reduction in copy number at 5 mJ/cm^2^ but inconsistency was evident, especially for the Alpha variant. Increasing the dose to 7 mJ/cm^2^ did not increase average inactivation but did result in a dramatic decrease in the inactivation inconsistency making this dose the recommended minimum. Sequence analysis suggests that the difference between the variants is likely due to small differences in the frequency of specific UV extra-sensitive nucleotide sequence motifs although this hypothesis requires further experimental testing. In summary, the use of UV-LED with their simple electricity need (can be operated from a battery or photovoltaic panel) and geometrical flexibility could offer many advantages in the prevention of SARS-CoV-2 spread, but minimal UV dose should be carefully considered.

## Introduction

The efficiency of ultraviolet (UV) irradiation depends on multiple factors such as UV dose, irradiance, irradiation source, microorganism and strain type, matrix, and UV wavelength. UV light-emitting diodes (UV LEDs) emit UV light at specific wavelengths with relatively narrow full width at half maximum (FWHM) bandwidths. For example, UV LED or polychromatic mercury wavelengths in the germicidal range showed lower effectiveness at higher UV wavelengths^1,2^, while discrepancies of the time–dose reciprocity law were found for UV LED of different wavelengths and UV-damage mechanisms (Ref.^3^; also see Table 2).

UV irradiation is efficient in the inactivation of viruses in various environments such as aqueous solutions^1,4^, on surfaces^5,6^, and in air/bioaerosols^7,8^. UV irradiation was also found efficient in the inactivation of human coronaviruses (e.g., hOC43^1^) and severe acute respiratory syndrome coronavirus 2 (SARS-CoV2)^9,10^. However, the SARS-CoV-2 outbreaks are characterized by the rapid appearance of variants^11,12^, making comparisons between studies done on different SARS-CoV-2 variants complicated. Our previous study examined the impact of UV LED wavelengths on one strain of human coronavirus (hCV-43^1^). Here we examined the sensitivity of four different variants of the SARS-CoV-2 (*wild type*, *Alpha, Delta*, and *Omicron*) to UV-LED germicidal wavelength with peak emission at 267 nm.

## Results and discussion

**Figure 1:**
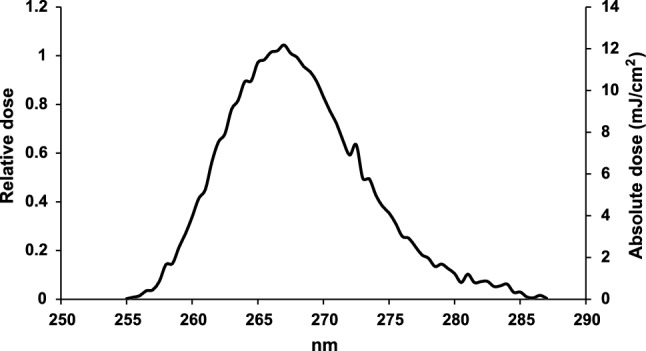
Emission spectra of the 267 nm UV LED used in this study.

The UV-LED spectra exhibited peak emission at 267 nm with narrow FWHM bandwidth of 12 nm (Fig. 1).

**Figure 2:**
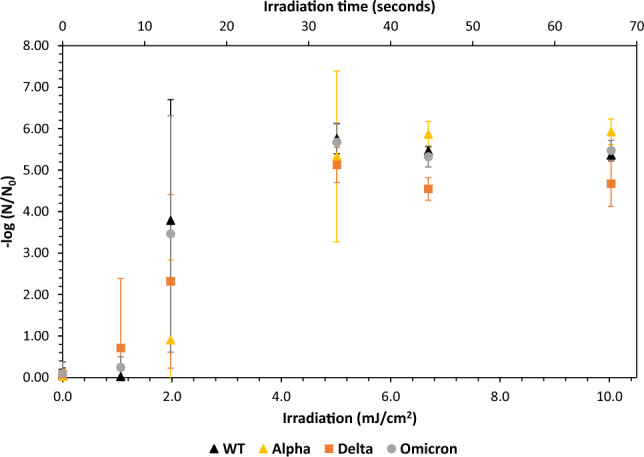
UV267nm irradiation time and dose–response curves of the different SARS-CoV-2 variants (weighted incident irradiance 0.152 mW/cm^2^). Data points are averages (N = 11–16 for 0 to 5 mJ/cm^2^ and N = 4 for 7 mJ/cm^2^ and above). Error bars denote 1 SD.

At dose zero (no UV exposure), a variation was found among initial virus copy numbers of the different variants but one-way ANOVA analysis revealed no statistically significant difference (F_3,60_ = 0.2941, p = 0.83). The irradiation time and dose (fluence) response curve of different SARS-CoV-2 variants to 267 nm UV LED is presented in Fig. 2. First statistically significant inactivation for the *w.t., Delta and Omicron* variants required exposure to 2 mJ/cm^2^, while the Alpha variant required 5 mJ/cm^2^ (Fig. 2b), with higher doses resulting in plateauing up to the maximal dose (10 mJ/cm^2^). Interestingly, variability in the inactivation efficiency was both UV dose- and variant-dependent, as evident by the error bars size (Fig. 2) and the large coefficient of variance, especially for the *Alpha* variant (Table 1). This variability at the lower doses should play a role when designing a disinfection system to mitigate coronaviruses and a UV-LED dose of 7 mJ/cm^2^ is recommended. Additionally, the LED incident irradiance was low in this case—however more powerful LEDs (leading to higher radiant flux at a given exposure time) could mitigate the risk in this variability and should be examined.

**Table 1:** Coefficient of Variation for different variant-UV dose combinations.

Variant		− Log (N/N_0_) coefficient of variation (absolute numbers)				
	UV dose (mg/cm^2^)					
	0 (N = 16)	1 (N = 13–16)	2 (N = 11–15)	5 (N = 14–16)	7 (N = 4)	10 (N = 4)
w.t	500%	1350%	77%^a^	6%	2%	3%
Alpha	515%	1984%	213%	39%^b^	5%	5%
Delta	368%	234%	90%^c^	8%	6%	12%
Omicron	320%	106%	82%^d^	8%	5%	4%

Superscript alphabets donate first significant inactivation as determined by Tukey HSD Post-hoc Test.

^a^*w.t.* one-way ANOVA of irradiation dose F(5,61) = 46.22, p < 0.0001; Tukey HSD Post-hoc Test p < 0.0001.

^b^*Alpha* one-way ANOVA of irradiation dose F(5,63) = 47.0, p < 0.0001; Tukey HSD Post-hoc Test p < 0.0001.

^c^*Delta* one-way ANOVA of irradiation dose F(5,62) = 32.9, p < 0.0001; Tukey HSD Post-hoc Test p = 0.0002.

^d^*Omicron* one-way ANOVA of irradiation dose F(5,62) = 44.11, p < 0.0001; Tukey HSD Post-hoc Test p < 0.0001.

To decipher the mechanism underlying the different inactivation efficiency we looked further into the variant's sequences focusing on the sequences stretch YTTC and YCTY ('Y' being C or T), consensus for the highest intensity UV-induced 6-4PP adduct and CPD damage respectively^17^. Table 2 report the number of appearances of these sequences in the different variant's sequences (see sequence alignment in Supplementary Fig. S1), demonstrating the *Alpha* variant has the lowest appearance of such sequences, in agreement with its higher tolerance and variance in response to UV at intermediate doses (i.e. 2 mJ/cm^2^). This hypothesis, if correct, would suggest small changes in the virus genome sequence could result in dramatic changes in its UV resistance, and should be considered when looking for the use of UV irradiation as way to combat pathogenic viruses.

**Table 2:** Appearance of highly UV sensitive sequences in the variant's genomes.

Variant		Sequence						
		YTTC		YCTY				Sum of appearances
		CTTC	TTTC	CCTC	CCTT	TCTC	TCTT	
*w.t*	EPI_ISL_745046	125	157	55	119	71	203	730
*Alpha*	EPI_ISL_737204	121	155	54	116	72	203	721
Delta	EPI_ISL_2183060	123	156	53	119	71	204	726
Omicron	EPI_ISL_7869197	123	157	51	117	74	202	724

The data presented in Fig. 2b fit nicely with the previously published results, both in suggesting UVC can be effective against SARS-CoV-2 viruses and doses needed for efficient inactivation (Table 3). It is noteworthy that previous published data on the inactivation of SARS-CoV-2 virus in suspension suggested higher doses needed for 3-log reduction (Compare Table 3, lines 1, 4, 6, 7 and 8), probably due to the addition of protein to the suspension^8^, serving as UV absorbent. Where suspension and aerosols were directly compared^8^ much lower doses were required for similar activation of the virus in the latter (see line 7 in Table 3), probably due to the much smaller droplet size in the aerosol (in Ref.^8^ more than 80% of the droplets were smaller than 1 µm while the suspension droplets were probably ~ 1 mm, given they used similar volume to that presented here).

**Table 3:** UV dose required for 3-log inactivation of SARS-CoV-2 variants.

#	Virus/variant	UV source	Conditions	Dose needed for a 3-log reduction	References
1	SARS-CoV-2: w.t. (Wuhan strain), alpha (B.1.1.7 501Y.V1), delta (B.1.617.2), omicron (B.1.1.529)	UV-LED 267 nm	Suspension	2–5 mJ/cm^2^	This study
2	hCoV-OC43	UV-LED (267, 275, 285. 295 nm)	Suspension	5.6–32 mJ/cm^2^, wavelength-dependent	^1^
3	SARS-CoV-2 Isolate USA-WA1/2020	LP-UV (254 nm)	Aerosol	Not available	^7^
4	SARS-CoV-2 clade 20A (lineage B.1)	LP-UV (254 nm)	Suspension	14.5 for polystyrene surface and 9.8 mJ/cm^2^ for glass and stainless steel	^6^
5	SARS-CoV-2 USA-WA1/2020	Pulsed xenon ultraviolet (200–320 nm)	Suspension dried on surface and on N95 masks	Not available	^5^
6	SARS-CoV-2 SB3-TYAGNC, HCoV-229E, HCoV-OC43, Others	LP-UV (254 nm)	Suspension	7.5 mJ/cm^2^	^4^
7	SARS-CoV-2 UT-NCGM02/Human/2020/Tokyo	UV-LED 265 nm	Suspension and Aerosol. 1% protein (bovine serum albumin) added	8.3 and 1 mJ/cm^2^ for suspension and aerosol respectively	^8^
8	SARS-CoV-2 (isolate USA WA1 2020)	KrCl (222 nm), LP-UV (254 nm), UV-LEDs (270 and 282 nm)	Suspension	2.5, 2.2, 3.3, and 6 mJ/cm^2^, with the different irradiation sources	^2^

In summary, both our and previous results suggest that the UVC irradiation can be used to combat human coronavirus while mitigating the environmental effects of using disinfectants and allow reuse of respiration masks^9,18^ lowering plastics wastes originating from such^19^. Moreover, the use UV as a disinfectant can reduce the use of environmentally problematic chemical disinfecting agent^20^ and mercury containing UV lamps (in line with the Minamata convention to reduce global mercury pollution). UV-LEDs small format and simple electrical circuitry needed for UVC LEDs could also support their incorporation into air ventilation systems^21^ although such application is still limited by the UV-LED emission efficiency.

## Materials and methods

Four SARS-CoV-2 variants were used in this study: *w.t.* (w.t-like strain, B.1.1.50, that circulated in Israel 2020); *Alpha* (B.1.1.7 501Y.V1), containing multiple spike mutations, demonstrated to have 70% higher transmission rate than the *w.t.* strain^13^; *Delta* (B.1.617.2), reported more infectious and causing more severe disease compared to the *Alpha* variant^13^; and *Omicron* (B.1.1.529), containing more than thirty amino acid mutations in the spike protein, and demonstrating mutation rate exceeding that of other variants by 5–11 times as well as enhanced transmissibility and immune evasion^14^. All the virus variants were isolated at the Mandelbaum lab from leftover respiratory swabs samples (fully anonymized samples) used for routine diagnosis and found positive for SARS-CoV-2. All protocols were conducted under Sheba Medical Center Helsinki Committee approval (number 7875-20-SMC), and under these circumstances’ hospitals don’t require informed consent. Viruses were identified by sequencing (sequences deposited in the NCBI genebank database under accession numbers OQ948263 to OQ948266, respectively. Sequences can also be found in the https://gisaid.org/ under accession numbers EPI_ISL_745046, EPI_ISL_737204, EPI_ISL_2183060 and EPI_ISL_7869197, respectively). Propagation of the viruses was as previously described^15^.

UV source was a custom-made UV-LEDs device built in collaboration with AquiSense, having a peak emission wavelength at 267 nm (Fig. 1)^1,16^. Weighted incident irradiance was 0.152 mW/cm^2^ at the center of the exposure area (measured with a calibrated Ocean Optics USB4000 spectroradiometer equipped with a cosine corrector and integrated for 250–290 nm). UV dose (mJ/cm^2^) was determined by multiplying the measured irradiation (mW/cm^2^) by irradiation time (seconds).

Virus irradiation was done as previously described^1^. Briefly, virus suspension was diluted in Eagle's Minimum Essential Medium without phenol red (UVT > 95%) to a concentration of 10 × 100TCID50 (i.e. 1000-fold the dilution of a virus required to infect 50% of the cells in the cell culture^22^, here within 5 days of infection). Fifty µl of this virus suspension was placed in each well of a black 24-well plate (giving a layer of ~ 1 mm height in the highest point). All wells were covered with black insulation tape. Each time before irradiation, the tape was removed from a 4-well column or 6-well row for the designated time^1,16^, resulting in 4 or 6 replicates per plate, accordingly. In each plate tested a column/row of wells was left covered throughout the irradiation to serve as noUV control and as 0 irradiance reference. The process was repeated three times for the shorter irradiation times (1, 2, and 5 mJ/cm^2^) due to significant variability in results. Zero-irradiation control was kept covered with the tape throughout the irradiation process to allow for other effects.

Virus quantification was done after proliferation, thus quantifying only infective capable viruses. To this end, after irradiation 450 µl Eagle's Minimum Essential Medium supplemented with 2% (v/v) fetal calf serum (MEM- EAGLE) was added to each well (including the “no UV” wells); the content was mixed by pipettation, and 50 µl were transferred to a well of 96-well plate (Applied Biosystems, USA) containing 24-h-old (80–90% confluency) Vero-E6 cells in the same medium, giving final virus concentration of 100TCID50 per well (for pre-irradiated viruses). The plates were incubated for 1 h at 33 °C, unattached viruses washed with media by pippeting, and 200 μL MEM-EAGLE medium containing 2% FCS was added. The cells were then incubated in a humidified incubator with 5% CO_2_ at 33 °C for five additional days. Total RNA was extracted from the cells using a MagNA Pure 96 Instrument (Roche Life Science) according to the manufacturer’s protocol. Virus copy number in the cells was determined by reverse transcriptase − qPCR (done in CFX-96 thermocycler, Bio-Rad, USA) and compared against a calibration curve constructed from virus solutions of known titers. Oligonucleotides used were E_Sarbeco_F (ACAGGTACGTTAATAGTTAATAGCGT) and E_Sarbeco_R (ATATTGCAGCAGTACGCACACA) and the probe was E_Sarbeco_P1(FAM-ACACTAGCCATCCTTACTGCGCTTCG-BBQ), with conditions as described in Ref.^23^.

### Inactivation and statistical analysis

Log inactivation was calculated for each variant-preparation combination separately as log (N_0_/N), N and N_0_ being viral concentrations with and without irradiation respectively, to correct for variability in initial virus numbers (both for different preparation of the same variant and between variants on the same preparation date). To this end, the virus number in each well (Eq. 1, N_dose-variant_) was divided by average number of viruses of the corresponding average specific variants noUV wells in the same plate (Eq. 1 N_0-variant_).

1 $$-\mathrm{log}\left(\frac{{N}_{dose-varient}}{{N}_{0-varient}}\right).$$

All analyses were done with SPSS statistics for windows v.24 (IBM, Released 2016) with type III sums of squares.

### Sequence analysis

Quantification of UV sensitive motifs was done in R (version 4.1.3; 2022-03-10) using custom code (see supplementary information). Sequence comparison was done using the MAFFT algorithm (https://www.ebi.ac.uk/Tools/msa/mafft/) and alignment using Clustal Omega (https://www.ebi.ac.uk/Tools/msa/clustalo/).

This research was partially funded by Oranim Academic College internal grant and by the Tel Aviv University Center for Combatting Pandemics (TCCP).

## Supplementary Information

Supplementary Information.

## Data Availability

Sequences of the variants used in this study were deposited in (sequences deposited in the NCBI genebank database under accession numbers OQ948263 (*w.t.*), OQ948264 (*Alpha*), OQ948265 (*Delta*), OQ948266 (*Omicron*). Sequences can also be found in the https://gisaid.org/ under accession numbers EPI_ISL_745046, EPI_ISL_737204, EPI_ISL_2183060 and EPI_ISL_7869197, respectively) https://gisaid.org/ under accession numbers EPI_ISL_745046 (*w.t.*), EPI_ISL_737204 (*Alpha*), EPI_ISL_2183060 (*Delta*) and EPI_ISL_7869197 (*Omicron*)*.*
