# Supplementary material for: Disinfection of SARS-CoV-2 by UV-LED 267 nm: comparing different variants
Source: Sci Rep. 2023 May 22;13:8229. doi: 10.1038/s41598-023-35247-9 (PMC10201513; doi:10.1038/s41598-023-35247-9)
Supplement: Supplementary file 1 — Supplementary Information. [file 41598_2023_35247_MOESM1_ESM.pdf]

Figure 1S: Variant's sequence comparison

```
w.t._745046         ----AAGGTTTATACCCTCCCAGGTAACAAACCAACCAACTTTCGATCTCTTGTAGATCT   56
Alpha_737204        ATTAAAGGTTTATACCCTCCCAGGTAACAAACCAACCAACTTTCGATCTCTTGTAGATCT   60
Delta_2183060       --TAAAGGTTTATACCCTCCCAGGTAACAAACCAACCAACTTTCGATCTCTTGTAGATCT   58
Omicron_7869197     --TAAAGGTTTATACCCTCCTAGGTAACAAACCAACCAACTTTCGATCTCTTGTAGATCT   58
                        **************** ***************************************

w.t._745046         GTTCTCTAAACGAACTTTAAAATCTGTGTGGCTGTCACTCGGCTGCATGCTTAGTGCACT   116
Alpha_737204        GTTCTCTAAACGAACTTTAAAATCTGTGTGGCTGTCACTCGGCTGCATGCTTAGTGCACT   120
Delta_2183060       GTTCTCTAAACGAACTTTAAAATCTGTGTGGCTGTCACTCGGCTGCATGCTTAGTGCACT   118
Omicron_7869197     GTTCTCTAAACGAACTTTAAAATCTGTGTGGCTGTCACTCGGCTGCATGCTTAGTGCACT   118
                    ************************************************************

w.t._745046         CACGCAGTATAATTAATAACTAATTACTGTCGTTGACAGGACACGAGTAACTCGTCTATC   176
Alpha_737204        CACGCAGTATAATTAATAACTAATTACTGTCGTTGACAGGATACGAGTAACTCGTCTATC   180
Delta_2183060       CACGCAGTATAATTAATAACTAATTACTGTCGTTGACAGGACACGAGTAACTCGTCTATC   178
Omicron_7869197     CACGCAGTATAATTAATAACTAATTACTGTCGTTGACAGGACACGAGTAACTCGTCTATC   178
                    ***************************************** ******************

w.t._745046         TTCTGCAGGCTGCTTACGGTTTCGTCCGTGTTGCAGCCGATCATCAGCACATCTAGGTTT   236
Alpha_737204        TTCTGCAGGCTGCTTACGGTTTCGTCCGTGTTGCAGCCGATCATCAGCACATCTAGGTTT   240
Delta_2183060       TTCTGCAGGCTGCTTACGGTTTCGTCCGTTTTGCAGCCGATCATCAGCACATCTAGGTTT   238
Omicron_7869197     TTCTGCAGGCTGCTTACGGTTTCGTCCGTGTTGCAGCCGATCATCAGCACATCTAGGTTT   238
                    ***************************** ******************************

w.t._745046         TGTCCGGGTGTGACCGAAAGGTAAGATGGAGAGCCTTGTCCCTGGTTTCAACGAGAAAAC   296
Alpha_737204        TGTCCGGGTGTGACCGAAAGGTAAGATGGAGAGCCTTGTCCCTGGTTTCAACGAGAAAAC   300
Delta_2183060       TGTCCGGGTGTGACCGAAAGGTAAGATGGAGAGCCTTGTCCCTGGTTTCAACGAGAAAAC   298
Omicron_7869197     TGTCCGGGTGTGACCGAAAGGTAAGATGGAGAGCCTTGTCCCTGGTTTCAACGAGAAAAC   298
                    ************************************************************

w.t._745046         ACACGTCCAACTTAGTTTGCCTGTTTTACAGGTTCGCGACGTGCTCGTACGTGGCTTTGG   356
Alpha_737204        ACACGTCCAACTCAGTTTGCCTGTTTTACAGGTTCGCGACGTGCTCGTACGTGGCTTTGG   360
Delta_2183060       ACACGTCCAACTCAGTTTGCCTGTTTTACAGGTTCGCGACGTGCTCGTACGTGGCTTTGG   358
Omicron_7869197     ACACGTCCAACTCAGTTTGCCTGTTTTACAGGTTCGCGACGTGCTCGTACGTGGCTTTGG   358
                    ************ ***********************************************

w.t._745046         AGACTCCGTGGAGGAGGTCTTATCAGAGGCACGTCAACATCTTAAAGATGGCACTTGTGG   416
Alpha_737204        AGACTCCGTGGAGGAGGTCTTATCAGAGGCACGTCAACATCTTAAAGATGGCACTTGTGG   420
Delta_2183060       AGACTCCGTGGAGGAGGTCTTATCAGAGGCACGTCAACATCTTAAAGATGGCACTTGTGG   418
Omicron_7869197     AGACTCCGTGGAGGAGGTCTTATCAGAGGCACGTCAACATCTTAAAGATGGCACTTGTGG   418
                    ************************************************************

w.t._745046         CTTAGTAGAAGTTGAAAAAGGCGTTTTGCCTCAACTTGAACAGCCCTATGTGTTCATCAA   476
Alpha_737204        CTTAGTAGAAGTTGAAAAAGGCGTTTTGCCTCAACTTGAACAGCCCTATGTGTTCATCAA   480
Delta_2183060       CTTAGTAGAAGTTGAAAAAGGCGTTTTGCCTCAACTTGAACAGCCCTATGTGTTCATCAA   478
Omicron_7869197     CTTAGTAGAAGTTGAAAAAGGCGTTTTGCCTCAACTTGAACAGCCCTATGTGTTCATCAA   478
                    ************************************************************

w.t._745046         ACGTTCGGATGCTCGAACTGCACCTCATGGTCATGTTATGGTTGAGCTGGTAGCAGAACT   536
Alpha_737204        ACGTTCGGATGCTCGAACTGCACCTCATGGTCATGTTATGGTTGAGCTGGTAGCAGAACT   540
Delta_2183060       ACGTTCGGATGCTCGAACTGCACCTCATGGTCATGTTATGGTTGAGCTGGTAGCAGAACT   538
Omicron_7869197     ACGTTCGGATGCTCGAACTGCACCTCATGGTCATGTTATGGTTGAGCTGGTAGCAGAACT   538
                    ************************************************************

w.t._745046         CGAAGGCATTCAGTACGGTCGTAGTGGTGAGACACTTGGTGTCCTTGTCCCTCATGTGGG   596
Alpha_737204        CGAAGGCATTCAGTACGGTCGTAGTGGTGAGACACTTGGTGTCCTTGTCCCTCATGTGGG   600
Delta_2183060       CGAAGGCATTCAGTACGGTCGTAGTGGTGAGACACTTGGTGTCCTTGTCCCTCATGTGGG   598
Omicron_7869197     CGAAGGCATTCAGTACGGTCGTAGTGGTGAGACACTTGGTGTCCTTGTCCCTCATGTGGG   598
                    ************************************************************
```

```
w.t._745046        CGAAATACCAGTGGCTTACCGCAAGGTTCCTCTTCGTAAGAACGGTAATAAAGGAGCTGG   656
Alpha_737204       CGAAATACCAGTGGCTTACCGCAAGGTTCCTCTTCGTAAGAACGGTAATAAAGGAGCTGG   660
Delta_2183060      CGAAATACCAGTGGCTTACCGCAAGGTTCCTCTTCGTAAGAACGGTAATAAAGGAGCTGG   658
Omicron_7869197    CGAAATACCAGTGGCTTACCGCAAGGTTCCTCTTCGTAAGAACGGTAATAAAGGAGCTGG   658
                   ************************************************************

w.t._745046        TGGCCATAGTTACGGCGCCGATCTAAAGTCATTTGACTTAGGCGACGAGCTTGGCACTGA   716
Alpha_737204       TGGCCATAGTTACGGCGCCGATCTAAAGTCATTTGACTTAGGCGACGAGCTTGGCACTGA   720
Delta_2183060      TGGCCATAGTTACGGCGCCGATCTA---------GACTTAGGCGACGAGCTTGGCACTGA   709
Omicron_7869197    TGGCCATAGTTACGGCGCCGATCTAAAGTCATTTGACTTAGGCGACGAGCTTGGCACTGA   718
                   *************************         **************************

w.t._745046        TCCTTATGAAGATTTTCAAGAAAACTGGAACACTAAACATAGCAGTGGTGTTACCCGTGA   776
Alpha_737204       TCCTTATGAAGATTTTCAAGAAAACTGGAACACTAAACATAGCAGTGGTGTTACCCGTGA   780
Delta_2183060      TCCTTATGAAGATTTTCAAGAAAACTGGAACACTAAACATAGCAGTGGTGTTACCCGTGA   769
Omicron_7869197    TCCTTATGAAGATTTTCAAGAAAACTGGAACACTAAACATAGCAGTGGTGTTACCCGTGA   778
                   ************************************************************

w.t._745046        ACTCATGCGTGAGCTTAACGGAGGGGCATACACTCGCTATGTCGATAACAACCTCTGTGG   836
Alpha_737204       ACTCATGCGTGAGCTTAACGGAGGGGCATACACTCGCTATGTCGATAACAACCTCTGTGG   840
Delta_2183060      ACTCATGCGTGAGCTTAACGGAGGGGCATACACTCGCTATGTCGATAACAACCTCTGTGG   829
Omicron_7869197    ACTCATGCGTGAGCTTAACGGAGGGGCATACACTCGCTATGTCGATAACAACCTCTGTGG   838
                   ************************************************************

w.t._745046        CCCTGATGGCTACCCTCTTGAGTGCATTAAAGACCCTCTAGCACGTGCTGGTAAAGCCTC   896
Alpha_737204       CCCTGATGGCTACCCTCTTGAGTGCATTAAAGACCCTCTAGCACGTGCTGGTAAAGCCTC   900
Delta_2183060      CCCTGATGGCTACCCTCTTGAGTGCATTAAAGACCCTCTAGCACGTGCTGGTAAAGCCTC   889
Omicron_7869197    CCCTGATGGCTACCCTCTTGAGTGCATTAAAGACCCTCTAGCACGTGCTGGTAAAGCCTC   898
                   ************************************************************

w.t._745046        ATGCACTTTGTCCGAACAACTGGACTTTATTGACACTAAGAGGGGTGTATACTGCTGCCG   956
Alpha_737204       ATGCACTTTGTCTGAACAACTGGACTTTATTGACACTAAGAGGGGTGTATACTGCTGCCG   960
Delta_2183060      ATGCACTTTGTCCGAACAACTGGACTTTATTGACACTAAGAGGGGTGTATACTGCTGCCG   949
Omicron_7869197    ATGCACTTTGTCCGAACAACTGGACTTTATTGACACTAAGAGGGGTGTATACTGCTGCCG   958
                   ************ ***********************************************

w.t._745046        TGAACATGAGCATGAAATTGCTTGGTACACGGAACGTTCTGAAAAGAGCTATGAATTGCA   1016
Alpha_737204       TGAACATGAGCATGAAATTGCTTGGTACACGGAACGTTCTGAAAAGAGCTATGAATTGCA   1020
Delta_2183060      TGAACATGAGCATGAAATTGCTTGGTACACGGAACGTTCTGAAAAGAGCTATGAATTGCA   1009
Omicron_7869197    TGAACATGAGCATGAAATTGCTTGGTACACGGAACGTTCTGAAAAGAGCTATGAATTGCA   1018
                   ************************************************************

w.t._745046        GACACCTTTTGAAATTAAATTGGCAAAGAAATTTGACACCCTCAATGGGGAATGTCCAAA   1076
Alpha_737204       GACACCTTTTGAAATTAAATTGGCAAAGAAATTTGACACCCTCAATGGGGAATGTCCAAA   1080
Delta_2183060      GACACCTTTTGAAATTAAATTGGCAAAGAAATTTGACACCCTCAATGGGGAATGTCCAAA   1069
Omicron_7869197    GACACCTTTTGAAATTAAATTGGCAAAGAAATTTGACACCCTCAATGGGGAATGTCCAAA   1078
                   ************************************************************

w.t._745046        TTTTGTATTTCCCTTAAATTCCATAATCAAGACTATTCAACCAAGGGTTGAAAAGAAAAA   1136
Alpha_737204       TTTTGTATTTCCCTTAAATTCCATAATCAAGACTATTCAACCAAGGGTTGAAAAGAAAAA   1140
Delta_2183060      TTTTGTATTTCCCTTAAATTCCATAATCAAGACTATTCAACCAAGGGTTGAAAAGAAAAA   1129
Omicron_7869197    TTTTGTATTTCCCTTAAATTCCATAATCAAGACTATTCAACCAAGGGTTGAAAAGAAAAA   1138
                   ************************************************************

w.t._745046        GCTTGATGGCTTTATGGGTAGAATTCGATCTGTCTATCCAGTTGCGTCACTAAATGAATG   1196
Alpha_737204       GCTTGATGGCTTTATGGGTAGAATTCGATCTGTCTATCCAGTTGCGTCACCAAATGAATG   1200
Delta_2183060      GCTTGATGGCTTTATGGGTAGAATTCGATCTGTCTATCCAGTTGCGTCACTAAATGAATG   1189
Omicron_7869197    GCTTGATGGCTTTATGGGTAGAATTCGATCTGTCTATCCAGTTGCGTCACCAAATGAATG   1198
                   ************************************************** *********
```

```
w.t._745046         CAACCAAATGTGCCTTTCAACTCTCATGAAGTGTGATCATTGTGGTGAAACCTCATGGCA   1256
Alpha_737204        CAACCAAATGTGCCTTTCAACTCTCATGAAGTGTGATCATTGTGGTGAAACCTCATGGCA   1260
Delta_2183060       CAACCAAATGTGCCTTTCAACTCTCATGAAGTGTGATCATTGTGGTGAAACCTCATGGCA   1249
Omicron_7869197     CAACCAAATGTGCCTTTCAACTCTCATGAAGTGTGATCATTGTGGTGAAACCTCATGGCA   1258
                    ************************************************************

w.t._745046         GACGGGCGATTTTGTTAAAGCCACTTGCGAATTTTGTGGCACTGAGAATTTGACTAAAGA   1316
Alpha_737204        GACGGGCGATTTTGTTAAAGCCACTTGCGAATTTTGTGGCACTGAGAATTTGACTAAAGA   1320
Delta_2183060       GACGGGTGATTTTGTTAAAGCCACTTGCGAATTTTGTGGCACTGAGAATTTGACTAAAGA   1309
Omicron_7869197     GACGGGCGATTTTGTTAAAGCCACTTGCGAATTTTGTGGCACTGAGAATTTGACTAAAGA   1318
                    ****** *****************************************************

w.t._745046         AGGTGCCACTACTTGTGGTTACTTACCCCAAAATGCTGTTGTTAAAATTTATTGTCCAGC   1376
Alpha_737204        AGGTGCCACTACTTGTGGTTACTTACCCCAAAATGCTGTTGTTAAAATTTATTGTCCAGC   1380
Delta_2183060       AGGTGCCACTACTTGTGGTTACTTACCCCAAAATGCTGTTGTTAAAATTTATTGTCCAGC   1369
Omicron_7869197     AGGTGCCACTACTTGTGGTTACTTACCCCAAAATGCTGTTGTTAAAATTTATTGTCCAGC   1378
                    ************************************************************

w.t._745046         ATGTCACAATTCAGAAGTAGGACCTGAGCATAGTCTTGCCGAATACCATAATGAATCTGG   1436
Alpha_737204        ATGTCACAATTCAGAAGTAGGACCTGAGCATAGTCTTGCCGAATACCATAATGAATCTGG   1440
Delta_2183060       ATGTCACAATTCAGAAGTAGGACCTGAGCATAGTCTTGCCGAATACCATAATGAATCTGG   1429
Omicron_7869197     ATGTCACAATTCAGAAGTAGGACCTGAGCATAGTCTTGCCGAATACCATAATGAATCTGG   1438
                    ************************************************************

w.t._745046         CTTGAAAACCATTCCTCGTAAGGGTGGTCGCACTATTGCCTTTGGAGGCTGTGTGTTCTC   1496
Alpha_737204        CTTGAAAACCATTCCTCGTAAGGGTGGTCGCACTATTGCCTTTGGAGGCTGTGTGTTCTC   1500
Delta_2183060       CTTGAAAACCATTCCTCGTAAGGGTGGTCGCACTATTGCCTTTGGAGGCTGTGTGTTCTC   1489
Omicron_7869197     CTTGAAAACCATTCCTCGTAAGGGTGGTCGCACTATTGCCTTTGGAGGCTGTGTGTTCTC   1498
                    ************************************************************

w.t._745046         TTATGTTGGTTGCCATAACAAGTGTGCCTATTGGGTTCCACGTGCTAGCGCTAACATAGG   1556
Alpha_737204        TTATGTTGGTTGCCATAACAAGTGTGCCTATTGGGTTCCACGTGCTAGCGCTAACATAGG   1560
Delta_2183060       TTATGTTGGTTGCCATAACAAGTGTGCCTATTGGGTTCCACGTGCTAGCGCTAACATAGG   1549
Omicron_7869197     TTATGTTGGTTGCCATAACAAGTGTGCCTATTGGGTTCCACGTGCTAGCGCTAACATAGG   1558
                    ************************************************************

w.t._745046         TTGTAACCATACAGGTGTTGTTGGAGAAGGTTCCGAAGGTCTTAATGACAACCCTCTTGA   1616
Alpha_737204        TTGTAACCATACAGGTGTTGTTGGAGAAGGTTCCGAAGGTCTTAATGACAACCCTCTTGA   1620
Delta_2183060       TTGTAACCATACAGGTGTTGTTGGAGAAGGTTCCGAAGGTCTTAATGACAACCCTCTTGA   1609
Omicron_7869197     TTGTAACCATACAGGTGTTGTTGGAGAAGGTTCCGAAGGTCTTAATGACAACCCTCTTGA   1618
                    ************************************************************

w.t._745046         AATACTCCAAAAAGAGAAAGTCAACATCAATATTGTTGGTGACTTTAAACTTAATGAAGA   1676
Alpha_737204        AATACTCCAAAAAGAGAAAGTCAACATCAATATTGTTGGTGACTTTAAACTTAATGAAGA   1680
Delta_2183060       AATACTCCAAAAAGAGAAAGTCAACATCAATATTGTTGGTGACTTTAAACTTAATGAAGA   1669
Omicron_7869197     AATACTCCAAAAAGAGAAAGTCAACATCAATATTGTTGGTGACTTTAAACTTAATGAAGA   1678
                    ************************************************************

w.t._745046         GATCGCCATTATTTTGGCATCTTTTTCTGCCTCCACAAGTGCTTTTGTGGAAACTGTGAA   1736
Alpha_737204        GATCGCCATTATTTTGGCATCTTTTTCTGCCTCCACAAGTGCTTTTGTGGAAACTGTGAA   1740
Delta_2183060       GATCGCCATTATTTTGGCATCTTTTTCTGCCTCCACAAGTGCTTTTGTGGAAACTGTGAA   1729
Omicron_7869197     GATCGCCATTATTTTGGCATCTTTTTCTGCCTCCACAAGTGCTTTTGTGGAAACTGTGAA   1738
                    ************************************************************

w.t._745046         AGGTTTGGATTATAAAGCATTCAAACAAATTGTTGAATCCTGTGGTAATTTTAAAGTTAC   1796
Alpha_737204        AGGTTTGGATTATAAAGCATTCAAACAAATTGTTGAATCCTGTGGTAATTTTAAAGTTAC   1800
Delta_2183060       AGGTTTGGATTATAAAGCATTCAAACAAATTGTTGAATCCTGTGGTAATTTTAAAGTTAC   1789
Omicron_7869197     AGGTTTGGATTATAAAGCATTCAAACAAATTGTTGAATCCTGTGGTAATTTTAAAGTTAC   1798
                    ************************************************************
```

```
w.t._745046         AAAAGGAAAAGCTAAAAAAGGTGCCTGGAATATTGGTGAACAGAAATCAATACTGAGTCC   1856
Alpha_737204        AAAAGGAAAAGCTAAAAAAGGTGCCTGGAATATTGGTGAACAGAAATCAATACTGAGTCC   1860
Delta_2183060       AAAAGGAAAAGCTAAAAAAGGTGCCTGGAATATTGGTGAACAGAAATCAATACTGAGTCC   1849
Omicron_7869197     AAAAGGAAAAGCTAAAAAAGGTGCCTGGAATATTGGTGAACAGAAATCAATACTGAGTCC   1858
                    ************************************************************

w.t._745046         TCTTTATGCATTTGCATCAGAGGCTGCTCGTGTTGTACGATCAATTTTCTCCCGCACTCT   1916
Alpha_737204        TCTTTATGCATTTGCATCAGAGGCTGCTCGTGTTGTACGATCAATTTTCTCCCGCACTCT   1920
Delta_2183060       TCTTTATGCATTTGCATCAGAGGCTGCTCGTGTTGTACGATCAATTTTCTCCCGCACTCT   1909
Omicron_7869197     TCTTTATGCATTTGCATCAGAGGCTGCTCGTGTTGTACGATCAATTTTCTCCCGCACTCT   1918
                    ************************************************************

w.t._745046         TGAAACTGCTCAAAATTCTGTGCGTGTTTTACAGAAGGCCGCTATAACAATACTAGATGG   1976
Alpha_737204        TGAAACTGCTCAAAATTCTGTGCGTGTTTTACAGAAGGCCGCTATAACAATACTAGATGG   1980
Delta_2183060       TGAAACTGCTCAAAATTCTGTGCGTGTTTTACAGAAGGCCGCTATAACAATACTAGATGG   1969
Omicron_7869197     TGAAACTGCTCAAAATTCTGTGCGTGTTTTACAGAATGCCGCTATAACAATACTAGATGG   1978
                    ************************************ ***********************

w.t._745046         AATTTCACAGTATTCACTGAGACTCATTGATGCTATGATGTTCACATCTGATTTGGCTAC   2036
Alpha_737204        AATTTCACAGTATTCACTGAGACTCATTGATGCTATGATGTTCACATCTGATTTGGCTAC   2040
Delta_2183060       AATTTCACAGTATTCACTGAGACTCATTGATGCTATGATGTTCACATCTGATTTGGCTAC   2029
Omicron_7869197     AATTTCACAGTATTCACTGAGACTCATTGATGCTATGATGTTCACATCTGATTTGGCTAC   2038
                    ************************************************************

w.t._745046         TAACAATCTAGTTGTAATGGCCTACATTACAGGTGGTGTTGTTCAGTTGACCTCGCAGTG   2096
Alpha_737204        TAACAATCTAGTTGTAATGGCCTACATTACAGGTGGTGTTGTTCAGTTGACCTCGCAGTG   2100
Delta_2183060       TAACAATCTAGTTGTAATGGCCTACATTACAGGTGGTGTTGTTCAGTTGACCTCGCAGTG   2089
Omicron_7869197     TAACAATCTAGTTGTAATGGCCTACATTACAGGTGGTGTTGTTCAGTTGACCTCGCAGTG   2098
                    ************************************************************

w.t._745046         GCTAACTAACATCTTTGGCACTGTTTATGAAAAACTCAAACCCGTCCTTGATTGGCTTGA   2156
Alpha_737204        GCTAACTAACATCTTTGGCACTGTTTATGAAAAACTCAAACCCGTCCTTGATTGGCTTGA   2160
Delta_2183060       GCTAACTAACATCTTTGGCACTGTTTATGAAAAACTCAAACCCGTCCTTGATTGGCTTGA   2149
Omicron_7869197     GCTAACTAACATCTTTGGCACTGTTTATGAAAAACTCAAACCCGTCCTTGATTGGCTTGA   2158
                    ************************************************************

w.t._745046         AGAGAAGTTTAAGGAAGGTGTAGAGTTTCTTAGAGACGGTTGGGAAATTGTTAAATTTAT   2216
Alpha_737204        AGAGAAGTTTAAGGAAGGTGTAGAGTTTCTTAGAGACGGTTGGGAAATTGTTAAATTTAT   2220
Delta_2183060       AGAGAAGTTTAAGGAAGGTGTAGAGTTTCTTAGAGACGGTTGGGAAATTGTTAAATTTAT   2209
Omicron_7869197     AGAGAAGTTTAAGGAAGGTGTAGAGTTTCTTAGAGACGGTTGGGAAATTGTTAAATTTAT   2218
                    ************************************************************

w.t._745046         CTCAACCTGTGCTTGTGAAATTGTCGGTGGACAAATTGTCACCTGTGCAAAGGAAATTAA   2276
Alpha_737204        CTCAACCTGTGCTTGTGAAATTGTCGGTGGACAAATTGTCACCTGTGCAAAGGAAATTAA   2280
Delta_2183060       CTCAACCTGTGCTTGTGAAATTGTCGGTGGACAAATTGTCACCTGTGCAAAGGAAATTAA   2269
Omicron_7869197     CTCAACCTGTGCTTGTGAAATTGTCGGTGGACAAATTGTCACCTGTGCAAAGGAAATTAA   2278
                    ************************************************************

w.t._745046         GGAGAGTGTTCAGACATTCTTTAAGCTTGTAAATAAATTTTTGGCTTTGTGTGCTGACTC   2336
Alpha_737204        GGAGAGTGTTCAGACATTCTTTAAGCTTGTAAATAAATTTTTGGCTTTGTGTGCTGACTC   2340
Delta_2183060       GGAGAGTGTTCAGACATTCTTTAAGCTTGTAAATAAATTTTTGGCTTTGTGTGCTGACTC   2329
Omicron_7869197     GGAGAGTGTTCAGACATTCTTTAAGCTTGTAAATAAATTTTTGGCTTTGTGTGCTGACTC   2338
                    ************************************************************

w.t._745046         TATCATTATTGGTGGAGCTAAACTTAAAGCCTTGAATTTAGGTGAAACATTTGTCACGCA   2396
Alpha_737204        TATCATTATTGGTGGAGCTAAACTTAAAGCCTTGAATTTAGGTGAAACATTTGTCACGCA   2400
Delta_2183060       TATCATTATTGGTGGAGCTAAACTTAAAGCCTTGAATTTAGGTGAAACATTTGTCACGCA   2389
Omicron_7869197     TATCATTATTGGTGGAGCTAAACTTAAAGCCTTGAATTTAGGTGAAACATTTGTCACGCA   2398
                    ************************************************************
```

```
w.t._745046         CTCAAAGGGATTGTACAGAAAGTGTGTTAAATCCAGAGAAGAAACTGGCCTACTCATGCC   2456
Alpha_737204        CTCAAAGGGATTGTACAGAAAGTGTGTTAAATCCAGAGAAGAAACTGGCCTACTCATGCC   2460
Delta_2183060       CTCAAAGGGATTGTACAGAAAGTGTGTTAAATCCAGAGAAGAAACTGGCCTACTCATGCC   2449
Omicron_7869197     CTCAAAGGGATTGTACAGAAAGTGTGTTAAATCCAGAGAAGAAACTGGCCTACTCATGCC   2458
                    ************************************************************

w.t._745046         TCTAAAAGCCCCAAAAGAAATTATCCTCTTAGAGGGAGAAACACCTCCCACAGAAGTGTT   2516
Alpha_737204        TCTAAAAGCCCCAAAAGAAATTATCCTCTTAGAGGGAGAAACACCTCCCACAGAAGTGTT   2520
Delta_2183060       TCTAAAAGCCCCAAAAGAAATTATCCTCTTAGAGGGAGAAACACCTCCCACAGAAGTGTT   2509
Omicron_7869197     TCTAAAAGCCCCAAAAGAAATTATCCTCTTAGAGGGAGAAACACCTCCCACAGAAGTGTT   2518
                    ************************************************************

w.t._745046         AACAGAGGAAGTTGTCTTGAAAACTGGTGATTTACAACCATTAGAACAACCTACTAGTGA   2576
Alpha_737204        AACAGAGGAAGTTGTCTTGAAAACTGGTGATTTACAACCATTAGAACAACCTACTAGTGA   2580
Delta_2183060       AACAGAGGAAGTTGTCTTGAAAACTGGTGATTTACAACCATTAGAACAACCTACTAGTGA   2569
Omicron_7869197     AACAGAGGAAGTTGTCTTGAAAACTGGTGATTTACAACCATTAGAACAACCTACTAGTGA   2578
                    ************************************************************

w.t._745046         AGCTGTTGAAGCTCCATTGGTTGGTACACCAGTTTGTATTAACGGGCTTATGTTGCTCGA   2636
Alpha_737204        AGCTGTTGAAGCTCCATTGGTTGGTACACCAGTTTGTATTAACGGGCTTATGTTGCTCGA   2640
Delta_2183060       AGCTGTTGAAGCTCCATTGGTTGGTACACCAGTTTGTATTAACGGGCTTATGTTGCTCGA   2629
Omicron_7869197     AGCTGTTGAAGCTCCATTGGTTGGTACACCAGTTTGTATTAACGGGCTTATGTTGCTCGA   2638
                    ************************************************************

w.t._745046         AATCAAAGACACAGAAAAGTACTGTGCCCTTGCACCTAATATGATGGTAACAAACAATAC   2696
Alpha_737204        AATCAAAGACACAGAAAAGTACTGTGCCCTTGCACCTAATATGATGGTAACAAACAATAC   2700
Delta_2183060       AATCAAAGACACAGAAAAGTACTGTGCCCTTGCACCTAATATGATGGTAACAAACAATAC   2689
Omicron_7869197     AATCAAAGACACAGAAAAGTACTGTGCCCTTGCACCTAATATGATGGTAACAAACAATAC   2698
                    ************************************************************

w.t._745046         CCTCACACTCAAAGGCGGTGCACCAACAAAGGTTACTTTTGGTGATGACACTGTGATAGA   2756
Alpha_737204        CCTCACACTCAAAGGCGGTGCACCAACAAAGGTTACTTTTGGTGATGACACTGTGATAGA   2760
Delta_2183060       CCTCACACTCAAAGGCGGTGCACCAACAAAGGTTACTTTTGGTGATGACACTGTGATAGA   2749
Omicron_7869197     CCTCACACTCAAAGGCGGTGCACCAACAAAGGTTACTTTTGGTGATGACACTGTGATAGA   2758
                    ************************************************************

w.t._745046         AGTGCAAGGTTACAAGAGTGTGAATATCACTTTTGAACTTGATGAAAGGATTGATAAAGT   2816
Alpha_737204        AGTGCAAGGTTACAAGAGTGTGAATATCACTTTTGAACTTGATGAAAGGATTGATAAAGT   2820
Delta_2183060       AGTGCAAGGTTACAAGAGTGTGAATATCACTTTTGAACTTGATGAAAGGATTGATAAAGT   2809
Omicron_7869197     AGTGCAAGGTTACAAGAGTGTGAATATCACTTTTGAACTTGATGAAAGGATTGATAAAGT   2818
                    ************************************************************

w.t._745046         ACTTAATGAGAAGTGCTCTGCCTATACAGTTGAACTCGGTACAGAAGTAAATGAGTTCGC   2876
Alpha_737204        ACTTAATGAGAAGTGCTCTGCCTATACAGTTGAACTCGGTACAGAAGTAAATGAGTTCGC   2880
Delta_2183060       ACTTAATGAGAAGTGCTCTGCCTATACAGTTGAACTCGGTACAGAAGTAAATGAGTTCGC   2869
Omicron_7869197     ACTTAATGAGAGGTGCTCTGCCTATACAGTTGAACTCGGTACAGAAGTAAATGAGTTCGC   2878
                    *********** ************************************************

w.t._745046         CTGTGTTGTGGCAGATGCTGTCATAAAAACTTTGCAACCAGTATCTGAATTACTTACACC   2936
Alpha_737204        CTGTGTTGTGGCAGATGCTGTCATAAAAACTTTGCAACCAGTATCTGAATTACTTACACC   2940
Delta_2183060       CTGTGTTGTGGCAGATGCTGTCATAAAAACTTTGCAACCAGTATCTGAATTACTTACACC   2929
Omicron_7869197     CTGTGTTGTGGCAGATGCTGTCATAAAAACTTTGCAACCAGTATCTGAATTACTTACACC   2938
                    ************************************************************

w.t._745046         ACTGGGCATTGATTTAGATGAGTGGAGTATGGCTACATACTACTTATTTGATGAGTCTGG   2996
Alpha_737204        ACTGGGCATTGATTTAGATGAGTGGAGTATGGCTACATACTACTTATTTGATGAGTCTGG   3000
Delta_2183060       ACTGGGCATTGATTTAGATGAGTGGAGTATGGCTACATACTACTTATTTGATGAGTCTGG   2989
Omicron_7869197     ACTGGGCATTGATTTAGATGAGTGGAGTATGGCTACATACTACTTATTTGATGAGTCTGG   2998
                    ************************************************************
```

```
w.t._745046         TGAGTTTAAATTGGCCTCACATATGTATTGTTCTTTTTACCCTCCAGATGAGGATGAAGA   3056
Alpha_737204        TGAGTTTAAATTGGCCTCACATATGTATTGTTCTTTTTACCCTCCAGATGAGGATGAAGA   3060
Delta_2183060       TGAGTTTAAATTGGCCTCACATATGTATTGTTCTTTTTACCCTCCAGATGAGGATGAAGA   3049
Omicron_7869197     TGAGTTTAAATTGGCCTCACATATGTATTGTTCTTTTTACCCTCCAGATGAGGATGAAGA   3058
                    ************************************************************

w.t._745046         AGAAGGTGATTGTGAAGAAGAAGAGTTTGAGCCATCAACTCAATATGAGTATGGTACTGA   3116
Alpha_737204        AGAAGGTGATTGTGAAGAAGAAGAGTTTGAGCCATCAACTCAATATGAGTATGGTACTGA   3120
Delta_2183060       AGAAGGTGATTGTGAAGAAGAAGAGTTTGAGCCATCAACTCAATATGAGTATGGTACTGA   3109
Omicron_7869197     AGAAGGTGATTGTGAAGAAGAAGAGTTTGAGCCATCAACTCAATATGAGTATGGTACTGA   3118
                    ************************************************************

w.t._745046         AGATGATTACCAAGGTAAACCTTTGGAATTTGGTGCCACCTCTGCTGCTCCTCAACCTGA   3176
Alpha_737204        AGATGATTACCAAGGTAAACCTTTGGAATTTGGTGCCACCTCTGCTGCTCCTCAACCTGA   3180
Delta_2183060       AGATGATTACCAAGGTAAACCTTTGGAATTTGGTGCCACCTCTGCTGCTCCTCAACCTGA   3169
Omicron_7869197     AGATGATTACCAAGGTAAACCTTTGGAATTTGGTGCCACCTCTGCTGCTCCTCAACCTGA   3178
                    ************************************************************

w.t._745046         AGAAGAGCAAGAAGAAGATTGGTTAGATGATGATAGTCAACAAACTGTTGGTCAACAAGA   3236
Alpha_737204        AGAAGAGCAAGAAGAAGATTGGTTAGATGATGATAGTCAACAAACTGTTGGTCAACAAGA   3240
Delta_2183060       AGAAGAGCAAGAAGAAGATTGGTTAGATGATGATAGTCAACAAACTGTTGGTCAACAAGA   3229
Omicron_7869197     AGAAGAGCAAGAAGAAGATTGGTTAGATGATGATAGTCAACAAACTGTTGGTCAACAAGA   3238
                    ************************************************************

w.t._745046         CGGCAGTGAGGACAATCAGACAACTACTATTCAAACAATTGTTGAGGTTCAACCTCAATT   3296
Alpha_737204        CGGCAGTGAGGACAATCAGACAACTATTATTCAAACAATTGTTGAGGTTCAACCTCAATT   3300
Delta_2183060       CGGCAGTGAGGACAATCAGACAACTACTATTCAAACAATTGTTGAGGTTCAACCTCAATT   3289
Omicron_7869197     CGGCAGTGAGGACAATCAGACAACTACTATTCAAACAATTGTTGAGGTTCAACCTCAATT   3298
                    ************************** *********************************

w.t._745046         AGAGATGGAACTTACACCAGTTGTTCAGACTATTGAAGTGAATAGTTTTAGTGGTTATTT   3356
Alpha_737204        AGAGATGGAACTTACACCAGTTGTTCAGACTATTGAAGTGAATAGTTTTAGTGGTTATTT   3360
Delta_2183060       AGAGATGGAACTTACACCAGTTGTTCAGACTATTGAAGTGAATAGTTTTAGTGGTTATTT   3349
Omicron_7869197     AGAGATGGAACTTACACCAGTTGTTCAGACTATTGAAGTGAATAGTTTTAGTGGTTATTT   3358
                    ************************************************************

w.t._745046         AAAACTTACTGACAATGTATACATTAAAAATGCAGACATTGTGGAAGAAGCTAAAAAGGT   3416
Alpha_737204        AAAACTTACTGACAATGTATACATTAAAAATGCAGACATTGTGGAAGAAGCTAAAAAGGT   3420
Delta_2183060       AAAACTTACTGACAATGTATACATTAAAAATGCAGACATTGTGGAAGAAGCTAAAAAGGT   3409
Omicron_7869197     AAAACTTACTGACAATGTATACATTAAAAATGCAGACATTGTGGAAGAAGCTAAAAAGGT   3418
                    ************************************************************

w.t._745046         AAAACCAACAGTGGTTGTTAATGCAGCCAATGTTTACCTTAAACATGGAGGAGGTGTTGC   3476
Alpha_737204        AAAACCAACAGTGGTTGTTAATGCAGCCAATGTTTACCTTAAACATGGAGGAGGTGTTGC   3480
Delta_2183060       AAAACCAACAGTGGTTGTTAATGCAGCCAATGTTTACCTTAAACATGGAGGAGGTGTTGC   3469
Omicron_7869197     AAAACCAACAGTGGTTGTTAATGCAGCCAATGTTTACCTTAAACATGGAGGAGGTGTTGC   3478
                    ************************************************************

w.t._745046         AGGAGCCTTAAATAAGGCTACTAACAATGCCATGCAAGTTGAATCTGATGATTACATAGC   3536
Alpha_737204        AGGAGCCTTAAATAAGGCTACTAACAATGCCATGCAAGTTGAATCTGATGATTACATAGC   3540
Delta_2183060       AGGAGCCTTAAATAAGGCTACTAACAATGCCATGCAAGTTGAATCTGATGATTACATAGC   3529
Omicron_7869197     AGGAGCCTTAAATAAGGCTACTAACAATGCCATGCAAGTTGAATCTGATGATTACATAGC   3538
                    ************************************************************

w.t._745046         TACTAATGGACCACTTAAAGTGGGTGGTAGTTGTGTTTTAAGCGGACACAATCTTGCTAA   3596
Alpha_737204        TACTAATGGACCACTTAAAGTGGGTGGTAGTTGTGTTTTAAGCGGACACAATCTTGCTAA   3600
Delta_2183060       TACTAATGGACCACTTAAAGTGGGTGGTAGTTGTGTTTTAAGCGGACACAATCTTGCTAA   3589
Omicron_7869197     TACTAATGGACCACTTAAAGTGGGTGGTAGTTGTGTTTTAAGCGGACACAATCTTGCTAA   3598
                    ************************************************************
```

```
w.t._745046        ACACTGTCCTCATGTTGTCGGCCCAAATGTTAACAAAGGTGAAGACATTCAACCTCTTAA   3656
Alpha_737204       ACACTGTCCTCATGTTGTCGGCCCAAATGTTAACAAAGGTGAAGACATTCAACCTCTTAA   3660
Delta_2183060      ACACTGTCCTCATGTTGTCGGCCCAAATGTTAACAAAGGTGAAGACATTCAACCTCTTAA   3649
Omicron_7869197    ACACTGTCCTCATGTTGTCGGCCCAAATGTTAACAAAGGTGAAGACATTCAACCTCTTAA   3658
                   ************************************************************

w.t._745046        GAGTGCTTATGAAAATTTTAATCAGCACGAAGTTCTACTTGCACCATTATTATCAGCTGG   3716
Alpha_737204       GAGTGCTTATGAAAATTTTAATCAGCACGAAGTTCTACTTGCACCATTATTATCAGCTGG   3720
Delta_2183060      GAGTGCTTATGAAAATTTTAATCAGCACGAAGTTCTACTTGCACCATTATTATCAGCTGG   3709
Omicron_7869197    GAGTGCTTATGAAAATTTTAATCAGCACGAAGTTCTACTTGCACCATTATTATCAGCTGG   3718
                   ************************************************************

w.t._745046        TATTTTTGGTGCTGACCCTATACATTCTTTAAGAGTTTGTGTAGATACTGTTCGCACAAA   3776
Alpha_737204       TATTTTTGGTGCTGACCCTATACATTCTTTAAGAGTTTGTGTAGATACTGTTCGCACAAA   3780
Delta_2183060      TATTTTTGGTGCTGACCCTATACATTCTTTAAGAGTTTGTGTAGATACTGTTCGCACAAA   3769
Omicron_7869197    TATTTTTGGTGCTGACCCTATACATTCTTTAAGAGTTTGTGTAGATACTGTTCGCACAAA   3778
                   ************************************************************

w.t._745046        TGTCTACTTAGCTGTCTTTGATAAAAATCTCTATGACAAACTTGTTTCAAGCTTTTTGGA   3836
Alpha_737204       TGTCTACTTAGCTGTCTTTGATAAAAATCTCTATGACAAACTTGTTTCAAGCTTTTTGGA   3840
Delta_2183060      TGTCTACTTAGCTGTCTTTGATAAAAATCTCTATGACAAACTTGTTTCAAGCTTTTTGGA   3829
Omicron_7869197    TGTCTACTTAGCTGTCTTTGATAAAAATCTCTATGACAAACTTGTTTCAAGCTTTTTGGA   3838
                   ************************************************************

w.t._745046        AATGAAGAGTGAAAAGCAAGTTGAACAAAAGATCGCTGAGATTCCTAAAGAGGAAGTTAA   3896
Alpha_737204       AATGAAGAGTGAAAAGCAAGTTGAACAAAAGATCGCTGAGATTCCTAAAGAGGAAGTTAA   3900
Delta_2183060      AATGAAGAGTGAAAAGCAAGTTGAACAAAAGATCGCTGAGATTCCTAAAGAGGAAGTTAA   3889
Omicron_7869197    AATGAAGAGTGAAAAGCAAGTTGAACAAAAGATCGCTGAGATTCCTAAAGAGGAAGTTAA   3898
                   ************************************************************

w.t._745046        GCCATTTATAACTGAAAGTAAACCCTCAGTTGAACAGAGAAAACAAGATGATAAGAAAAT   3956
Alpha_737204       GCCATTTATAACTGAAAGTAAACCCTCAGTTGAACAGAGAAAACAAGATGATAAGAAAAT   3960
Delta_2183060      GCCATTTATAACTGAAAGTAAACCCTCAGTTGAACAGAGAAAACAAGATGATAAGAAAAT   3949
Omicron_7869197    GCCATTTATAACTGAAAGTAAACCCTCAGTTGAACAGAGAAAACAAGATGATAAGAAAAT   3958
                   ************************************************************

w.t._745046        CAAAGCTTGTGTTGAAGAAGTTACAACAACTCTGGAAGAAACTAAGTTCCTCACAGAAAA   4016
Alpha_737204       CAAAGCTTGTGTTGAAGAAGTTACAACAACTCTGGAAGAAACTAAGTTCCTCACAGAAAA   4020
Delta_2183060      CAAAGCTTGTGTTGAAGAAGTTACAACAACTCTGGAAGAAACTAAGTTCCTCACAGAAAA   4009
Omicron_7869197    CAAAGCTTGTGTTGAAGAAGTTACAACAACTCTGGAAGAAACTAAGTTCCTCACAGAAAA   4018
                   ************************************************************

w.t._745046        CTTGTTACTTTATATTGACATTAATGGCAATCCTCATCCAGATTCTGCCACTCTTGTTAG   4076
Alpha_737204       CTTGTTACTTTATATTGACATTAATGGCAATCCTCATCCAGATTCTGCCACTCTTGTTAG   4080
Delta_2183060      CTTGTTACTTTATATTGACATTAATGGCAATCCTCATCCAGATTCTGCCACTCTTGTTAG   4069
Omicron_7869197    CTTGTTACTTTATATTGACATTAATGGCAATCCTCATCCAGATTCTGCCACTCTTGTTAG   4078
                   ************************************************************

w.t._745046        TGACATTGACATCACTTTCTTAAAGAAAGATGCTCCATATATAGTGGGTGATGTTGTTCA   4136
Alpha_737204       TGACATTGACATCACTTTCTTAAAGAAAGATGCTCCATATATAGTGGGTGATGTTGTTCA   4140
Delta_2183060      TGACATTGACATCACTTTCTTAAAGAAAGATGCTCCATATATAGTGGGTGATGTTGTTCA   4129
Omicron_7869197    TGACATTGACATCACTTTCTTAAAGAAAGATGCTCCATATATAGTGGGTGATGTTGTTCA   4138
                   ************************************************************

w.t._745046        AGAGGGTGTTTTAACTGCTGTGGTTATACCTACTAAAAAGGCTGGTGGCACTACTGAAAT   4196
Alpha_737204       AGAGGGTGTTTTAACTGCTGTGGTTATACCTACTAAAAAGGCTGGTGGCACTACTGAAAT   4200
Delta_2183060      AGAGGGTGTTTTAACTGCTGTGGTTATACCTACTAAAAAGGCTGGTGGCACTACTGAAAT   4189
Omicron_7869197    AGAGGGTGTTTTAACTGCTGTGGTTATACCTACTAAAAAGGCTGGTGGCACTACTGAAAT   4198
                   ************************************************************
```

```
w.t._745046         GCTAGCGAAAGCTTTGAGAAAAGTGCCAACAGACAATTATATAACCACTTACCCGGGTCA   4256
Alpha_737204        GCTAGCGAAAGCTTTGAGAAAAGTGCCAACAGACAATTATATAACCACTTACCCGGGTCA   4260
Delta_2183060       GCTAGCGAAAGCTTTGAGAAAAGTGCCAACAGACAATTATATAACCACTTACCCGGGTCA   4249
Omicron_7869197     GCTAGCGAAAGCTTTGAGAAAAGTGCCAACAGACAATTATATAACCACTTACCCGGGTCA   4258
                    ************************************************************

w.t._745046         GGGTTTAAATGGTTACACTGTAGAGGAGGCAAAGACAGTGCTTAAAAAGTGTAAAAGTGC   4316
Alpha_737204        GGGTTTAAATGGTTACACTGTAGAGGAGGCAAAGACAGTGCTTAAAAAGTGTAAAAGTGC   4320
Delta_2183060       GGGTTTAAATGGTTACACTGTAGAGGAGGCAAAGACAGTGCTTAAAAAGTGTAAAAGTGC   4309
Omicron_7869197     GGGTTTAAATGGTTACACTGTAGAGGAGGCAAAGACAGTGCTTAAAAAGTGTAAAAGTGC   4318
                    ************************************************************

w.t._745046         CTTTTACATTCTACCATCTATTATCTCTAATGAGAAGCAAGAAATTCTTGGAACTGTTTC   4376
Alpha_737204        CTTTTACATTCTACCATCTATTATCTCTAATGAGAAGCAAGAAATTCTTGGAACTGTTTC   4380
Delta_2183060       CTTTTACATTCTACCATCTATTATCTCTAATGAGAAGCAAGAAATTCTTGGAACTGTTTC   4369
Omicron_7869197     CTTTTACATTCTACCATCTATTATCTCTAATGAGAAGCAAGAAATTCTTGGAACTGTTTC   4378
                    ************************************************************

w.t._745046         TTGGAATTTGCGAGAAATGCTTGCACATGCAGAAGAAACACGCAAATTAATGCCTGTCTG   4436
Alpha_737204        TTGGAATTTGCGAGAAATGCTTGCACATGCAGAAGAAACACGCAAATTAATGCCTGTCTG   4440
Delta_2183060       TTGGAATTTGCGAGAAATGCTTGCACATGCAGAAGAAACACGCAAATTAATGCCTGTCTG   4429
Omicron_7869197     TTGGAATTTGCGAGAAATGCTTGCACATGCAGAAGAAACACGCAAATTAATGCCTGTCTG   4438
                    ************************************************************

w.t._745046         TGTGGAAACTAAAGCCATAGTTTCAACTATACAGCGTAAATATAAGGGTATTAAAATACA   4496
Alpha_737204        TGTGGAAACTAAAGCCATAGTTTCAACTATACAGCGTAAATATAAGGGTATTAAAATACA   4500
Delta_2183060       TGTGGAAACTAAAGCCATAGTTTCAACTATACAGCGTAAATATAAGGGTATTAAAATACA   4489
Omicron_7869197     TGTGGAAACTAAAGCCATAGTTTCAACTATACAGCGTAAATATAAGGGTATTAAAATACA   4498
                    ************************************************************

w.t._745046         AGAGGGTGTAGTTGATTATGGTGCTAGATTTTACTTTTACACCAGTAAAACAACTGTAGC   4556
Alpha_737204        AGAGGGTGTGGTTGATTATGGTGCTAGATTTTACTTTTACACCAGTAAAACAACTGTAGC   4560
Delta_2183060       AGAGGGTGTGGTTGATTATGGTGCTAGATTTTACTTTTACACCAGTAAAACAACTGTAGC   4549
Omicron_7869197     AGAGGGTGTGGTTGATTATGGTGCTAGATTTTACTTTTACACCAGTAAAACAACTGTAGC   4558
                    ********* **************************************************

w.t._745046         GTCACTTATCAACACACTTAACGATCTAAATGAAACTCTTGTTACAATGCCACTTGGCTA   4616
Alpha_737204        GTCACTTATCAACACACTTAACGATCTAAATGAAACTCTTGTTACAATGCCACTTGGCTA   4620
Delta_2183060       GTCACTTATCAACACACTTAACGATCTAAATGAAACTCTTGTTACAATGCCACTTGGCTA   4609
Omicron_7869197     GTCACTTATCAACACACTTAACGATCTAAATGAAACTCTTGTTACAATGCCACTTGGCTA   4618
                    ************************************************************

w.t._745046         TGTAACACATGGCTTAAATTTGGAAGAAGCTGCTCGGTATATGAGATCTCTCAAAGTGCC   4676
Alpha_737204        TGTAACACATGGCTTAAATTTGGAAGAAGCTGCTCGGTATATGAGATCTCTCAAAGTGCC   4680
Delta_2183060       TGTAACACATGGCTTAAATTTGGAAGAAGCTGCTCGGTATATGAGATCTCTCAAAGTGCC   4669
Omicron_7869197     TGTAACACATGGCTTAAATTTGGAAGAAGCTGCTCGGTATATGAGATCTCTCAAAGTGCC   4678
                    ************************************************************

w.t._745046         AGCTACAGTTTCTGTTTCCTCACCTGATGCTGTTACAGCGTATAATGGTTATCTTACCTC   4736
Alpha_737204        AGCTACAGTTTCTGTTTCCTCACCTGATGCTGTTACAGCGTATAATGGTTATCTTACCTC   4740
Delta_2183060       AGCTACAGTTTCTGTTTCCTCACCTGATGCTGTTACAGCGTATAATGGTTATCTTACCTC   4729
Omicron_7869197     AGCTACAGTTTCTGTTTCCTCACCTGATGCTGTTACAGCGTATAATGGTTATCTTACCTC   4738
                    ************************************************************

w.t._745046         TTCCTCTAAAACACCTGAAGAACATTTTATTGAAACCATCTCACTTGCTGGTTCCTATAA   4796
Alpha_737204        TTCCTCTAAAACACCTGAAGAACATTTTATTGAAACCATCTCACTTGCTGGTTCCTATAA   4800
Delta_2183060       TTCCTCTAAAACACCTGAAGAACATTTTATTGAAACCATCTCACTTGCTGGTTCCTATAA   4789
Omicron_7869197     TTCCTCTAAAACACCTGAAGAACATTTTATTGAAACCATCTCACTTGCTGGTTCCTATAA   4798
                    ************************************************************
```

```
w.t._745046         AGATTGGTCCTATTCTGGACAATCTACACAACTAGGTATAGAATTTCTTAAGAGAGGTGA   4856
Alpha_737204        AGATTGGTCCTATTCTGGACAATCTACACAACTAGGTATAGAATTTCTTAAGAGAGGTGA   4860
Delta_2183060       AGATTGGTCCTATTCTGGACAATCTACACAACTAGGTATAGAATTTCTTAAGAGAGGTGA   4849
Omicron_7869197     AGATTGGTCCTATTCTGGACAATCTACACAACTAGGTATAGAATTTCTTAAGAGAGGTGA   4858
                    ************************************************************

w.t._745046         TAAAAGTGTATATTACACTAGTAATCCTACCACATTCCACCTAGATGGTGAAGTTATCAC   4916
Alpha_737204        TAAAAGTGTATATTACACTAGTAATCCTACCACATTCCACCTAGATGGTGAAGTTATCAC   4920
Delta_2183060       TAAAAGTGTATATTACACTAGTAATCCTACCACATTCCACCTAGATGGTGAAGTTATCAC   4909
Omicron_7869197     TAAAAGTGTATATTACACTAGTAATCCTACCACATTCCACCTAGATGGTGAAGTTATCAC   4918
                    ************************************************************

w.t._745046         CTTTGACAATCTTAAGACACCTCTTTCTTTGAGAGAAGTGAGGACTATTAAGGTGTTTAC   4976
Alpha_737204        CTTTGACAATCTTAAGACACCTCTTTCTTTGAGAGAAGTGAGGACTATTAAGGTGTTTAC   4980
Delta_2183060       CTTTGACAATCTTAAGACACCTCTTTCTTTGAGAGAAGTGAGGACTATTAAGGTGTTTAC   4969
Omicron_7869197     CTTTGACAATCTTAAGACACCTCTTTCTTTGAGAGAAGTGAGGACTATTAAGGTGTTTAC   4978
                    ************************************************************

w.t._745046         AACAGTAGACAACATTAACCTCCACACGCAAGTTGTGGACATGTCAATGACATATGGACA   5036
Alpha_737204        AACAGTAGACAACATTAACCTCCACACGCAAGTTGTGGACATGTCAATGACATATGGACA   5040
Delta_2183060       AACAGTAGACAACATTAACCTCCACACGCAAGTTGTGGACATGTCAATGACATATGGACA   5029
Omicron_7869197     AACAGTAGACAACATTAACCTCCACACGCAAGTTGTGGACATGTCAATGACATATGGACA   5038
                    ************************************************************

w.t._745046         ACAGTTTGGTCCAACTTATTTGGATGGAGCTGATGTTACTAAAATAAAACCTCATAATTC   5096
Alpha_737204        ACAGTTTGGTCCAACTTATTTGGATGGAGCTGATGTTACTAAAATAAAACCTCATAATTC   5100
Delta_2183060       ACAGTTTGGTCCAACTTATTTGGATGGAGCTGATGTTACTAAAATAAAACCTCATAATTC   5089
Omicron_7869197     ACAGTTTGGTCCAACTTATTTGGATGGAGCTGATGTTACTAAAATAAAACCTCATAATTC   5098
                    ************************************************************

w.t._745046         ACATGAAGGTAAAACATTTTATGTTTTACCTAATGATGACACTCTACGTGTTGAGGCTTT   5156
Alpha_737204        ACATGAAGGTAAAACATTTTATGTTTTACCTAATGATGACACTCTACGTGTTGAGGCTTT   5160
Delta_2183060       ACATGAAGGTAAAACATTTTATGTTTTACCTAATGATGACACTCTACGTGTTGAGGCTTT   5149
Omicron_7869197     ACATGAAGGTAAAACATTTTATGTTTTACCTAATGATGACACTCTACGTGTTGAGGCTTT   5158
                    ************************************************************

w.t._745046         TGAGTACTACCACACAACTGATCCTAGTTTTCTGGGTAGGTACATGTCAGCATTAAATCA   5216
Alpha_737204        TGAGTACTACCACACAACTGATCCTAGTTTTCTGGGTAGGTACATGTCAGCATTAAATCA   5220
Delta_2183060       TGAGTACTACCACACAACTGATCTTAGTTTTCTGGGTAGGTACATGTCAGCATTAAATCA   5209
Omicron_7869197     TGAGTACTACCACACAACTGATCCTAGTTTTCTGGGTAGGTACATGTCAGCATTAAATCA   5218
                    *********************** ************************************

w.t._745046         CACTAAAAAGTGGAAATACCCACAAGTTAATGGTTTAACCTCTATTAAATGGGCAGATAA   5276
Alpha_737204        CACTAAAAAGTGGAAATACCCACAAGTTAATGGTTTAACCTCTATTAAATGGGCAGATAA   5280
Delta_2183060       CACTAAAAAGTGGAAATACCCACAAGTTAATGGTTTAACCTCTATTAAATGGGCAGATAA   5269
Omicron_7869197     CACTAAAAAGTGGAAATACCCACAAGTTAATGGTTTAACCTCTATTAAATGGGCAGATAA   5278
                    ************************************************************

w.t._745046         CAACTGTTATCTTGCCACTGCATTGTTAACACTCCAACAAATAGAGTTGAAGTTTAATCC   5336
Alpha_737204        CAACTGTTATCTTGCCACTGCATTGTTAACACTCCAACAAATAGAGTTGAAGTTTAATCC   5340
Delta_2183060       CAACTGTTATCTTGCCACTGCATTGTTAACACTCCAACAAATAGAGTTGAAGTTTAATCC   5329
Omicron_7869197     CAACTGTTATCTTGCCACTGCATTGTTAACACTCCAACAAATAGAGTTGAAGTTTAATCC   5338
                    ************************************************************

w.t._745046         ACCTGCTCTACAAGATGCTTATTACAGAGCAAGGGCTGGTGAAGCTGCTAACTTTTGTGC   5396
Alpha_737204        ACCTGCTCTACAAGATGCTTATTACAGAGCAAGGGCTGGTGAAGCTGATAACTTTTGTGC   5400
Delta_2183060       ACCTGCTCTACAAGATGCTTATTACAGAGCAAGGGCTGGTGAAGCTGCTAACTTTTGTGC   5389
Omicron_7869197     ACCTGCTCTACAAGATGCTTATTACAGAGCAAGGGCTGGTGAAGCGGCTAACTTTTGTGC   5398
                    ********************************************* * ************
```

```
w.t._745046        ACTTATCTTAGCCTACTGTAATAAGACAGTAGGTGAGTTAGGTGATGTTAGAGAAACAAT   5456
Alpha_737204       ACTTATCTTAGCCTACTGTAATAAGACAGTAGGTGAGTTAGGTGATGTTAGAGAAACAAT   5460
Delta_2183060      ACTTATCTTAGCCTACTGTAATAAGACAGTAGGTGAGTTAGGTGATGTTAGAGAAACAAT   5449
Omicron_7869197    ACTTATCTTAGCCTACTGTAATAAGACAGTAGGTGAGTTAGGTGATGTTAGAGAAACAAT   5458
                   ************************************************************

w.t._745046        GAGTTACTTGTTTCAACATGCCAATTTAGATTCTTGCAAAAGAGTCTTGAACGTGGTGTG   5516
Alpha_737204       GAGTTACTTGTTTCAACATGCCAATTTAGATTCTTGCAAAAGAGTCTTGAACGTGGTGTG   5520
Delta_2183060      GAGTTACTTGTTTCAACATGCCAATTTAGATTCTTGCAAAAGAGTCTTGAACGTGGTGTG   5509
Omicron_7869197    GAGTTACTTGTTTCAACATGCCAATTTAGATTCTTGCAAAAGAGTCTTGAACGTGGTGTG   5518
                   ************************************************************

w.t._745046        TAAAACTTGTGGACAACAGCAGACAACCCTTAAGGGTGTAGAAGCTGTTATGTACATGGG   5576
Alpha_737204       TAAAACTTGTGGACAACAGCAGACAACCCTTAAGGGTGTAGAAGCTGTTATGTACATGGG   5580
Delta_2183060      TAAAACTTGTGGACAACAGCAGACAACCCTTAAGGGTGTAGAAGCTGTTATGTACATGGG   5569
Omicron_7869197    TAAAACTTGTGGACAACAGCAGACAACCCTTAAGGGTGTAGAAGCTGTTATGTACATGGG   5578
                   ************************************************************

w.t._745046        CACACTTTCTTATGAACAATTTAAGAAAGGTGTTCAGATACCTTGTACGTGTGGTAAACA   5636
Alpha_737204       CACACTTTCTTATGAACAATTTAAGAAAGGTGTTCAGATACCTTGTACGTGTGGTAAACA   5640
Delta_2183060      CACACTTTCTTATGAACAATTTAAGAAAGGTGTTCAGATACCTTGTACGTGTGGTAAACA   5629
Omicron_7869197    CACACTTTCTTATGAACAATTTAAGAAAGGTGTTCAGATACCTTGTACGTGTGGTAAACA   5638
                   ************************************************************

w.t._745046        AGCTACAAAATATCTAGTACAACAGGAGTCACCTTTTGTTATGATGTCAGCACCACCTGC   5696
Alpha_737204       AGCTACAAAATATCTAGTACAACAGGAGTCACCTTTTGTTATGATGTCAGCACCACCTGC   5700
Delta_2183060      AGCTACAAAATATCTAGTACAACAGGAGTCACCTTTTGTTATGATGTCAGCACCACCTGC   5689
Omicron_7869197    AGCTACAAAATATCTAGTACAACAGGAGTCACCTTTTGTTATGATGTCAGCACCACCTGC   5698
                   ************************************************************

w.t._745046        TCAGTATGAACTTAAGCATGGTACATTTACTTGTGCTAGTGAGTACACTGGTAATTACCA   5756
Alpha_737204       TCAGTATGAACTTAAGCATGGTACATTTACTTGTGCTAGTGAGTACACTGGTAATTACCA   5760
Delta_2183060      TCAGTATGAACTTAAGCATGGTACATTTACTTGTGCTAGTGAGTACACTGGTAATTACCA   5749
Omicron_7869197    TCAGTATGAACTTAAGCATGGTACATTTACTTGTGCTAGTGAGTACACTGGTAATTACCA   5758
                   ************************************************************

w.t._745046        GTGTGGTCACTATAAACATATAACCTCTAAAGAAACTTTGTATTGCATAGACGGTGCTTT   5816
Alpha_737204       GTGTGGTCACTATAAACATATAACCTCTAAAGAAACTTTGTATTGCATAGACGGTGCTTT   5820
Delta_2183060      GTGTGGTCACTATAAACATATAACCTCTAAAGAAACTTTGTATTGCATAGACGGTGCTTT   5809
Omicron_7869197    GTGTGGTCACTATAAACATATAACCTCTAAAGAAACTTTGTATTGCATAGACGGTGCTTT   5818
                   ************************************************************

w.t._745046        ACTTACAAAGTCCTCAGAATACAAAGGTCCTATTACGGATGTTTTCTACAAAGAAAACAG   5876
Alpha_737204       ACTTACAAAGTCCTCAGAATACAAAGGTCCTATTACGGATGTTTTCTACAAAGAAAACAG   5880
Delta_2183060      ACTTACAAAGTCCTCAGAATACAAAGGTCCTATTACGGATGTTTTCTACAAAGAAAACAG   5869
Omicron_7869197    ACTTACAAAGTCCTCAGAATACAAAGGTCCTATTACGGATGTTTTCTACAAAGAAAACAG   5878
                   ************************************************************

w.t._745046        TTACACAACAACCATAAAACCAGTTACTTATAAATTGGATGGTGTTGTTTGTACAGAAAT   5936
Alpha_737204       TTACACAACAACCATAAAACCAGTTACTTATAAATTGGATGGTGTTGTTTGTACAGAAAT   5940
Delta_2183060      TTACACAACAACCATAAAACCAGTTACTTATAAATTGGATGGTGTTGTTTGTACAGAAAT   5929
Omicron_7869197    TTACACAACAACCATAAAACCAGTTACTTATAAATTGGATGGTGTTGTTTGTACAGAAAT   5938
                   ************************************************************

w.t._745046        TGACCCTAAGTTGGACAATTATTATAAGAAAGACAATTCTTATTTCACAGAGCAACCAAT   5996
Alpha_737204       TGACCCTAAGTTGGACAATTATTATAAGAAAGACAATTCTTATTTTACAGAGCAACCAAT   6000
Delta_2183060      TGACCCTAAGTTGGACAATTATTATAAGAAAGACAATTCTTATTTCACAGAGCAACCAAT   5989
Omicron_7869197    TGACCCTAAGTTGGACAATTATTATAAGAAAGACAATTCTTATTTCACAGAGCAACCAAT   5998
                   ********************************************* **************
```

```
w.t._745046         TGATCTTGTACCAAACCAACCATATCCAAACGCAAGCCTCGATAATTTTAAGTTTGTATG   6056
Alpha_737204        TGATCTTGTACCAAACCAACCATATCCAAACGCAAGCCTCGATAATTTTAAGTTTGTATG   6060
Delta_2183060       TGATCTTGTACCAAACCAACCATATCCAAACGCAAGCCTCGATAATTTTAAGTTTGTATG   6049
Omicron_7869197     TGATCTTGTACCAAACCAACCATATCCAAACGCAAGCCTCGATAATTTTAAGTTTGTATG   6058
                    ************************************************************

w.t._745046         TGATAATATCAAATTTGCTGATGATTTAAACCAGTTAACTGGTTATAAGAAACCTGCCTC   6116
Alpha_737204        TGATAATATCAAATTTGCTGATGATTTAAACCAGTTAACTGGTTATAAGAAACCTGCCTC   6120
Delta_2183060       TGATAATATCAAATTTGCTGATGATTTAAACCAGTTAACTGGTTATAAGAAACCTGCCTC   6109
Omicron_7869197     TGATAATATCAAATTTGCTGATGATTTAAACCAGTTAACTGGTTATAAGAAACCTGCCTC   6118
                    ************************************************************

w.t._745046         AAGAGAGCTTAAAGTTACATTTTTCCCTGACTTAAATGGTGATGTGGTGGCTATTGATTA   6176
Alpha_737204        AAGAGAGCTTAAAGTTACATTTTTCCCTGACTTAAATGGTGATGTGGTGGCTATTGATTA   6180
Delta_2183060       AAGAGAGCTTAAAGTTACATTTTTCCCTGACTTAAATGGTGATGTGGTGGCTATTGATTA   6169
Omicron_7869197     AAGAGAGCTTAAAGTTACATTTTTCCCTGACTTAAATGGTGATGTGGTGGCTATTGATTA   6178
                    ************************************************************

w.t._745046         TAAACACTACACACCCTCTTTTAAGAAAGGAGCTAAATTGTTACATAAACCTATTGTTTG   6236
Alpha_737204        TAAACACTACACACCCTCTTTTAAGAAAGGAGCTAAATTGTTACATAAACCTATTGTTTG   6240
Delta_2183060       TAAACACTACACACCCTCTTTTAAGAAAGGAGCTAAATTGTTACATAAACCTATTGTTTG   6229
Omicron_7869197     TAAACACTACACACCCTCTTTTAAGAAAGGAGCTAAATTGTTACATAAACCTATTGTTTG   6238
                    ************************************************************

w.t._745046         GCATGTTAACAATGCAACTAATAAAGCCACGTATAAACCAAATACCTGGTGTATACGTTG   6296
Alpha_737204        GCATGTTAACAATGCAACTAATAAAGCCACGTATAAACCAAATACCTGGTGTATACGTTG   6300
Delta_2183060       GCATGTTAACAATGCAACTAATAAAGCCACGTATAAACCAAATACCTGGTGTATACGTTG   6289
Omicron_7869197     GCATGTTAACAATGCAACTAATAAAGCCACGTATAAACCAAATACCTGGTGTATACGTTG   6298
                    ************************************************************

w.t._745046         TCTTTGGAGCACAAAACCAGTTGAAACATCAAATTCGTTTGATGTACTGAAGTCAGAGGA   6356
Alpha_737204        TCTTTGGAGCACAAAACCAGTTGAAACATCAAATTCGTTTGATGTACTGAAGTCAGAGGA   6360
Delta_2183060       TCTTTGGAGCACAAAACCAGTTGAAACATCAAATTCGTTTGATGTACTGAAGTCAGAGGA   6349
Omicron_7869197     TCTTTGGAGCACAAAACCAGTTGAAACATCAAATTCGTTTGATGTACTGAAGTCAGAGGA   6358
                    ************************************************************

w.t._745046         CGCGCAGGGAATGGATAATCTTGCCTGCGAAGATCTAAAACCAGTCTCTGAAGAAGTAGT   6416
Alpha_737204        CGCGCAGGGAATGGATAATCTTGCCTGCGAAGATCTAAAACCAGTCTCTGAAGAAGTAGT   6420
Delta_2183060       CGCGCAGGGAATGGATAATCTTGCCTGCGAAGATCTAAAACCAGTCTCTGAAGAAGTAGT   6409
Omicron_7869197     CGCGCAGGGAATGGATAATCTTGCCTGCGAAGATCTAAAACCAGTCTCTGAAGAAGTAGT   6418
                    ************************************************************

w.t._745046         GGAAAATCCTACCATACAGAAAGACGTTCTTGAGTGTAATGTGAAAACTACCGAAGTTGT   6476
Alpha_737204        GGAAAATCCTACCATACAGAAAGACGTTCTTGAGTGTAATGTGAAAACTACCGAAGTTGT   6480
Delta_2183060       GGAAAATCCTACCATACAGAAAGACGTTCTTGAGTGTAATGTGAAAACTACCGAAGTTGT   6469
Omicron_7869197     GGAAAATCCTACCATACAGAAAGACGTTCTTGAGTGTAATGTGAAAACTACCGAAGTTGT   6478
                    ************************************************************

w.t._745046         AGGAGACATTATACTTAAACCAGCAAATAATAGTTTAAAAATTACAGAAGAGGTTGGCCA   6536
Alpha_737204        AGGAGACATTATACTTAAACCAGCAAATAATAGTTTAAAAATTACAGAAGAGGTTGGCCA   6540
Delta_2183060       AGGAGACATTATACTTAAACCAGCAAATAATAGTTTAAAAATTACAGAAGAGGTTGGCTA   6529
Omicron_7869197     AGGAGACATTATACTTAAACCAGCAAATAATA---TAAAAATTACAGAAGAGGTTGGCCA   6535
                    ********************************   *********************** *

w.t._745046         CACAGATCTAATGGCTGCTTATGTAGACAATTCTAGTCTTACTATTAAGAAACCTAATGA   6596
Alpha_737204        CACAGATCTAATGGCTGCTTATGTAGACAATTCTAGTCTTACTATTAAGAAACCTAATGA   6600
Delta_2183060       CACAGATCTAATGGCTGCTTATGTAGACAATTCTAGTCTTACTATTAAGAAACCTAATGA   6589
Omicron_7869197     CACAGATCTAATGGCTGCTTATGTAGACAATTCTAGTCTTACTATTAAGAAACCTAATGA   6595
                    ************************************************************
```

```
w.t._745046         ATTATCTAGAGTATTAGGTTTGAAAACCCTTGCTACTCATGGTTTAGCTGCTGTTAATAG   6656
Alpha_737204        ATTATCTAGAGTATTAGGTTTGAAAACCCTTGCTACTCATGGTTTAGCTGCTGTTAATAG   6660
Delta_2183060       ATTATCTAGAGTATTAGGTTTGAAAACCCTTGCTACTCATGGTTTAGCTGCTGTTAATAG   6649
Omicron_7869197     ATTATCTAGAGTATTAGGTTTGAAAACCCTTGCTACTCATGGTTTAGCTGCTGTTAATAG   6655
                    ************************************************************

w.t._745046         TGTCCCTTGGGATACTATAGCTAATTATGCTAAGCCTTTTCTTAACAAAGTTGTTAGTAC   6716
Alpha_737204        TGTCCCTTGGGATACTATAGCTAATTATGCTAAGCCTTTTCTTAACAAAGTTGTTAGTAC   6720
Delta_2183060       TGTCCCTTGGGATACTATAGCTAATTATGCTAAGCCTTTTCTTAACAAAGTTGTTAGTAC   6709
Omicron_7869197     TGTCCCTTGGGATACTATAGCTAATTATGCTAAGCCTTTTCTTAACAAAGTTGTTAGTAC   6715
                    ************************************************************

w.t._745046         AACTACTAACATAGTTACACGGTGTTTAAACCGTGTTTGTACTAATTATATGCCTTATTT   6776
Alpha_737204        AACTACTAACATAGTTACACGGTGTTTAAACCGTGTTTGTACTAATTATATGCCTTATTT   6780
Delta_2183060       AACTACTAACATAGTTACACGGTGTTTAAACCGTGTTTGTACTAATTATATGCCTTATTT   6769
Omicron_7869197     AACTACTAACATAGTTACACGGTGTTTAAACCGTGTTTGTACTAATTATATGCCTTATTT   6775
                    ************************************************************

w.t._745046         CTTTACTTTATTGCTACAATTGTGTACTTTTACTAGAAGTACAAATTCTAGAATTAAAGC   6836
Alpha_737204        CTTTACTTTATTGCTACAATTGTGTACTTTTACTAGAAGTACAAATTCTAGAATTAAAGC   6840
Delta_2183060       CTTTACTTTATTGCTACAATTGTGTACTTTTACTAGAAGTACAAATTCTAGAATTAAAGC   6829
Omicron_7869197     CTTTACTTTATTGCTACAATTGTGTACTTTTACTAGAAGTACAAATTCTAGAATTAAAGC   6835
                    ************************************************************

w.t._745046         ATCTATGCCGACTACTATAGCAAAGAATACTGTTAAGAGTGTCGGTAAATTTTGTCTAGA   6896
Alpha_737204        ATCTATGCCGACTACTATAGCAAAGAATACTGTTAAGAGTGTCGGTAAATTTTGTCTAGA   6900
Delta_2183060       ATCTATGCCGACTACTATAGCAAAGAATACTGTTAAGAGTGTCGGTAAATTTTGTCTAGA   6889
Omicron_7869197     ATCTATGCCGACTACTATAGCAAAGAATACTGTTAAGAGTGTCGGTAAATTTTGTCTAGA   6895
                    ************************************************************

w.t._745046         GGCCTCATTTAATTATTTGAAGTCACCTAATTTTTCTAAACTGATAAATATTATAATTTG   6956
Alpha_737204        GGCCTCATTTAATTATTTGAAGTCACCTAATTTTTCTAAACTGATAAATATTACAATTTG   6960
Delta_2183060       GGCCTCATTTAATTATTTGAAGTCACCTAATTTTTCTAAACTGATAAATATTATAATTTG   6949
Omicron_7869197     GGCCTCATTTAATTATTTGAAGTCACCTAATTTTTCTAAACTGATAAATATTATAATTTG   6955
                    ***************************************************** ******

w.t._745046         GTTTTTACTATTAAGTGTTTGCCTAGGTTCTTTAATCTACTCAACCGCTGCTTTAGGTGT   7016
Alpha_737204        GTTTTTACTATTAAGTGTTTGCCTAGGTTCTTTAATCTACTCAACCGCTGCTTTAGGTGT   7020
Delta_2183060       GTTTTTACTATTAAGTGTTTGCCTAGGTTCTTTAATCTACTCAACCGCTGCTTTAGGTGT   7009
Omicron_7869197     GTTTTTACTATTAAGTGTTTGCCTAGGTTCTTTAATCTACTCAACCGCTGCTTTAGGTGT   7015
                    ************************************************************

w.t._745046         TTTAATGTCTAATTTAGGCATGCCCTCTTACTGTACTGGTTACAGAGAAGGCTATTTGAA   7076
Alpha_737204        TTTAATGTCTAATTTAGGCATGCCCTCTTACTGTACTGGTTACAGAGAAGGCTATTTGAA   7080
Delta_2183060       TTTAATGTCTAATTTAGGCATGCCCTCTTACTGTACTGGTTACAGAGAAGGCTATTTGAA   7069
Omicron_7869197     TTTAATGTCTAATTTAGGCATGCCCTCTTACTGTACTGGTTACAGAGAAGGCTATTTGAA   7075
                    ************************************************************

w.t._745046         CTCTACTAATGTCACTATTGCAACCTACTGTACTGGTTCTATACCTTGTAGTGTTTGTCT   7136
Alpha_737204        CTCTACTAATGTCACTATTGCAACCTACTGTACTGGTTCTATACCTTGTAGTGTTTGTCT   7140
Delta_2183060       CTCTACTAATGTCACTATTGCAACCTACTGTACTGGTTCTATACCTTGTAGTGTTTGTCT   7129
Omicron_7869197     CTCTACTAATGTCACTATTGCAACCTACTGTACTGGTTCTATACCTTGTAGTGTTTGTCT   7135
                    ************************************************************

w.t._745046         TAGTGGTTTAGATTCTTTAGACACCTATCCCTCTTTAGAAACTATACAAATTACCATTTC   7196
Alpha_737204        TAGTGGTTTAGATTCTTTAGACACCTATCCCTCTTTAGAAACTATACAAATTACCATTTC   7200
Delta_2183060       TAGTGGTTTAGATTCTTTAGACACCTATCCCTCTTTAGAAACTATACAAATTACCATTTC   7189
Omicron_7869197     TAGTGGTTTAGATTCTTTAGACACCTATCCCTCTTTAGAAACTATACAAATTACCATTTC   7195
                    ************************************************************
```

```
w.t._745046        ATCTTTTAAATGGGATTTAACTGCTTTTGGCTTAGTTGCAGAGTGGTTTTTGGCATATAT   7256
Alpha_737204       ATCTTTTAAATGGGATTTAACTGCTTTTGGCTTAGTTGCAGAGTGGTTTTTGGCATATAT   7260
Delta_2183060      ATCTTTTAAATGGGATTTAACTGCTTTTGGCTTAGTTGCAGAGTGGTTTTTGGCATATAT   7249
Omicron_7869197    ATCTTTTAAATGGGATTTAACTGCTTTTGGCTTAGTTGCAGAGTGGTTTTTGGCATATAT   7255
                   ************************************************************

w.t._745046        TCTTTTCACTAGGTTTTTCTATGTACTTGGATTGGCTGCAATCATGCAATTGTTTTTCAG   7316
Alpha_737204       TCTTTTCACTAGGTTTTTCTATGTACTTGGATTGGCTGCAATCATGCAATTGTTTTTCAG   7320
Delta_2183060      TCTTTTCACTAGGTTTTTCTATGTACTTGGATTGGCTGCAATCATGCAATTGTTTTTCAG   7309
Omicron_7869197    TCTTTTCACTAGGTTTTTCTATGTACTTGGATTGGCTGCAATCATGCAATTGTTTTTCAG   7315
                   ************************************************************

w.t._745046        CTATTTTGCAGTACATTTTATTAGTAATTCTTGGCTTATGTGGTTAATAATTAATCTTGT   7376
Alpha_737204       CTATTTTGCAGTACATTTTATTAGTAATTCTTGGCTTATGTGGTTAATAATTAATCTTGT   7380
Delta_2183060      CTATTTTGCAGTACATTTTATTAGTAATTCTTGGCTTATGTGGTTAATAATTAATCTTGT   7369
Omicron_7869197    CTATTTTGCAGTACATTTTATTAGTAATTCTTGGCTTATGTGGTTAATAATTAATCTTGT   7375
                   ************************************************************

w.t._745046        ACAAATGGCCCCGATTTCAGCTATGGTTAGAATGTACATCCTCTTTGCATCATTTTATTA   7436
Alpha_737204       ACAAATGGCCCCGATTTCAGCTATGGTTAGAATGTACATCCTCTTTGCATCATTTTATTA   7440
Delta_2183060      ACAAATGGCCCCGATTTCAGCTATGGTTAGAATGTACATCCTCTTTGCATCATTTTATTA   7429
Omicron_7869197    ACAAATGGCCCCGATTTCAGCTATGGTTAGAATGTACATCCTCTTTGCATCATTTTATTA   7435
                   ************************************************************

w.t._745046        TGTATGGAAAAGTTATGTGCATGTTGTAGACGGTTGTAATTCATCAACTTGTATGATGTG   7496
Alpha_737204       TGTATGGAAAAGTTATGTGCATGTTGTAGACGGTTGTAATTCATCAACTTGTATGATGTG   7500
Delta_2183060      TGTATGGAAAAGTTATGTGCATGTTGTAGACGGTTGTAATTCATCAACTTGTATGATGTG   7489
Omicron_7869197    TGTATGGAAAAGTTATGTGCATGTTGTAGACGGTTGTAATTCATCAACTTGTATGATGTG   7495
                   ************************************************************

w.t._745046        TTACAAACGTAATAGAGCAACAAGAGTCGAATGTACAACTATTGTTAATGGTGTTAGAAG   7556
Alpha_737204       TTACAAACGTAATAGAGCAACAAGAGTCGAATGTACAACTATTGTTAATGGTGTTAGAAG   7560
Delta_2183060      TTACAAACGTAATAGAGCAACAAGAGTCGAATGTACAACTATTGTTAATGGTGTTAGAAG   7549
Omicron_7869197    TTACAAACGTAATAGAGCAACAAGAGTCGAATGTACAACTATTGTTAATGGTGTTAGAAG   7555
                   ************************************************************

w.t._745046        GTCCTTTTATGTCTATGCTAATGGAGGTAAAGGCTTTTGCAAACTACACAATTGGAATTG   7616
Alpha_737204       GTCCTTTTATGTCTATGCTAATGGAGGTAAAGGCTTTTGCAAACTACACAATTGGAATTG   7620
Delta_2183060      GTCCTTTTATGTCTATGCTAATGGAGGTAAAGGCTTTTGCAAACTACACAATTGGAATTG   7609
Omicron_7869197    GTCCTTTTATGTCTATGCTAATGGAGGTAAAGGCTTTTGCAAACTACACAATTGGAATTG   7615
                   ************************************************************

w.t._745046        TGTTAATTGTGATACATTCTGTGCTGGTAGTACATTTATTAGTGATGAAGTTGCGAGAGA   7676
Alpha_737204       TGTTAATTGTGATACATTCTGTGCTGGTAGTACATTTATTAGTGATGAAGTTGCGAGAGA   7680
Delta_2183060      TGTTAATTGTGATACATTCTGTGCTGGTAGTACATTTATTAGTGATGAAGTTGCGAGAGA   7669
Omicron_7869197    TGTTAATTGTGATACATTCTGTGCTGGTAGTACATTTATTAGTGATGAAGTTGCGAGAGA   7675
                   ************************************************************

w.t._745046        CTTGTCACTACAGTTTAAAAGACCAATAAATCCTACTGACCAGTCCTCTTACATCGTTGA   7736
Alpha_737204       CTTGTCACTACAGTTTAAAAGACCAATAAATCCTACTGACCAGTCCTCTTACATCGTTGA   7740
Delta_2183060      CTTGTCACTACAGTTTAAAAGACCAATAAATCCTACTGACCAGTCCTCTTACATCGTTGA   7729
Omicron_7869197    CTTGTCACTACAGTTTAAAAGACCAATAAATCCTACTGACCAGTCCTCTTACATCGTTGA   7735
                   ************************************************************

w.t._745046        TAGTGTTACAGTGAAGAATGGTTCCATCCATCTTTACTTTGATAAAGCTGGTCAAAAGAC   7796
Alpha_737204       TAGTGTTACAGTGAAGAATGGTTCCATCCATCTTTACTTTGATAAAGCTGGTCAAAAGAC   7800
Delta_2183060      TAGTGTTACAGTGAAGAATGGTTCCATCCATCTTTACTTTGATAAAGCTGGTCAAAAGAC   7789
Omicron_7869197    TAGTGTTACAGTGAAGAATGGTTCCATCCATCTTTACTTTGATAAAGCTGGTCAAAAGAC   7795
                   ************************************************************
```

```
w.t._745046        TTATGAAAGACATTCTCTCTCTCATTTTGTTAACTTAGACAACCTGAGAGCTAATAACAC   7856
Alpha_737204       TTATGAAAGACATTCTCTCTCTCATTTTGTTAACTTAGACAACCTGAGAGCTAATAACAC   7860
Delta_2183060      TTATGAAAGACATTCTCTCTCTCATTTTGTTAACTTAGACAACCTGAGAGCTAATAACAC   7849
Omicron_7869197    TTATGAAAGACATTCTCTCTCTCATTTTGTTAACTTAGACAACCTGAGAGCTAATAACAC   7855
                   ************************************************************

w.t._745046        TAAAGGTTCATTGCCTATTAATGTTATAGTTTTTGATGGTAAATCAAAATGTGAAGAATC   7916
Alpha_737204       TAAAGGTTCATTGCCTATTAATGTTATAGTTTTTGATGGTAAATCAAAATGTGAAGAATC   7920
Delta_2183060      TAAAGGTTCATTGCCTATTAATGTTATAGTTTTTGATGGTAAATCAAAATGTGAAGAATC   7909
Omicron_7869197    TAAAGGTTCATTGCCTATTAATGTTATAGTTTTTGATGGTAAATCAAAATGTGAAGAATC   7915
                   ************************************************************

w.t._745046        ATCTGCAAAATCAGCGTCTGTTTACTACAGTCAGCTTATGTGTCAACCTATACTGTTACT   7976
Alpha_737204       ATCTGCAAAATCAGCGTCTGTTTACTACAGTCAGCTTATGTGTCAACCTATACTGTTACT   7980
Delta_2183060      ATCTGCAAAATCAGCGTCTGTTTACTACAGTCAGCTTATGTGTCAACCTATACTGTTACT   7969
Omicron_7869197    ATCTGCAAAATCAGCGTCTGTTTACTACAGTCAGCTTATGTGTCAACCTATACTGTTACT   7975
                   ************************************************************

w.t._745046        AGATCAGGCATTAGTGTCTGATGTTGGTGATAGTGCGGAAGTTGCAGTTAAAATGTTTGA   8036
Alpha_737204       AGATCAGGCATTAGTGTCTGATGTTGGTGATAGTGCGGAAGTTGCAGTTAAAATGTTTGA   8040
Delta_2183060      AGATCAGGCATTAGTGTCTGATGTTGGTGATAGTGCGGAAGTTGCAGTTAAAATGTTTGA   8029
Omicron_7869197    AGATCAGGCATTAGTGTCTGATGTTGGTGATAGTGCGGAAGTTGCAGTTAAAATGTTTGA   8035
                   ************************************************************

w.t._745046        TGCTTACGTTAATACGTTTTCATCAACTTTTAACGTACCAATGGAAAAACTCAAAACACT   8096
Alpha_737204       TGCTTACGTTAATACGTTTTCATCAACTTTTAACGTACCAATGGAAAAACTCAAAACACT   8100
Delta_2183060      TGCTTACGTTAATACGTTTTCATCAACTTTTAACGTACCAATGGAAAAACTCAAAACACT   8089
Omicron_7869197    TGCTTACGTTAATACGTTTTCATCAACTTTTAACGTACCAATGGAAAAACTCAAAACACT   8095
                   ************************************************************

w.t._745046        AGTTGCAACTGCAGAAGCTGAACTTGCAAAGAATGTGTCCTTAGACAATGTCTTATCTAC   8156
Alpha_737204       AGTTGCAACTGCAGAAGCTGAACTTGCAAAGAATGTGTCCTTAGACAATGTCTTATCTAC   8160
Delta_2183060      AGTTGCAACTGCAGAAGCTGAACTTGCAAAGAATGTGTCCTTAGACAATGTCTTATCTAC   8149
Omicron_7869197    AGTTGCAACTGCAGAAGCTGAACTTGCAAAGAATGTGTCCTTAGACAATGTCTTATCTAC   8155
                   ************************************************************

w.t._745046        TTTTATTTCAGCAGCTCGGCAAGGGTTTGTTGATTCAGATGTAGAAACTAAAGATGTTGT   8216
Alpha_737204       TTTTATTTCAGCAGCTCGGCAAGGGTTTGTTGATTCAGATGTAGAAACTAAAGATGTTGT   8220
Delta_2183060      TTTTATTTCAGCAGCTCGGCAAGGGTTTGTTGATTCAGATGTAGAAACTAAAGATGTTGT   8209
Omicron_7869197    TTTTATTTCAGCAGCTCGGCAAGGGTTTGTTGATTCAGATGTAGAAACTAAAGATGTTGT   8215
                   ************************************************************

w.t._745046        TGAATGTCTTAAATTGTCACATCAATCTGACATAGAAGTTACTGGCGATAGTTGTAATAA   8276
Alpha_737204       TGAATGTCTTAAATTGTCACATCAATCTGACATAGAAGTTACTGGCGATAGTTGTAATAA   8280
Delta_2183060      TGAATGTCTTAAATTGTCACATCAATCTGACATAGAAGTTACTGGCGATAGTTGTAATAA   8269
Omicron_7869197    TGAATGTCTTAAATTGTCACATCAATCTGACATAGAAGTTACTGGCGATAGTTGTAATAA   8275
                   ************************************************************

w.t._745046        CTATATGCTCACCTATAACAAAGTTGAAAACATGACACCCCGTGACCTTGGTGCTTGTAT   8336
Alpha_737204       CTATATGCTCACCTATAACAAAGTTGAAAACATGACACCCCGTGACCTTGGTGCTTGTAT   8340
Delta_2183060      CTATATGCTCACCTATAACAAAGTTGAAAACATGACACCCCGTGACCTTGGTGCTTGTAT   8329
Omicron_7869197    CTATATGCTCACCTATAACAAAGTTGAAAACATGACACCCCGTGACCTTGGTGCTTGTAT   8335
                   ************************************************************

w.t._745046        TGACTGTAGTGCGCGTCATATTAATGCGCAGGTAGCAAAAAGTCACAACATTGCTTTGAT   8396
Alpha_737204       TGACTGTAGTGCGCGTCATATTAATGCGCAGGTAGCAAAAAGTCACAACATTGCTTTGAT   8400
Delta_2183060      TGACTGTAGTGCGCGTCATATTAATGCGCAGGTAGCAAAAAGTCACAACATTGCTTTGAT   8389
Omicron_7869197    TGACTGTAGTGCGCGTCATATTAATGCGCAGGTAGCAAAAAGTCACAACATTACTTTGAT   8395
                   **************************************************** *******
```

```
w.t._745046        ATGGAACGTTAAAGATTTCATGTCATTGTCTGAACAACTACGAAAACAAATACGTAGTGC   8456
Alpha_737204       ATGGAACGTTAAAGATTTCATGTCATTGTCTGAACAACTACGAAAACAAATACGTAGTGC   8460
Delta_2183060      ATGGAACGTTAAAGATTTCATGTCATTGTCTGAACAACTACGAAAACAAATACGTAGTGC   8449
Omicron_7869197    ATGGAACGTTAAAGATTTCATGTCATTGTCTGAACAACTACGAAAACAAATACGTAGTGC   8455
                   ************************************************************

w.t._745046        TGCTAAAAAGAATAACTTACCTTTTAAGTTGACATGTGCAACTACTAGACAAGTTGTTAA   8516
Alpha_737204       TGCTAAAAAGAATAACTTACCTTTTAAGTTGACATGTGCAACTACTAGACAAGTTGTTAA   8520
Delta_2183060      TGCTAAAAAGAATAACTTACCTTTTAAGTTGACATGTGCAACTACTAGACAAGTTGTTAA   8509
Omicron_7869197    TGCTAAAAAGAATAACTTACCTTTTAAGTTGACATGTGCAACTACTAGACAAGTTGTTAA   8515
                   ************************************************************

w.t._745046        TGTTGTAACAACAAAGATAGCACTTAAGGGTGGTAAAATTGTTAATAATTGGTTGAAGCA   8576
Alpha_737204       TGTTGTAACAACAAAGATAGCACTTAAGGGTGGTAAAATTGTTAATAATTGGTTGAAGCA   8580
Delta_2183060      TGTTGTAACAACAAAGATAGCACTTAAGGGTGGTAAAATTGTTAATAATTGGTTGAAGCA   8569
Omicron_7869197    TGTTGTAACAACAAAGATAGCACTTAAGGGTGGTAAAATTGTTAATAATTGGTTGAAGCA   8575
                   ************************************************************

w.t._745046        GTTAATTAAAGTTACACTTGTGTTCCTTTTTGTTGCTGCTATTTTCTATTTAATAACACC   8636
Alpha_737204       GTTAATTAAAGTTACACTTGTGCTCCTTTTTGTTGCTGCTATTTTCTATTTAATAACACC   8640
Delta_2183060      GTTAATTAAAGTTACACTTGTGTTCCTTTTTGTTGCTGCTATTTTCTATTTAATAACACC   8629
Omicron_7869197    GTTAATTAAAGTTACACTTGTGTTCCTTTTTGTTGCTGCTATTTTCTATTTAATAACACC   8635
                   ********************** *************************************

w.t._745046        TGTTCATGTCATGTCTAAACATACTGACTTTTCAAGTGAAATCATAGGATACAAGGCTAT   8696
Alpha_737204       TGTTCATGTCATGTCTAAACATACTGACTTTTCAAGTGAAATCATAGGATACAAGGCTAT   8700
Delta_2183060      TGTTCATGTCATGTCTAAACATACTGACTTTTCAAGTGAAATCATAGGATACAAGGCTAT   8689
Omicron_7869197    TGTTCATGTCATGTCTAAACATACTGACTTTTCAAGTGAAATCATAGGATACAAGGCTAT   8695
                   ************************************************************

w.t._745046        TGATGGTGGTGTCACTCGTGACATAGCATCTACAGATACTTGTTTTGCTAACAAACATGC   8756
Alpha_737204       TGATGGTGGTGTCACTCGTGACATAGCATCTACAGATACTTGTTTTGCTAACAAACATGC   8760
Delta_2183060      TGATGGTGGTGTCACTCGTGACATAGCATCTACAGATACTTGTTTTGCTAACAAACATGC   8749
Omicron_7869197    TGATGGTGGTGTCACTCGTGACATAGCATCTACAGATACTTGTTTTGCTAACAAACATGC   8755
                   ************************************************************

w.t._745046        TGATTTTGACACATGGTTTAGCCAGCGTGGTGGTAGTTATACTAATGACAAAGCTTGCCC   8816
Alpha_737204       TGATTTTGACACATGGTTTAGCCAGCGTGGTGGTAGTTATACTAATGACAAAGCTTGCCC   8820
Delta_2183060      TGATTTTGACACATGGTTTAGCCAGCGTGGTGGTAGTTATACTAATGACAAAGCTTGCCC   8809
Omicron_7869197    TGATTTTGACACATGGTTTAGCCAGCGTGGTGGTAGTTATACTAATGACAAAGCTTGCCC   8815
                   ************************************************************

w.t._745046        ATTGATTGCTGCAGTCATAACAAGAGAAGTGGGTTTTGTCGTGCCTGGTTTGCCTGGCAC   8876
Alpha_737204       ATTGATTGCTGCAGTCATAACAAGAGAAGTGGGTTTTGTCGTGCCTGGTTTGCCTGGCAC   8880
Delta_2183060      ATTGATTGCTGCAGTCATAACAAGAGAAGTGGGTTTTGTCGTGCCTGGTTTGCCTGGCAC   8869
Omicron_7869197    ATTGATTGCTGCAGTCATAACAAGAGAAGTGGGTTTTGTCGTGCCTGGTTTGCCTGGCAC   8875
                   ************************************************************

w.t._745046        GATATTACGCACAACTAATGGTGACTTTTTGCATTTCTTACCTAGAGTTTTTAGTGCAGT   8936
Alpha_737204       GATATTACGCACAACTAATGGTGACTTTTTGCATTTCTTACCTAGAGTTTTTAGTGCAGT   8940
Delta_2183060      GATATTACGCACAACTAATGGTGACTTTTTGCATTTCTTACCTAGAGTTTTTAGTGCAGT   8929
Omicron_7869197    GATATTACGCACAACTAATGGTGACTTTTTGCATTTCTTACCTAGAGTTTTTAGTGCAGT   8935
                   ************************************************************

w.t._745046        TGGTAACATCTGTTACACACCATCAAAACTTATAGAGTACACTGACTTTGCAACATCAGC   8996
Alpha_737204       TGGTAACATCTGTTACACACCATCAAAACTTATAGAGTACACTGACTTTGCAACATCAGC   9000
Delta_2183060      TGGTAACATCTGTTACACACCATCAAAACTTATAGAGTACACTGACTTTGCAACATCAGC   8989
Omicron_7869197    TGGTAACATCTGTTACACACCATCAAAACTTATAGAGTACACTGACTTTGCAACATCAGC   8995
                   ************************************************************
```

```
w.t._745046        TTGTGTTTTGGCTGCTGAATGTACAATTTTTAAAGATGCCTCTGGTAAGCCAGTACCATA   9056
Alpha_737204       TTGTGTTTTGGCTGCTGAATGTACAATTTTTAAAGATGCCTCTGGTAAGCCAGTACCATA   9060
Delta_2183060      TTGTGTTTTGGCTGCTGAATGTACAATTTTTAAAGATGCCTCTGGTAAGCCAGTACCATA   9049
Omicron_7869197    TTGTGTTTTGGCTGCTGAATGTACAATTTTTAAAGATGCCTCTGGTAAGCCAGTACCATA   9055
                   ************************************************************

w.t._745046        TTGTTATGATACCAATGTACTAGAAGGTTCTGTTGCTTATGAAAGTTTACGCCCTGACAC   9116
Alpha_737204       TTGTTATGATACCAATGTACTAGAAGGTTCTGTTGCTTATGAAAGTTTACGCCCTGACAC   9120
Delta_2183060      TTGTTATGATACCAATGTACTAGAAGGTTCTGTTGCTTATGAAAGTTTACGCCCTGACAC   9109
Omicron_7869197    TTGTTATGATACCAATGTACTAGAAGGTTCTGTTGCTTATGAAAGTTTACGCCCTGACAC   9115
                   ************************************************************

w.t._745046        ACGTTATGTGCTCATGGATGGCTCTATTATTCAATTTCCTAACACCTACCTTGAAGGTTC   9176
Alpha_737204       ACGTTATGTGCTCATGGATGGCTCTATTATTCAATTTCCTAACACCTACCTTGAAGGTTC   9180
Delta_2183060      ACGTTATGTGCTCATGGATGGCTCTATTATTCAATTTCCTAACACCTACCTTGAAGGTTC   9169
Omicron_7869197    ACGTTATGTGCTCATGGATGGCTCTATTATTCAATTTCCTAACACCTACCTTGAAGGTTC   9175
                   ************************************************************

w.t._745046        TGTTAGAGTGGTAACAACTTTTGATTCTGAGTACTGTAGGCACGGCACTTGTGAAAGATC   9236
Alpha_737204       TGTTAGAGTGGTAACAACTTTTGATTCTGAGTACTGTAGGCACGGCACTTGTGAAAGATC   9240
Delta_2183060      TGTTAGAGTGGTAACAACTTTTGATTCTGAGTACTGTAGGCACGGCACTTGTGAAAGATC   9229
Omicron_7869197    TGTTAGAGTGGTAACAACTTTTGATTCTGAGTACTGTAGGCACGGCACTTGTGAAAGATC   9235
                   ************************************************************

w.t._745046        AGAAGCTGGTGTTTGTGTATCTACTAGTGGTAGATGGGTACTTAACAATGATTATTACAG   9296
Alpha_737204       AGAAGCTGGTGTTTGTGTATCTACTAGTGGTAGATGGGTACTTAACAATGATTATTACAG   9300
Delta_2183060      AGAAGCTGGTGTTTGTGTATCTACTAGTGGTAGATGGGTACTTAACAATGATTATTACAG   9289
Omicron_7869197    AGAAGCTGGTGTTTGTGTATCTACTAGTGGTAGATGGGTACTTAACAATGATTATTACAG   9295
                   ************************************************************

w.t._745046        ATCTTTACCAGGAGTTTTCTGTGGTGTAGATGCTGTAAATTTACTTACTAATATGTTTAC   9356
Alpha_737204       ATCTTTACCAGGAGTTTTCTGTGGTGTAGATGCTGTAAATTTACTTACTAATATGTTTAC   9360
Delta_2183060      ATCTTTACCAGGAGTTTTCTGTGGTGTAGATGCTGTAAATTTACTTACTAATATGTTTAC   9349
Omicron_7869197    ATCTTTACCAGGAGTTTTCTGTGGTGTAGATGCTGTAAATTTACTTACTAATATGTTTAC   9355
                   ************************************************************

w.t._745046        ACCACTAATTCAACCTATTGGTGCTTTGGACATATCAGCATCTATAGTAGCTGGTGGTAT   9416
Alpha_737204       ACCACTAATTCAACCTATTGGTGCTTTGGACATATCAGCATCTATAGTAGCTGGTGGTAT   9420
Delta_2183060      ACCACTAATTCAACCTATTGGTGCTTTGGACATATCAGCATCTATAGTAGCTGGTGGTAT   9409
Omicron_7869197    ACCACTAATTCAACCTATTGGTGCTTTGGACATATCAGCATCTATAGTAGCTGGTGGTAT   9415
                   ************************************************************

w.t._745046        TGTAGCTATCGTAGTAACATGCCTTGCCTACTATTTTATGAGGTTTAGAAGAGCTTTTGG   9476
Alpha_737204       TGTAGCTATCGTAGTAACATGCCTTGCCTACTATTTTATGAGGTTTAGAAGAGCTTTTGG   9480
Delta_2183060      TGTAGCTATCGTAGTAACATGCCTTGCCTACTATTTTATGAGGTTTAGAAGAGCTTTTGG   9469
Omicron_7869197    TGTAGCTATCGTAGTAACATGCCTTGCCTACTATTTTATGAGGTTTAGAAGAGCTTTTGG   9475
                   ************************************************************

w.t._745046        TGAATACAGTCATGTAGTTGCCTTTAATACTTTACTATTCCTTATGTCATTCACTGTACT   9536
Alpha_737204       TGAATACAGTCATGTAGTTGCCTTTAATACTTTACTATTCCTTATGTCATTCACTGTACT   9540
Delta_2183060      TGAATACAGTCATGTAGTTGCCTTTAATACTTTACTATTCCTTATGTCATTCACTGTACT   9529
Omicron_7869197    TGAATACAGTCATGTAGTTGCCTTTAATACTTTACTATTCCTTATGTCATTCACTGTACT   9535
                   ************************************************************

w.t._745046        CTGTTTAACACCAGTTTACTCATTCTTACCTGGTGTTTATTCTGTTATTTACTTGTACTT   9596
Alpha_737204       CTGTTTAACACCAGTTTACTCATTCTTACCTGGTGTTTATTCTGTTATTTACTTGTACTT   9600
Delta_2183060      CTGTTTAACACCAGTTTACTCATTCTTACCTGGTGTTTATTCTGTTATTTACTTGTACTT   9589
Omicron_7869197    CTGTTTAACACCAGTTTACTCATTCTTACCTGGTGTTTATTCTGTTATTTACTTGTACTT   9595
                   ************************************************************
```

```
w.t._745046         GACATTTTATCTTACTAATGATGTTTCTTTTTTAGCACATATTCAGTGGATGGTTATGTT   9656
Alpha_737204        GACATTTTATCTTACTAATGATGTTTCTTTTTTAGCACATATTCAGTGGATGGTTATGTT   9660
Delta_2183060       GACATTTTATCTTACTAATGATGTTTCTTTTTTAGCACATATTCAGTGGATGGTTATGTT   9649
Omicron_7869197     GACATTTTATCTTACTAATGATGTTTCTTTTTTAGCACATATTCAGTGGATGGTTATGTT   9655
                    ************************************************************

w.t._745046         CACACCTTTAGTACCTTTCTGGATAACAATTGCTTATATCATTTGTATTTCCACAAAGCA   9716
Alpha_737204        CACACCTTTAGTACCTTTCTGGATAACAATTGCTTATATCATTTGTATTTCCACAAAGCA   9720
Delta_2183060       CACACCTTTAGTACCTTTCTGGATAACAATTGCTTATATCATTTGTATTTCCACAAAGCA   9709
Omicron_7869197     CACACCTTTAGTACCTTTCTGGATAACAATTGCTTATATCATTTGTATTTCCACAAAGCA   9715
                    ************************************************************

w.t._745046         TTTCTATTGGTTCTTTAGTAATTACCTAAAGAGACGTGTAGTCTTTAATGGTGTTTCCTT   9776
Alpha_737204        TTTCTATTGGTTCTTTAGTAATTACCTAAAGAGACGTGTAGTCTTTAATGGTGTTTCCTT   9780
Delta_2183060       TTTCTATTGGTTCTTTAGTAATTACCTAAAGAGACGTGTAGTCTTTAATGGTGTTTCCTT   9769
Omicron_7869197     TTTCTATTGGTTCTTTAGTAATTACCTAAAGAGACGTGTAGTCTTTAATGGTGTTTCCTT   9775
                    ************************************************************

w.t._745046         TAGTACTTTTGAAGAAGCTGCGCTGTGCACCTTTTTGTTAAATAAAGAAATGTATCTAAA   9836
Alpha_737204        TAGTACTTTTGAAGAAGCTGCGCTGTGCACCTTTTTGTTAAATAAAGAAATGTATCTAAA   9840
Delta_2183060       TAGTACTTTTGAAGAAGCTGCGCTGTGCACCTTTTTGTTAAATAAAGAAATGTATCTAAA   9829
Omicron_7869197     TAGTACTTTTGAAGAAGCTGCGCTGTGCACCTTTTTGTTAAATAAAGAAATGTATCTAAA   9835
                    ************************************************************

w.t._745046         GTTGCGTAGTGATGTGCTATTACCTCTTACGCAATATAATAGATACTTAGCTCTTTATAA   9896
Alpha_737204        GTTGCGTAGTGATGTGCTATTACCTCTTACGCAATATAATAGATACTTAGCTCTTTATAA   9900
Delta_2183060       GTTGCGTAGTGATGTGCTATTACCTCTTACGCAATATAATAGATACTTAGTTCTTTATAA   9889
Omicron_7869197     GTTGCGTAGTGATGTGCTATTACCTCTTACGCAATATAATAGATACTTAGCTCTTTATAA   9895
                    ************************************************** *********

w.t._745046         TAAGTACAAGTATTTTAGTGGAGCAATGGATACAACTAGCTACAGAGAAGCTGCTTGTTG   9956
Alpha_737204        TAAGTACAAGTATTTTAGTGGAGCAATGGATACAACTAGCTACAGAGAAGCTGCTTGTTG   9960
Delta_2183060       TAAGTACAAGTATTTTAGTGGAGCAATGGATACAACTAGCTACAGAGAAGCTGCTTGTTG   9949
Omicron_7869197     TAAGTACAAGTATTTTAGTGGAGCAATGGATACAACTAGCTACAGAGAAGCTGCTTGTTG   9955
                    ************************************************************

w.t._745046         TCATCTCGCAAAGGCTCTCAATGACCTCAGTAACTCAGGTTCTGATGTTCTTTACCAACC   10016
Alpha_737204        TCATCTCGCAAAGGCTCTCAATGACCTCAGTAACTCAGGTTCTGATGTTCTTTACCAACC   10020
Delta_2183060       TCATCTCGCAAAGGCTCTCAATGACCTCAGTAACTCAGGTTCTGATGTTCTTTACCAACC   10009
Omicron_7869197     TCATCTCGCAAAGGCTCTCAATGACCTCAGTAACTCAGGTTCTGATGTTCTTTACCAACC   10015
                    ************************************************************

w.t._745046         ACCACAAACCTCTATCACCTCAGCTGTTTTGCAGAGTGGTTTTAGAAAAATGGCATTCCC   10076
Alpha_737204        ACCACAAACCTCTATCACCTCAGCTGTTTTGCAGAGTGGTTTTAGAAAAATGGCATTCCC   10080
Delta_2183060       ACCACAAACCTCTATCACCTCAGCTGTTTTGCAGAGTGGTTTTAGAAAAATGGCATTCCC   10069
Omicron_7869197     ACCACAAATCTCTATCACCTCAGCTGTTTTGCAGAGTGGTTTTAGAAAAATGGCATTCCC   10075
                    ******** ***************************************************

w.t._745046         ATCTGGTAAAGTTGAGGGTTGTATGGTACAAGTAACTTGTGGTACAACTACACTTAACGG   10136
Alpha_737204        ATCTGGTAAAGTTGAGGGTTGTATGGTACAAGTAACTTGTGGTACAACTACACTTAACGG   10140
Delta_2183060       ATCTGGTAAAGTTGAGGGTTGTATGGTACAAGTAACTTGTGGTACAACTACACTTAACGG   10129
Omicron_7869197     ATCTGGTAAAGTTGAGGGTTGTATGGTACAAGTAACTTGTGGTACAACTACACTTAACGG   10135
                    ************************************************************

w.t._745046         TCTTTGGCTTGATGACGTAGTTTACTGTCCAAGACATGTGATCTGCACCTCTGAAGACAT   10196
Alpha_737204        TCTTTGGCTTGATGACGTAGTTTACTGTCCAAGACATGTGATCTGCACCTCTGAAGACAT   10200
Delta_2183060       TCTTTGGCTTGATGACGTAGTTTACTGTCCAAGACATGTGATCTGCACCTCTGAAGACAT   10189
Omicron_7869197     TCTTTGGCTTGATGACGTAGTTTACTGTCCAAGACATGTGATCTGCACCTCTGAAGACAT   10195
                    ************************************************************
```

```
w.t._745046         GCTTAACCCTAATTATGAAGATTTACTCATTCGTAAGTCTAATCATAATTTCTTGGTACA   10256
Alpha_737204        GCTTAACCCTAATTATGAAGATTTACTCATTCGTAAGTCTAATCATAATTTCTTGGTACA   10260
Delta_2183060       GCTTAACCCTAATTATGAAGATTTACTCATTCGTAAGTCTAATCATAATTTCTTGGTACA   10249
Omicron_7869197     GCTTAACCCTAATTATGAAGATTTACTCATTCGTAAGTCTAATCATAATTTCTTGGTACA   10255
                    ************************************************************

w.t._745046         GGCTGGTAATGTTCAACTCAGGGTTATTGGACATTCTATGCAAAATTGTGTACTTAAGCT   10316
Alpha_737204        GGCTGGTAATGTTCAACTCAGGGTTATTGGACATTCTATGCAAAATTGTGTACTTAAGCT   10320
Delta_2183060       GGCTGGTAATGTTCAACTCAGGGTTATTGGACATTCTATGCAAAATTGTGTACTTAAGCT   10309
Omicron_7869197     GGCTGGTAATGTTCAACTCAGGGTTATTGGACATTCTATGCAAAATTGTGTACTTAAGCT   10315
                    ************************************************************

w.t._745046         TAAGGTTGATACAGCCAATCCTAAGACACCTAAGTATAAGTTTGTTCGCATTCAACCAGG   10376
Alpha_737204        TAAGGTTGATACAGCCAATCCTAAGACACCTAAGTATAAGTTTGTTCGCATTCAACCAGG   10380
Delta_2183060       TAAGGTTGATACAGCCAATCCTAAGACACCTAAGTATAAGTTTGTTCGCATTCAACCAGG   10369
Omicron_7869197     TAAGGTTGATACAGCCAATCCTAAGACACCTAAGTATAAGTTTGTTCGCATTCAACCAGG   10375
                    ************************************************************

w.t._745046         ACAGACTTTTTCAGTGTTAGCTTGTTACAATGGTTCACCATCTGGTGTTTACCAATGTGC   10436
Alpha_737204        ACAGACTTTTTCAGTGTTAGCTTGTTACAATGGTTCACCATCTGGTGTTTACCAATGTGC   10440
Delta_2183060       ACAGACTTTTTCAGTGTTAGCTTGTTACAATGGTTCACCATCTGGTGTTTACCAATGTGC   10429
Omicron_7869197     ACAGACTTTTTCAGTGTTAGCTTGTTACAATGGTTCACCATCTGGTGTTTACCAATGTGC   10435
                    ************************************************************

w.t._745046         TATGAGGCCCAATTTCACTATTAAGGGTTCATTCCTTAATGGTTCATGTGGTAGTGTTGG   10496
Alpha_737204        TATGAGGCCCAATTTCACTATTAAGGGTTCATTCCTTAATGGTTCATGTGGTAGTGTTGG   10500
Delta_2183060       TATGAGGCCCAATTTCACTATTAAGGGTTCATTCCTTAATGGTTCATGTGGTAGTGTTGG   10489
Omicron_7869197     TATGAGGCACAATTTCACTATTAAGGGTTCATTCCTTAATGGTTCATGTGGTAGTGTTGG   10495
                    ******** ***************************************************

w.t._745046         TTTTAACATAGATTATGACTGTGTCTCTTTTTGTTACATGCACCATATGGAATTACCAAC   10556
Alpha_737204        TTTTAACATAGATTATGACTGTGTCTCTTTTTGTTACATGCACCATATGGAATTACCAAC   10560
Delta_2183060       TTTTAACATAGATTATGACTGTGTCTCTTTTTGTTACATGCACCATATGGAATTACCAAC   10549
Omicron_7869197     TTTTAACATAGATTATGACTGTGTCTCTTTTTGTTACATGCACCATATGGAATTACCAAC   10555
                    ************************************************************

w.t._745046         TGGAGTTCATGCTGGCACAGACTTAGAAGGTAACTTTTATGGACCTTTTGTTGACAGGCA   10616
Alpha_737204        TGGAGTTCATGCTGGCACAGACTTAGAAGGTAACTTTTATGGACCTTTTGTTGACAGGCA   10620
Delta_2183060       TGGAGTTCATGCTGGCACAGACTTAGAAGGTAACTTTTATGGACCTTTTGTTGACAGGCA   10609
Omicron_7869197     TGGAGTTCATGCTGGCACAGACTTAGAAGGTAACTTTTATGGACCTTTTGTTGACAGGCA   10615
                    ************************************************************

w.t._745046         AACAGCACAAGCAGCTGGTACGGACACAACTATTACAGTTAATGTTTTAGCTTGGTTGTA   10676
Alpha_737204        AACAGCACAAGCAGCTGGTACGGACACAACTATTACAGTTAATGTTTTAGCTTGGTTGTA   10680
Delta_2183060       AACAGCACAAGCAGCTGGTACGGACACAACTATTACAGTTAATGTTTTAGCTTGGTTGTA   10669
Omicron_7869197     AACAGCACAAGCAGCTGGTACGGACACAACTATTACAGTTAATGTTTTAGCTTGGTTGTA   10675
                    ************************************************************

w.t._745046         CGCTGCTGTTATAAATGGAGACAGGTGGTTTCTCAATCGATTTACCACAACTCTTAATGA   10736
Alpha_737204        CGCTGCTGTTATAAATGGAGACAGGTGGTTTCTCAATCGATTTACCACAACTCTTAATGA   10740
Delta_2183060       CGCTGCTGTTATAAATGGAGACAGGTGGTTTCTCAATCGATTTACCACAACTCTTAATGA   10729
Omicron_7869197     CGCTGCTGTTATAAATGGAGACAGGTGGTTTCTCAATCGATTTACCACAACTCTTAATGA   10735
                    ************************************************************

w.t._745046         CTTTAACCTTGTGGCTATGAAGTACAATTATGAACCTCTAACACAAGACCATGTTGACAT   10796
Alpha_737204        CTTTAACCTTGTGGCTATGAAGTACAATTATGAACCTCTAACACAAGACCATGTTGACAT   10800
Delta_2183060       CTTTAACCTTGTGGCTATGAAGTACAATTATGAACCTCTAACACAAGACCATGTTGACAT   10789
Omicron_7869197     CTTTAACCTTGTGGCTATGAAGTACAATTATGAACCTCTAACACAAGACCATGTTGACAT   10795
                    ************************************************************
```

```
w.t._745046         ACTAGGACCTCTTTCTGCTCAAACTGGAATTGCCGTTTTAGATATGTGTGCCTCATTAAA   10856
Alpha_737204        ACTAGGACCTCTTTCTGCTCAAACTGGAATTGCCGTTTTAGATATGTGTGCCTCATTAAA   10860
Delta_2183060       ACTAGGACCTCTTTCTGCTCAAACTGGAATTGCCGTTTTAGATATGTGTGCCTCATTAAA   10849
Omicron_7869197     ACTAGGACCTCTTTCTGCTCAAACTGGAATTGCCGTTTTAGATATGTGTGCCTCATTAAA   10855
                    ************************************************************

w.t._745046         AGAATTACTGCAAAATGGTATGAATGGACGTACCATATTGGGTAGTGCTTTATTAGAAGA   10916
Alpha_737204        AGAATTACTGCAAAATGGTATGAATGGACGTACCATATTGGGTAGTGCTTTATTAGAAGA   10920
Delta_2183060       AGAATTACTGCAAAATGGTATGAATGGACGTACCATATTGGGTAGTGCTTTATTAGAAGA   10909
Omicron_7869197     AGAATTACTGCAAAATGGTATGAATGGACGTACCATATTGGGTAGTGCTTTATTAGAAGA   10915
                    ************************************************************

w.t._745046         TGAATTTACACCTTTTGATGTTGTTAGACAATGCTCAGGTGTTACTTTCCAAAGTGCAGT   10976
Alpha_737204        TGAATTTACACCTTTTGATGTTGTTAGACAATGCTCAGGTGTTACTTTCCAAAGTGCAGT   10980
Delta_2183060       TGAATTTACACCTTTTGATGTTGTTAGACAATGCTCAGGTGTTACTTTCCAAAGTGCAGT   10969
Omicron_7869197     TGAATTTACACCTTTTGATGTTGTTAGACAATGCTCAGGTGTTACTTTCCAAAGTGCAGT   10975
                    ************************************************************

w.t._745046         GAAAAGAACAATCAAGGGTACACACCACTGGTTGTTACTCACAATTTTGACCTCACTTTT   11036
Alpha_737204        GAAAAGAACAATCAAGGGTACACACCACTGGTTGTTACTCACAATTTTGACCTCACTTTT   11040
Delta_2183060       GAAAAGAACAATCAAGGGTACACACCACTGGTTGTTACTCACAATTTTGACCTCACTTTT   11029
Omicron_7869197     GAAAAGAACAATCAAGGGTACACACCACTGGTTGTTACTCACAATTTTGACCTCACTTTT   11035
                    ************************************************************

w.t._745046         AGTTTTAGTCCAGAGTACTCAATGGTCTTTGTTCTTTTTTTTGTATGAAAATGCCTTTTT   11096
Alpha_737204        AGTTTTAGTCCAGAGTACTCAATGGTCTTTGTTCTTTTTTTTGTATGAAAATGCCTTTTT   11100
Delta_2183060       AGTTTTAGTCCAGAGTACTCAATGGTCTTTGTTCTTTTTTTTGTATGAAAATGCCTTTTT   11089
Omicron_7869197     AGTTTTAGTCCAGAGTACTCAATGGTCTTTGTTCTTTTTTTTGTATGAAAATGCCTTTTT   11095
                    ************************************************************

w.t._745046         ACCTTTTGCTATGGGTATTATTGCTATGTCTGCTTTTGCAATGATGTTTGTCAAACATAA   11156
Alpha_737204        ACCTTTTGCTATGGGTATTATTGCTATGTCTGCTTTTGCAATGATGTTTGTCAAACATAA   11160
Delta_2183060       ACCTTTTGCTATGGGTATTATTGCTATGTCTGCTTTTGCAATGATGTTTGTCAAACATAA   11149
Omicron_7869197     ACCTTTTGCTATGGGTATTATTGCTATGTCTGCTTTTGCAATGATGTTTGTCAAACATAA   11155
                    ************************************************************

w.t._745046         GCATGCATTTCTCTGTTTGTTTTTGTTACCCTCTCTTGCCACTGTAGCTTATTTTAATAT   11216
Alpha_737204        GCATGCATTTCTCTGTTTGTTTTTGTTACCCTCTCTTGCCACTGTAGCTTATTTTAATAT   11220
Delta_2183060       GCATGCATTTCTCTGTTTGTTTTTGTTACCCTCTCTTGCCACTGTAGCTTATTTTAATAT   11209
Omicron_7869197     GCATGCATTTCTCTGTTTGTTTTTGTTACCCTCTCTTGCCACTGTAGCTTATTTTAATAT   11215
                    ************************************************************

w.t._745046         GGTCTATATGCCTGCTAGTTGGGTGATGCGTATTATGACATGGTTGGATATGGTTGATAC   11276
Alpha_737204        GGTCTATATGCCTGCTAGTTGGGTGATGCGTATTATGACATGGTTGGATATGGTTGATAC   11280
Delta_2183060       GGTCTATATGCCTGCTAGTTGGGTGATGCGTATTATGACATGGTTGGATATGGTTGATAC   11269
Omicron_7869197     GGTCTATATGCCTGCTAGTTGGGTGATGCGTATTATGACATGGTTGGATATGGTTGATAC   11275
                    ************************************************************

w.t._745046         TAGTTTGTCTGGTTTTAAGCTAAAAGACTGTGTTATGTATGCATCAGCTGTAGTGTTACT   11336
Alpha_737204        TAGTTTG---------AAGCTAAAAGACTGTGTTATGTATGCATCAGCTGTAGTGTTACT   11331
Delta_2183060       TAGTTTGTCTGGTTTTAAGCTAAAAGACTGTGTTATGTATGCATCAGCTGTAGTGTTACT   11329
Omicron_7869197     TAGTTTT---------AAGCTAAAAGACTGTGTTATGTATGCATCAGCTGTAGTGTTACT   11326
                    ******          ********************************************

w.t._745046         AATCCTTATGACAGCAAGAACTGTGTATGATGATGGTGCTAGGAGAGTGTGGACACTTAT   11396
Alpha_737204        AATCCTTATGACAGCAAGAACTGTGTATGATGATGGTGCTAGGAGAGTGTGGACACTTAT   11391
Delta_2183060       AATCCTTATGACAGCAAGAACTGTGTATGATGATGGTGCTAGGAGAGTGTGGACACTTAT   11389
Omicron_7869197     AATCCTTATGACAGCAAGAACTGTGTATGATGATGGTGCTAGGAGAGTGTGGACACTTAT   11386
                    ************************************************************
```

```
w.t._745046         GAATGTCTTGACACTCGTTTATAAAGTTTATTATGGTAATGCTTTAGATCAAGCCATTTC   11456
Alpha_737204        GAATGTCTTGACACTCGTTTATAAAGTTTATTATGGTAATGCTTTAGATCAAGCCATTTC   11451
Delta_2183060       GAATGTCTTGACACTCGCTTATAAAGTTTATTATGGTAATGCTTTAGATCAAGCCATTTC   11449
Omicron_7869197     GAATGTCTTGACACTCGTTTATAAAGTTTATTATGGTAATGCTTTAGATCAAGCCATTTC   11446
                    ***************** ******************************************

w.t._745046         CATGTGGGCTCTTATAATCTCTGTTACCTCTAACTACTCAGGTGTAGTTACAACTGTCAT   11516
Alpha_737204        CATGTGGGCTCTTATAATCTCTGTTACCTCTAACTACTCAGGTGTAGTTACAACTGTCAT   11511
Delta_2183060       CATGTGGGCTCTTATAATCTCTGTTACCTCTAACTACTCAGGTGTAGTTACAACTGTCAT   11509
Omicron_7869197     CATGTGGGCTCTTATAATCTCTGTTACCTCTAACTACTCAGGTGTAGTTACAACTGTCAT   11506
                    ************************************************************

w.t._745046         GTTTTTGGCCAGAGGTATTTTTTTTATGTGTGTTGAGTATTGCCCTATTTTCCTCATAAC   11576
Alpha_737204        GTTTTTGGCCAGAGGTATTGTTTTTATGTGTGTTGAGTATTGCCCTATTTTCCTCATAAC   11571
Delta_2183060       GTTTTTGGCCAGAGGTATTGTTTTTATGTGTGTTGAGTATTGCCCTATTTTCCTCATAAC   11569
Omicron_7869197     GTTTTTGGCCAGAGGTGTTGTTTTTATGTGTGTTGAGTATTGCCCTATTTTCCTCATAAC   11566
                    **************** ** ****************************************

w.t._745046         TGGTAATACACCTCAGTGTATAATGCTAGTTTATTGTTTCTTAGGCTATTTTTGTACTTG   11636
Alpha_737204        TGGTAATACACCTCAGTGTATAATGCTAGTTTATTGTTTCTTAGGCTATTTTTGTACTTG   11631
Delta_2183060       TGGTAATACACCTCAGTGTATAATGCTAGTTTATTGTTTCTTAGGCTATTTTTGTACTTG   11629
Omicron_7869197     TGGTAATACACCTCAGTGTATAATGCTAGTTTATTGTTTCTTAGGCTATTTTTGTACTTG   11626
                    ************************************************************

w.t._745046         TTACTTTGGCCTCTTTTGTTTACTCAACCGCTACTTTAGACTGACTCTTGGTGTTTATGA   11696
Alpha_737204        TTACTTTGGCCTCTTTTGTTTACTCAACCGCTACTTTAGACTGACTCTTGGTGTTTATGA   11691
Delta_2183060       TTACTTTGGCCTCTTTTGTTTACTCAACCGCTACTTTAGACTGACTCTTGGTGTTTATGA   11689
Omicron_7869197     TTACTTTGGCCTCTTTTGTTTACTCAACCGCTACTTTAGACTGACTCTTGGTGTTTATGA   11686
                    ************************************************************

w.t._745046         TTACTTAGTTTCTACACAGGAGTTTAGATATATGAATTCACAGGGACTACTCCCACCCAA   11756
Alpha_737204        TTACTTAGTTTCTACACAGGAGTTTAGATATATGAATTCACAGGGACTACTCCCACCCAA   11751
Delta_2183060       TTACTTAGTTTCTACACAGGAGTTTAGATATATGAATTCACAGGGACTACTCCCACCCAA   11749
Omicron_7869197     TTACTTAGTTTCTACACAGGAGTTTAGATATATGAATTCACAGGGACTACTCCCACCCAA   11746
                    ************************************************************

w.t._745046         GAATAGCATAGATGCCCTCAAACTCAACATTAAATTGTTGGGTGTTGGTGGCAAACCTTG   11816
Alpha_737204        GAATAGCATAGATGCCCTCAAACTCAACATTAAATTGTTGGGTGTTGGTGGCAAACCTTG   11811
Delta_2183060       GAATAGCATAGATGCCCTCAAACTCAACATTAAATTGTTGGGTGTTGGTGGCAAACCTTG   11809
Omicron_7869197     GAATAGCATAGATGCCCTCAAACTCAACATTAAATTGTTGGGTGTTGGTGGCAAACCTTG   11806
                    ************************************************************

w.t._745046         TATCAAAGTAGCCACTGTACAGTCTAAAATGTCAGATGTAAAGTGCACATCAGTAGTCTT   11876
Alpha_737204        TATCAAAGTAGCCACTGTACAGTCTAAAATGTCAGATGTAAAGTGCACATCAGTAGTCTT   11871
Delta_2183060       TATCAAAGTAGCCACTGTACAGTCTAAAATGTCAGATGTAAAGTGCACATCAGTAGTCTT   11869
Omicron_7869197     TATCAAAGTAGCCACTGTACAGTCTAAAATGTCAGATGTAAAGTGCACATCAGTAGTCTT   11866
                    ************************************************************

w.t._745046         ACTCTCAGTTTTGCAACAACTCAGAGTAGAATCATCATCTAAATTGTGGGCTCAATGTGT   11936
Alpha_737204        ACTCTCAGTTTTGCAACAACTCAGAGTAGAATCATCATCTAAATTGTGGGCTCAATGTGT   11931
Delta_2183060       ACTCTCAGTTTTGCAACAACTCAGAGTAGAATCATCATCTAAATTGTGGGCTCAATGTGT   11929
Omicron_7869197     ACTCTCAGTTTTGCAACAACTCAGAGTAGAATCATCATCTAAATTGTGGGCTCAATGTGT   11926
                    ************************************************************

w.t._745046         CCAGTTACACAATGACATTCTCTTAGCTAAAGATACTACTGAAGCCTTTGAAAAAATGGT   11996
Alpha_737204        CCAGTTACACAATGACATTCTCTTAGCTAAAGATACTACTGAAGCCTTTGAAAAAATGGT   11991
Delta_2183060       CCAGTTACACAATGACATTCTCTTAGCTAAAGATACTACTGAAGCCTTTGAAAAAATGGT   11989
Omicron_7869197     CCAGTTACACAATGACATTCTCTTAGCTAAAGATACTACTGAAGCCTTTGAAAAAATGGT   11986
                    ************************************************************
```

```
w.t._745046        TTCACTACTTTCTGTTTTGCTTTCCATGCAGGGTGCTGTAGACATAAACAAGCTTTGTGA   12056
Alpha_737204       TTCACTACTTTCTGTTTTGCTTTCCATGCAGGGTGCTGTAGACATAAACAAGCTTTGTGA   12051
Delta_2183060      TTCACTACTTTCTGTTTTGCTTTCCATGCAGGGTGCTGTAGACATAAACAAGCTTTGTGA   12049
Omicron_7869197    TTCACTACTTTCTGTTTTGCTTTCCATGCAGGGTGCTGTAGACATAAACAAGCTTTGTGA   12046
                   ************************************************************

w.t._745046        AGAAATGCTGGACAACAGGGCAACCTTACAAGCTATAGCCTCAGAGTTTAGTTCCCCTCC   12116
Alpha_737204       AGAAATGCTGGACAACAGGGCAACCTTACAAGCTATAGCCTCAGAGTTTAGTTCCCCTCC   12111
Delta_2183060      AGAAATGCTGGACAACAGGGCAACCTTACAAGCTATAGCCTCAGAGTTTAGTTCCCCTCC   12109
Omicron_7869197    AGAAATGCTGGACAACAGGGCAACCTTACAAGCTATAGCCTCAGAGTTTAGTTCCCCTCC   12106
                   ************************************************************

w.t._745046        ATCATATGCAGCTTTTGCTACTGCTCAAGAAGCTTATGAGCAGGCTGTTGCTAATGGTGA   12176
Alpha_737204       ATCATATGCAGCTTTTGCTACTGCTCAAGAAGCTTATGAGCAGGCTGTTGCTAATGGTGA   12171
Delta_2183060      ATCATATGCAGCTTTTGCTACTGCTCAAGAAGCTTATGAGCAGGCTGTTGCTAATGGTGA   12169
Omicron_7869197    ATCATATGCAGCTTTTGCTACTGCTCAAGAAGCTTATGAGCAGGCTGTTGCTAATGGTGA   12166
                   ************************************************************

w.t._745046        TTCTGAAGTTGTTCTTAAAAAGTTGAAGAAGTCTTTGAATGTGGCTAAATCTGAATTTGA   12236
Alpha_737204       TTCTGAAGTTGTTCTTAAAAAGTTGAAGAAGTCTTTGAATGTGGCTAAATCTGAATTTGA   12231
Delta_2183060      TTCTGAAGTTGTTCTTAAAAAGTTGAAGAAGTCTTTGAATGTGGCTAAATCTGAATTTGA   12229
Omicron_7869197    TTCTGAAGTTGTTCTTAAAAAGTTGAAGAAGTCTTTGAATGTGGCTAAATCTGAATTTGA   12226
                   ************************************************************

w.t._745046        CCGTGATGCAGCCATGCAACGTAAGTTGGAAAAGATGGCTGATCAAGCTATGACCCAAAT   12296
Alpha_737204       CCGTGATGCAGCCATGCAACGTAAGTTGGAAAAGATGGCTGATCAAGCTATGACCCAAAT   12291
Delta_2183060      CCGTGATGCAGCCATGCAACGTAAGTTGGAAAAGATGGCTGATCAAGCTATGACCCAAAT   12289
Omicron_7869197    CCGTGATGCAGCCATGCAACGTAAGTTGGAAAAGATGGCTGATCAAGCTATGACCCAAAT   12286
                   ************************************************************

w.t._745046        GTATAAACAGGCTAGATCTGAGGACAAGAGGGCAAAAGTTACTAGTGCTATGCAGACAAT   12356
Alpha_737204       GTATAAACAGGCTAGATCTGAGGACAAGAGGGCAAAAGTTACTAGTGCTATGCAGACAAT   12351
Delta_2183060      GTATAAACAGGCTAGATCTGAGGACAAGAGGGCAAAAGTTACTAGTGCTATGCAGACAAT   12349
Omicron_7869197    GTATAAACAGGCTAGATCTGAGGACAAGAGGGCAAAAGTTACTAGTGCTATGCAGACAAT   12346
                   ************************************************************

w.t._745046        GCTTTTCACTATGCTTAGAAAGTTGGATAATGATGCACTCAACAACATTATCAACAATGC   12416
Alpha_737204       GCTTTTCACTATGCTTAGAAAGTTGGATAATGATGCACTCAACAACATTATCAACAATGC   12411
Delta_2183060      GCTTTTCACTATGCTTAGAAAGTTGGATAATGATGCACTCAACAACATTATCAACAATGC   12409
Omicron_7869197    GCTTTTCACTATGCTTAGAAAGTTGGATAATGATGCACTCAACAACATTATCAACAATGC   12406
                   ************************************************************

w.t._745046        AAGAGATGGTTGTGTTCCCTTGAACATAATACCTCTTACAACAGCAGCCAAACTAATGGT   12476
Alpha_737204       AAGAGATGGTTGTGTTCCCTTGAACATAATACCTCTTACAACAGCAGCCAAACTAATGGT   12471
Delta_2183060      AAGAGATGGTTGTGTTCCCTTGAACATAATACCTCTTACAACAGCAGCCAAACTAATGGT   12469
Omicron_7869197    AAGAGATGGTTGTGTTCCCTTGAACATAATACCTCTTACAACAGCAGCCAAACTAATGGT   12466
                   ************************************************************

w.t._745046        TGTCATACCAGACTATAACACATATAAAAATACGTGTGATGGTACAACATTTACTTATGC   12536
Alpha_737204       TGTCATACCAGACTATAACACATATAAAAATACGTGTGATGGTACAACATTTACTTATGC   12531
Delta_2183060      TGTCATACCAGACTATAACACATATAAAAATACGTGTGATGGTACAACATTTACTTATGC   12529
Omicron_7869197    TGTCATACCAGACTATAACACATATAAAAATACGTGTGATGGTACAACATTTACTTATGC   12526
                   ************************************************************

w.t._745046        ATCAGCATTGTGGGAAATCCAACAGGTTGTAGATGCAGATAGTAAAATTGTTCAACTTAG   12596
Alpha_737204       ATCAGCATTGTGGGAAATCCAACAGGTTGTAGATGCAGATAGTAAAATTGTTCAACTTAG   12591
Delta_2183060      ATCAGCATTGTGGGAAATCCAACAGGTTGTAGATGCAGATAGTAAAATTGTTCAACTTAG   12589
Omicron_7869197    ATCAGCATTGTGGGAAATCCAACAGGTTGTAGATGCAGATAGTAAAATTGTTCAACTTAG   12586
                   ************************************************************
```

```
w.t._745046        TGAAATTAGTATGGACAATTCACCTAATTTAGCATGGCCTCTTATTGTAACAGCTTTAAG   12656
Alpha_737204       TGAAATTAGTATGGACAATTCACCTAATTTAGCATGGCCTCTTATTGTAACAGCTTTAAG   12651
Delta_2183060      TGAAATTAGTATGGACAATTCACCTAATTTAGCATGGCCTCTTATTGTAACAGCTTTAAG   12649
Omicron_7869197    TGAAATTAGTATGGACAATTCACCTAATTTAGCATGGCCTCTTATTGTAACAGCTTTAAG   12646
                   ************************************************************

w.t._745046        GGCCAATTCTGCTGTCAAATTACAGAATAATGAGCTTAGTCCTGTTGCACTACGACAGAT   12716
Alpha_737204       GGCCAATTCTGCTGTCAAATTACAGAATAATGAGCTTAGTCCTGTTGCACTACGACAGAT   12711
Delta_2183060      GGCCAATTCTGCTGTCAAATTACAGAATAATGAGCTTAGTCCTGTTGCACTACGACAGAT   12709
Omicron_7869197    GGCCAATTCTGCTGTCAAATTACAGAATAATGAGCTTAGTCCTGTTGCACTACGACAGAT   12706
                   ************************************************************

w.t._745046        GTCTTGTGCTGCCGGTACTACACAAACTGCTTGCACTGATGACAATGCGTTAGCTTACTA   12776
Alpha_737204       GTCTTGTGCTGCCGGTACTACACAAACTGCTTGCACTGATGACAATGCGTTAGCTTACTA   12771
Delta_2183060      GTCTTGTGCTGCCGGTACTACACAAACTGCTTGCACTGATGACAATGCGTTAGCTTACTA   12769
Omicron_7869197    GTCTTGTGCTGCCGGTACTACACAAACTGCTTGCACTGATGACAATGCGTTAGCTTACTA   12766
                   ************************************************************

w.t._745046        CAACACAACAAAGGGAGGTAGGTTTGTACTTGCACTGTTATCCGATTTACAGGATTTGAA   12836
Alpha_737204       CAACACAACAAAGGGAGGTAGGTTTGTACTTGCACTGTTATCCGATTTACAGGATTTGAA   12831
Delta_2183060      CAACACAACAAAGGGAGGTAGGTTTGTACTTGCACTGTTATCCGATTTACAGGATTTGAA   12829
Omicron_7869197    CAACACAACAAAGGGAGGTAGGTTTGTACTTGCACTGTTATCCGATTTACAGGATTTGAA   12826
                   ************************************************************

w.t._745046        ATGGGCTAGATTCCCTAAGAGTGATGGAACTGGTACTATCTATACAGAACTGGAACCACC   12896
Alpha_737204       ATGGGCTAGATTCCCTAAGAGTGATGGAACTGGTACTATCTATACAGAACTGGAACCACC   12891
Delta_2183060      ATGGGCTAGATTCCCTAAGAGTGATGGAACTGGTACTATCTATACAGAACTGGAACCACC   12889
Omicron_7869197    ATGGGCTAGATTCCCTAAGAGTGATGGAACTGGTACTATCTATACAGAACTGGAACCACC   12886
                   ************************************************************

w.t._745046        TTGTAGGTTTGTTACAGACACACCTAAAGGTCCTAAAGTGAAGTATTTATACTTTATTAA   12956
Alpha_737204       TTGTAGGTTTGTTACAGACACACCTAAAGGTCCTAAAGTGAAGTATTTATACTTTATTAA   12951
Delta_2183060      TTGTAGGTTTGTTACAGACACACCTAAAGGTCCTAAAGTGAAGTACTTATACTTTATTAA   12949
Omicron_7869197    TTGTAGGTTTGTTACAGACACACCTAAAGGTCCTAAAGTGAAGTATTTATACTTTATTAA   12946
                   ******************************************** ***************

w.t._745046        AGGATTAAACAACCTAAATAGAGGTATGGTACTTGGTAGTTTAGCTGCCACAGTACGTCT   13016
Alpha_737204       AGGATTAAACAACCTAAATAGAGGTATGGTACTTGGTAGTTTAGCTGCCACAGTACGTCT   13011
Delta_2183060      AGGATTAAACAACCTAAATAGAGGTATGGTACTTGGTAGTTTAGCTGCCACAGTACGTCT   13009
Omicron_7869197    AGGATTAAACAACCTAAATAGAGGTATGGTACTTGGTAGTTTAGCTGCCACAGTACGTCT   13006
                   ************************************************************

w.t._745046        ACAAGCTGGTAATGCAACAGAAGTGCCTGCCAATTCAACTGTATTATCTTTCTGTGCTTT   13076
Alpha_737204       ACAAGCTGGTAATGCAACAGAAGTGCCTGCCAATTCAACTGTATTATCTTTCTGTGCTTT   13071
Delta_2183060      ACAAGCTGGTAATGCAACAGAAGTGCCTGCCAATTCAACTGTATTATCTTTCTGTGCTTT   13069
Omicron_7869197    ACAAGCTGGTAATGCAACAGAAGTGCCTGCCAATTCAACTGTATTATCTTTCTGTGCTTT   13066
                   ************************************************************

w.t._745046        TGCTGTAGATGCTGCTAAAGCTTACAAAGATTATCTAGCTAGTGGGGGACAACCAATCAC   13136
Alpha_737204       TGCTGTAGATGCTGCTAAAGCTTACAAAGATTATCTAGCTAGTGGGGGACAACCAATCAC   13131
Delta_2183060      TGCTGTAGATGCTGCTAAAGCTTACAAAGATTATCTAGCTAGTGGGGGACAACCAATCAC   13129
Omicron_7869197    TGCTGTAGATGCTGCTAAAGCTTACAAAGATTATCTAGCTAGTGGGGGACAACCAATCAC   13126
                   ************************************************************

w.t._745046        TAATTGTGTTAAGATGTTGTGTACACACACTGGTACTGGTCAGGCAATAACAGTTACACC   13196
Alpha_737204       TAATTGTGTTAAGATGTTGTGTACACACACTGGTACTGGTCAGGCAATAACAGTTACACC   13191
Delta_2183060      TAATTGTGTTAAGATGTTGTGTACACACACTGGTACTGGTCAGGCAATAACAGTTACACC   13189
Omicron_7869197    TAATTGTGTTAAGATGTTGTGTACACACACTGGTACTGGTCAGGCAATAACAGTCACACC   13186
                   ****************************************************** *****
```

```
w.t._745046         GGAAGCCAATATGGATCAAGAATCCTTTGGTGGTGCATCGTGTTGTCTGTACTGCCGTTG   13256
Alpha_737204        GGAAGCCAATATGGATCAAGAATCCTTTGGTGGTGCATCGTGTTGTCTGTACTGCCGTTG   13251
Delta_2183060       GGAAGCCAATATGGATCAAGAATCCTTTGGTGGTGCATCGTGTTGTCTGTACTGCCGTTG   13249
Omicron_7869197     GGAAGCCAATATGGATCAAGAATCCTTTGGTGGTGCATCGTGTTGTCTGTACTGCCGTTG   13246
                    ************************************************************

w.t._745046         CCACATAGATCATCCAAATCCTAAAGGATTTTGTGACTTAAAAGGTAAGTATGTACAAAT   13316
Alpha_737204        CCACATAGATCATCCAAATCCTAAAGGATTTTGTGACTTAAAAGGTAAGTATGTACAAAT   13311
Delta_2183060       CCACATAGATCATCCAAATCCTAAAGGATTTTGTGACTTAAAAGGTAAGTATGTACAAAT   13309
Omicron_7869197     CCACATAGATCATCCAAATCCTAAAGGATTTTGTGACTTAAAAGGTAAGTATGTACAAAT   13306
                    ************************************************************

w.t._745046         ACCTACAACTTGTGCTAATGACCCTGTGGGTTTTACACTTAAAAACACAGTCTGTACCGT   13376
Alpha_737204        ACCTACAACTTGTGCTAATGACCCTGTGGGTTTTACACTTAAAAACACAGTCTGTACCGT   13371
Delta_2183060       ACCTACAACTTGTGCTAATGACCCTGTGGGTTTTACACTTAAAAACACAGTCTGTACCGT   13369
Omicron_7869197     ACCTACAACTTGTGCTAATGACCCTGTGGGTTTTACACTTAAAAACACAGTCTGTACCGT   13366
                    ************************************************************

w.t._745046         CTGCGGTATGTGGAAAGGTTATGGCTGTAGTTGTGATCAACTCCGCGAACCCATGCCTCA   13436
Alpha_737204        CTGCGGTATGTGGAAAGGTTATGGCTGTAGTTGTGATCAACTCCGCGAACCCATGCCTCA   13431
Delta_2183060       CTGCGGTATGTGGAAAGGTTATGGCTGTAGTTGTGATCAACTCCGCGAACCCATGCCTCA   13429
Omicron_7869197     CTGCGGTATGTGGAAAGGTTATGGCTGTAGTTGTGATCAACTCCGCGAACCCATGCCTCA   13426
                    ************************************************************

w.t._745046         GTCAGCTGATGCACAATCGTTTTTAAACGGGTTTGCGGTGTAAGTGCAGCCCGTCTTACA   13496
Alpha_737204        GTCAGCTGATGCACAATCGTTTTTAAACGGGTTTGCGGTGTAAGTGCAGCCCGTCTTACA   13491
Delta_2183060       GTCAGCTGATGCACAATCGTTTTTAAACGGGTTTGCGGTGTAAGTGCAGCCCGTCTTACA   13489
Omicron_7869197     GTCAGCTGATGCACAATCGTTTTTAAACGGGTTTGCGGTGTAAGTGCAGCCCGTCTTACA   13486
                    ************************************************************

w.t._745046         CCGTGCGGCACAGGCACTAGTACTGATGTCGTATACAGGGCTTTTGACATCTACAATGAT   13556
Alpha_737204        CCGTGCGGCACAGGCACTAGTACTGATGTCGTATACAGGGCTTTTGACATCTACAATGAT   13551
Delta_2183060       CCGTGCGGCACAGGCACTAGTACTGATGTCGTATACAGGGCTTTTGACATCTACAATGAT   13549
Omicron_7869197     CCGTGCGGCACAGGCACTAGTACTGATGTCGTATACAGGGCTTTTGACATCTACAATGAT   13546
                    ************************************************************

w.t._745046         AAAGTAGCTGGTTTTGCTAAATTCCTAAAAACTAATTGTTGTCGCCTCCAAGAAAAGGAC   13616
Alpha_737204        AAAGTAGCTGGTTTTGCTAAATTCCTAAAAACTAATTGTTGTCGCCTCCAAGAAAAGGAC   13611
Delta_2183060       AAAGTAGCTGGTTTTGCTAAATTCCTAAAAACTAATTGTTGTCGCCTCCAAGAAAAGGAC   13609
Omicron_7869197     AAAGTAGCTGGTTTTGCTAAATTCCTAAAAACTAATTGTTGTCGCCTCCAAGAAAAGGAC   13606
                    ************************************************************

w.t._745046         GAAGATGACAATTTAATTGATTCTTACTTTGTAGTTAAGAGACACACTTTCTCTAACTAC   13676
Alpha_737204        GAAGATGACAATTTAATTGATTCTTACTTTGTAGTTAAGAGACACACTTTCTCTAACTAC   13671
Delta_2183060       GAAGATGACAATTTAATTGATTCTTACTTTGTAGTTAAGAGACACACTTTCTCTAACTAC   13669
Omicron_7869197     GAAGATGACAATTTAATTGATTCTTACTTTGTAGTTAAGAGACACACTTTCTCTAACTAC   13666
                    ************************************************************

w.t._745046         CAACATGAAGAAACAATTTATAATTTACTTAAGGATTGTCCAGCTGTTGCTAAACATGAC   13736
Alpha_737204        CAACATGAAGAAACAATTTATAATTTACTTAAGGATTGTCCAGCTGTTGCTAAACATGAC   13731
Delta_2183060       CAACATGAAGAAACAATTTATAATTTACTTAAGGATTGTCCAGCTGTTGCTAAACATGAC   13729
Omicron_7869197     CAACATGAAGAAACAATTTATAATTTACTTAAGGATTGTCCAGCTGTTGCTAAACATGAC   13726
                    ************************************************************

w.t._745046         TTCTTTAAGTTTAGAATAGACGGTGACATGGTACCACATATATCACGTCAACGTCTTACT   13796
Alpha_737204        TTCTTTAAGTTTAGAATAGACGGTGACATGGTACCACATATATCACGTCAACGTCTTACT   13791
Delta_2183060       TTCTTTAAGTTTAGAATAGACGGTGACATGGTACCACATATATCACGTCAACGTCTTACT   13789
Omicron_7869197     TTCTTTAAGTTTAGAATAGACGGTGACATGGTACCACATATATCACGTCAACGTCTTACT   13786
                    ************************************************************
```

```
w.t._745046         AAATACACAATGGCAGACCTCGTCTATGCTTTAAGGCATTTTGATGAAGGTAATTGTGAC   13856
Alpha_737204        AAATACACAATGGCAGACCTCGTCTATGCTTTAAGGCATTTTGATGAAGGTAATTGTGAC   13851
Delta_2183060       AAATACACAATGGCAGACCTCGTCTATGCTTTAAGGCATTTTGATGAAGGTAATTGTGAC   13849
Omicron_7869197     AAATACACAATGGCAGACCTCGTCTATGCTTTAAGGCATTTTGATGAAGGTAATTGTGAC   13846
                    ************************************************************

w.t._745046         ACATTAAAAGAAATACTTGTCACATACAATTGTTGTGATGATGATTATTTCAATAAAAAG   13916
Alpha_737204        ACATTAAAAGAAATACTTGTCACATACAATTGTTGTGATGATGATTATTTCAATAAAAAG   13911
Delta_2183060       ACATTAAAAGAAATACTTGTCACATACAATTGTTGTGATGATGATTATTTCAATAAAAAG   13909
Omicron_7869197     ACATTAAAAGAAATACTTGTCACATACAATTGTTGTGATGATGATTATTTCAATAAAAAG   13906
                    ************************************************************

w.t._745046         GACTGGTATGATTTTGTAGAAAACCCAGATATATTACGCGTATACGCCAACTTAGGTGAA   13976
Alpha_737204        GACTGGTATGATTTTGTAGAAAACCCAGATATATTACGCGTATACGCCAACTTAGGTGAA   13971
Delta_2183060       GACTGGTATGATTTTGTAGAAAACCCAGATATATTACGCGTATACGCCAACTTAGGTGAA   13969
Omicron_7869197     GACTGGTATGATTTTGTAGAAAACCCAGATATATTACGCGTATACGCCAACTTAGGTGAA   13966
                    ************************************************************

w.t._745046         CGTGTACGCCAAGCTTTGTTAAAAACAGTACAATTCTGTGATGCCATGCGAAATGCTGGT   14036
Alpha_737204        CGTGTACGCCAAGCTTTGTTAAAAACAGTACAATTCTGTGATGCCATGCGAAATGCTGGT   14031
Delta_2183060       CGTGTACGCCAAGCTTTGTTAAAAACAGTACAATTCTGTGATGCCATGCGAAATGCTGGT   14029
Omicron_7869197     CGTGTACGCCAAGCTTTGTTAAAAACAGTACAATTCTGTGATGCCATGCGAAATGCTGGT   14026
                    ************************************************************

w.t._745046         ATTGTTGGTGTACTGACATTAGATAATCAAGATCTCAATGGTAACTGGTATGATTTCGGT   14096
Alpha_737204        ATTGTTGGTGTACTGACATTAGATAATCAAGATCTCAATGGTAACTGGTATGATTTCGGT   14091
Delta_2183060       ATTGTTGGTGTACTGACATTAGATAATCAAGATCTCAATGGTAACTGGTATGATTTCGGT   14089
Omicron_7869197     ATTGTTGGTGTACTGACATTAGATAATCAAGATCTCAATGGTAACTGGTATGATTTCGGT   14086
                    ************************************************************

w.t._745046         GATTTCATACAAACCACGCCAGGTAGTGGAGTTCCTGTTGTAGATTCTTATTATTCATTG   14156
Alpha_737204        GATTTCATACAAACCACGCCAGGTAGTGGAGTTCCTGTTGTAGATTCTTATTATTCATTG   14151
Delta_2183060       GATTTCATACAAACCACGCCAGGTAGTGGAGTTCCTGTTGTAGATTCTTATTATTCATTG   14149
Omicron_7869197     GATTTCATACAAACCACGCCAGGTAGTGGAGTTCCTGTTGTAGATTCTTATTATTCATTG   14146
                    ************************************************************

w.t._745046         TTAATGCCTATATTAACCTTGACCAGGGCTTTAACTGCAGAGTCACATGTTGACACTGAC   14216
Alpha_737204        TTAATGCCTATATTAACCTTGACCAGGGCTTTAACTGCAGAGTCACATGTTGACACTGAC   14211
Delta_2183060       TTAATGCCTATATTAACCTTGACCAGGGCTTTAACTGCAGAGTCACATGTTGACACTGAC   14209
Omicron_7869197     TTAATGCCTATATTAACCTTGACCAGGGCTTTAACTGCAGAGTCACATGTTGACACTGAC   14206
                    ************************************************************

w.t._745046         TTAACAAAGCCTTACATTAAGTGGGATTTGTTAAAATATGACCTCACGGAAGAGAGGTTA   14276
Alpha_737204        TTAACAAAGCCTTACATTAAGTGGGATTTGTTAAAATATGACCTCACGGAAGAGAGGTTA   14271
Delta_2183060       TTAACAAAGCCTTACATTAAGTGGGATTTGTTAAAATATGACCTCACGGAAGAGAGGTTA   14269
Omicron_7869197     TTAACAAAGCCTTACATTAAGTGGGATTTGTTAAAATATGACCTCACGGAAGAGAGGTTA   14266
                    ************************************************************

w.t._745046         AAACTCTTTGACCGTTATTTTAAATATTGGGATCAGACATATCACCCAAATTGTGTTAAC   14336
Alpha_737204        AAACTCTTTGACCGTTATTTTAAATATTGGGATCAGACATACCACCCAAATTGTGTTAAC   14331
Delta_2183060       AAACTCTTTGACCGTTATTTTAAATATTGGGATCAGACATACCACCCAAATTGTGTTAAC   14329
Omicron_7869197     AAACTCTTTGACCGTTATTTTAAATATTGGGATCAGACATACCACCCAAATTGTGTTAAC   14326
                    ***************************************** ******************

w.t._745046         TGTTTGGATGACAGATGCATTCTGCATTGTGCAAACTTTAATGTTTTATTCTCTACAGTG   14396
Alpha_737204        TGTTTGGATGACAGATGCATTCTGCATTGTGCAAACTTTAATGTTTTATTCTCTACAGTG   14391
Delta_2183060       TGTTTGGATGACAGATGCATTCTGCATTGTGCAAACTTTAATGTTTTATTCTCTACAGTG   14389
Omicron_7869197     TGTTTGGATGACAGATGCATTCTGCATTGTGCAAACTTTAATGTTTTATTCTCTACAGTG   14386
                    ************************************************************
```

```
w.t._745046         TTCCCACTTACAAGTTTTGGACCACTAGTGAGAAAAATATTTGTTGATGGTGTTCCATTT   14456
Alpha_737204        TTCCCACTTACAAGTTTTGGACCACTAGTGAGAAAAATATTTGTTGATGGTGTTCCATTT   14451
Delta_2183060       TTCCCACTTACAAGTTTTGGACCACTAGTGAGAAAAATATTTGTTGATGGTGTTCCATTT   14449
Omicron_7869197     TTCCCACTTACAAGTTTTGGACCACTAGTGAGAAAAATATTTGTTGATGGTGTTCCATTT   14446
                    ************************************************************

w.t._745046         GTAGTTTCAACTGGATACCACCTCAGAGAGCTAGGTGTTGTACATAATCAGGATGTAAAC   14516
Alpha_737204        GTAGTTTCAACTGGATACCACCTCAGAGAGCTAGGTGTTGTACATAATCAGGATGTAAAC   14511
Delta_2183060       GTAGTTTCAACTGGATACCACCTCAGAGAGCTAGGTGTTGTACATAATCAGGATGTAAAC   14509
Omicron_7869197     GTAGTTTCAACTGGATACCACCTCAGAGAGCTAGGTGTTGTACATAATCAGGATGTAAAC   14506
                    ************************************************************

w.t._745046         TTACATAGCTCTAGACTTAGTTTTAAGGAATTACTTGTGTATGCTGCTGACCCCTCTATG   14576
Alpha_737204        TTACATAGCTCTAGACTTAGTTTTAAGGAATTACTTGTGTATGCTGCTGACCCTGCTATG   14571
Delta_2183060       TTACATAGCTCTAGACTTAGTTTTAAGGAATTACTTGTGTATGCTGCTGACCCTGCTATG   14569
Omicron_7869197     TTACATAGCTCTAGACTTAGTTTTAAGGAATTACTTGTGTATGCTGCTGACCCTGCTATG   14566
                    ****************************************************** *****

w.t._745046         CACGCTGCCTCTGGTAATCTATTACTAGATAAACGCACTACGTGCTTTTCAGTAGCTGCA   14636
Alpha_737204        CACGCTGCCTCTGGTAATCTATTACTAGATAAACGCACTACGTGCTTTTCAGTAGCTGCA   14631
Delta_2183060       CACGCTGCCTCTGGTAATCTATTACTAGATAAACGCACTACGTGCTTTTCAGTAGCTGCA   14629
Omicron_7869197     CACGCTGCCTCTGGTAATCTATTACTAGATAAACGCACTACGTGCTTTTCAGTAGCTGCA   14626
                    ************************************************************

w.t._745046         CTTACTAACAATGTTGCTTTTCAAACTGTCAAACCCGGTAATTTTAACAAAGACCTCTAT   14696
Alpha_737204        CTTACTAACAATGTTGCTTTTCAAACTGTCAAACCTGGTAATTTTAACAAAGACCTCTAT   14691
Delta_2183060       CTTACTAACAATGTTGCTTTTCAAACTGTCAAACCCGGTAATTTTAACAAAGACCTCTAT   14689
Omicron_7869197     CTTACTAACAATGTTGCTTTTCAAACTGTCAAACCCGGTAATTTTAACAAAGACCTCTAT   14686
                    *********************************** ************************

w.t._745046         GACTTTGCTGTGTCTAAGGGTTTCTTTAAGGAAGGAAGTTCTGTTGAATTAAAACACCTC   14756
Alpha_737204        GACTTTGCTGTGTCTAAGGGTTTCTTTAAGGAAGGAAGTTCTGTTGAATTAAAACACCTC   14751
Delta_2183060       GACTTTGCTGTGTCTAAGGGTTTCTTTAAGGAAGGAAGTTCTGTTGAATTAAAACACCTC   14749
Omicron_7869197     GACTTTGCTGTGTCTAAGGGTTTCTTTAAGGAAGGAAGTTCTGTTGAATTAAAACACCTC   14746
                    ************************************************************

w.t._745046         TTCTTTGCTCAGGATGGTAATGCTGCTATCAGCGATTATGACTACTATCGTTATAATCTA   14816
Alpha_737204        TTCTTTGCTCAGGATGGTAATGCTGCTATCAGCGATTATGACTACTATCGTTATAATCTA   14811
Delta_2183060       TTCTTTGCTCAGGATGGTAATGCTGCTATCAGCGATTATGACTACTATCGTTATAATCTA   14809
Omicron_7869197     TTCTTTGCTCAGGATGGTAATGCTGCTATCAGCGATTATGACTACTATCGTTATAATCTA   14806
                    ************************************************************

w.t._745046         CCAACAATGTGTGATATCAGACAACTACTATTTGTAGTTGAAGTTGTTGATAAGTACTTT   14876
Alpha_737204        CCAACAATGTGTGATATCAGACAACTACTATTTGTAGTTGAAGTTGTTGATAAGTACTTT   14871
Delta_2183060       CCAACAATGTGTGATATCAGACAACTACTATTTGTAGTTGAAGTTGTTGATAAGTACTTT   14869
Omicron_7869197     CCAACAATGTGTGATATCAGACAACTACTATTTGTAGTTGAAGTTGTTGATAAGTACTTT   14866
                    ************************************************************

w.t._745046         GATTGTTACGATGGTGGCTGTATTAATGCTAACCAAGTCATCGTCAACAACCTAGACAAA   14936
Alpha_737204        GATTGTTACGATGGTGGCTGTATTAATGCTAACCAAGTCATCGTCAACAACCTAGACAAA   14931
Delta_2183060       GATTGTTACGATGGTGGCTGTATTAATGCTAACCAAGTCATCGTCAACAACCTAGACAAA   14929
Omicron_7869197     GATTGTTACGATGGTGGCTGTATTAATGCTAACCAAGTCATCGTCAACAACCTAGACAAA   14926
                    ************************************************************

w.t._745046         TCAGCTGGTTTTCCATTTAATAAATGGGGTAAGGCTAGACTTTATTATGATTCAATGAGT   14996
Alpha_737204        TCAGCTGGTTTTCCATTTAATAAATGGGGTAAGGCTAGACTTTATTATGATTCAATGAGT   14991
Delta_2183060       TCAGCTGGTTTTCCATTTAATAAATGGGGTAAGGCTAGACTTTATTATGATTCAATGAGT   14989
Omicron_7869197     TCAGCTGGTTTTCCATTTAATAAATGGGGTAAGGCTAGACTTTATTATGATTCAATGAGT   14986
                    ************************************************************
```

```
w.t._745046         TATGAGGATCAAGATGCACTTTTCGCATATACAAAACGTAATGTCATCCCTACTATAACT   15056
Alpha_737204        TATGAGGATCAAGATGCACTTTTCGCATATACAAAACGTAATGTCATCCCTACTATAACT   15051
Delta_2183060       TATGAGGATCAAGATGCACTTTTCGCATATACAAAACGTAATGTCATCCCTACTATAACT   15049
Omicron_7869197     TATGAGGATCAAGATGCACTTTTCGCATATACAAAACGTAATGTCATCCCTACTATAACT   15046
                    ************************************************************

w.t._745046         CAAATGAATCTTAAGTATGCCATTAGTGCAAAGAATAGAGCTCGCACCGTAGCTGGTGTC   15116
Alpha_737204        CAAATGAATCTTAAGTATGCCATTAGTGCAAAGAATAGAGCTCGCACCGTAGCTGGTGTC   15111
Delta_2183060       CAAATGAATCTTAAGTATGCCATTAGTGCAAAGAATAGAGCTCGCACCGTAGCTGGTGTC   15109
Omicron_7869197     CAAATGAATCTTAAGTATGCCATTAGTGCAAAGAATAGAGCTCGCACCGTAGCTGGTGTC   15106
                    ************************************************************

w.t._745046         TCTATCTGTAGTACTATGACCAATAGACAGTTTCATCAAAAATTATTGAAATCAATAGCC   15176
Alpha_737204        TCTATCTGTAGTACTATGACCAATAGACAGTTTCATCAAAAATTATTGAAATCAATAGCC   15171
Delta_2183060       TCTATCTGTAGTACTATGACCAATAGACAGTTTCATCAAAAATTATTGAAATCAATAGCC   15169
Omicron_7869197     TCTATCTGTAGTACTATGACCAATAGACAGTTTCATCAAAAATTATTGAAATCAATAGCC   15166
                    ************************************************************

w.t._745046         GCCACTAGAGGAGCTACTGTAGTAATTGGAACAAGCAAATTCTATGGTGGTTGGCACAAC   15236
Alpha_737204        GCCACTAGAGGAGCTACTGTAGTAATTGGAACAAGCAAATTCTATGGTGGTTGGCACAAC   15231
Delta_2183060       GCCACTAGAGGAGCTACTGTAGTAATTGGAACAAGCAAATTCTATGGTGGTTGGCACAAC   15229
Omicron_7869197     GCCACTAGAGGAGCTACTGTAGTAATTGGAACAAGCAAATTCTATGGTGGTTGGCACAAT   15226
                    ***********************************************************

w.t._745046         ATGTTAAAAACTGTTTATAGTGATGTAGAAAACCCTCACCTTATGGGTTGGGATTATCCT   15296
Alpha_737204        ATGTTAAAAACTGTTTATAGTGATGTAGAAAACCCTCATCTTATGGGTTGGGATTATCCT   15291
Delta_2183060       ATGTTAAAAACTGTTTATAGTGATGTAGAAAACCCTCACCTTATGGGTTGGGATTATCCT   15289
Omicron_7869197     ATGTTAAAAACTGTTTATAGTGATGTAGAAAACCCTCACCTTATGGGTTGGGATTATCCT   15286
                    ************************************** *********************

w.t._745046         AAATGTGATAGAGCCATGCCTAACATGCTTAGAATTATGGCCTCACTTGTTCTTGCTCGC   15356
Alpha_737204        AAATGTGATAGAGCCATGCCTAACATGCTTAGAATTATGGCCTCACTTGTTCTTGCTCGC   15351
Delta_2183060       AAATGTGATAGAGCCATGCCTAACATGCTTAGAATTATGGCCTCACTTGTTCTTGCTCGC   15349
Omicron_7869197     AAATGTGATAGAGCCATGCCTAACATGCTTAGAATTATGGCCTCACTTGTTCTTGCTCGC   15346
                    ************************************************************

w.t._745046         AAACATACAACGTGTTGTAGCTTGTCACACCGTTTCTATAGATTAGCTAATGAGTGTGCT   15416
Alpha_737204        AAACATACAACGTGTTGTAGCTTGTCACACCGTTTCTATAGATTAGCTAATGAGTGTGCT   15411
Delta_2183060       AAACATACAACGTGTTGTAGCTTGTCACACCGTTTCTATAGATTAGCTAATGAGTGTGCT   15409
Omicron_7869197     AAACATACAACGTGTTGTAGCTTGTCACACCGTTTCTATAGATTAGCTAATGAGTGTGCT   15406
                    ************************************************************

w.t._745046         CAAGTATTGAGTGAAATTGTCATGTGTGGCGGTTCACTATATGTTAAACCAGGTGGAACC   15476
Alpha_737204        CAAGTATTGAGTGAAATGGTCATGTGTGGCGGTTCACTATATGTTAAACCAGGTGGAACC   15471
Delta_2183060       CAAGTATTGAGTGAAATGGTCATGTGTGGCAGTTCACTATATGTTAAACCAGGTGGAACC   15469
Omicron_7869197     CAAGTATTGAGTGAAATGGTCATGTGTGGCGGTTCACTATATGTTAAACCAGGTGGAACC   15466
                    ***************** ************ *****************************

w.t._745046         TCATCAGGAGATGCCACAACTGCTTATGCTAATAGTGTTTTTAACATTTGTCAAGCTGTC   15536
Alpha_737204        TCATCAGGAGATGCCACAACTGCTTATGCTAATAGTGTTTTTAACATTTGTCAAGCTGTC   15531
Delta_2183060       TCATCAGGAGATGCCACAACTGCTTATGCTAATAGTGTTTTTAACATTTGTCAAGCTGTC   15529
Omicron_7869197     TCATCAGGAGATGCCACAACTGCTTATGCTAATAGTGTTTTTAACATTTGTCAAGCTGTC   15526
                    ************************************************************

w.t._745046         ACGGCCAATGTTAATGCACTTTTATCTACTGATGGTAACAAAATTGCCGATAAGTATGTC   15596
Alpha_737204        ACGGCCAATGTTAATGCACTTTTATCTACTGATGGTAACAAAATTGCCGATAAGTATGTC   15591
Delta_2183060       ACGGCCAATGTTAATGCACTTTTATCTACTGATGGTAACAAAATTGCCGATAAGTATGTC   15589
Omicron_7869197     ACGGCCAATGTTAATGCACTTTTATCTACTGATGGTAACAAAATTGCCGATAAGTATGTC   15586
                    ************************************************************
```

```
w.t._745046         CGCAATTTACAACACAGACTTTATGAGTGTCTCTATAGAAATAGAGATGTTGACACAGAC   15656
Alpha_737204        CGCAATTTACAACACAGACTTTATGAGTGTCTCTATAGAAATAGAGATGTTGACACAGAC   15651
Delta_2183060       CGCAATTTACAACACAGACTTTATGAGTGTCTCTATAGAAATAGAGATGTTGACACAGAC   15649
Omicron_7869197     CGCAATTTACAACACAGACTTTATGAGTGTCTCTATAGAAATAGAGATGTTGACACAGAC   15646
                    ************************************************************

w.t._745046         TTTGTGAATGAGTTTTACGCATATTTGCGTAAACATTTCTCAATGATGATACTCTCTGAC   15716
Alpha_737204        TTTGTGAATGAGTTTTACGCATATTTGCGTAAACATTTCTCAATGATGATACTCTCTGAC   15711
Delta_2183060       TTTGTGAATGAGTTTTACGCATATTTGCGTAAACATTTCTCAATGATGATACTCTCTGAC   15709
Omicron_7869197     TTTGTGAATGAGTTTTACGCATATTTGCGTAAACATTTCTCAATGATGATACTCTCTGAC   15706
                    ************************************************************

w.t._745046         GATGCTGTTGTGTGTTTCAATAGCACTTATGCATCTCAAGGTCTAGTGGCTAGCATAAAG   15776
Alpha_737204        GATGCTGTTGTGTGTTTCAATAGCACTTATGCATCTCAAGGTCTAGTGGCTAGCATAAAG   15771
Delta_2183060       GATGCTGTTGTGTGTTTCAATAGCACTTATGCATCTCAAGGTCTAGTGGCTAGCATAAAG   15769
Omicron_7869197     GATGCTGTTGTGTGTTTCAATAGCACTTATGCATCTCAAGGTCTAGTGGCTAGCATAAAG   15766
                    ************************************************************

w.t._745046         AACTTTAAGTCAGTTCTTTATTATCAAAACAATGTTTTTATGTCTGAAGCAAAATGTTGG   15836
Alpha_737204        AACTTTAAGTCAGTTCTTTATTATCAAAACAATGTTTTTATGTCTGAAGCAAAATGTTGG   15831
Delta_2183060       AACTTTAAGTCAGTTCTTTATTATCAAAACAATGTTTTTATGTCTGAAGCAAAATGTTGG   15829
Omicron_7869197     AACTTTAAGTCAGTTCTTTATTATCAAAACAATGTTTTTATGTCTGAAGCAAAATGTTGG   15826
                    ************************************************************

w.t._745046         ACTGAGACTGACCTTACTAAAGGACCTCATGAATTTTGCTCTCAACATACAATGCTAGTT   15896
Alpha_737204        ACTGAGACTGACCTTACTAAAGGACCTCATGAATTTTGCTCTCAACATACAATGCTAGTT   15891
Delta_2183060       ACTGAGACTGACCTTACTAAAGGACCTCATGAATTTTGCTCTCAACATACAATGCTAGTT   15889
Omicron_7869197     ACTGAGACTGACCTTACTAAAGGACCTCATGAATTTTGCTCTCAACATACAATGCTAGTT   15886
                    ************************************************************

w.t._745046         AAACAGGGTGATGATTATGTGTACCCTCCTTACCCAGATCCATCAAGAATCCTAGGGGCC   15956
Alpha_737204        AAACAGGGTGATGATTATGTGTACCCTCCTTACCCAGATCCATCAAGAATCCTAGGGGCC   15951
Delta_2183060       AAACAGGGTGATGATTATGTGTACCCTCCTTACCCAGATCCATCAAGAATCCTAGGGGCC   15949
Omicron_7869197     AAACAGGGTGATGATTATGTGTACCCTCCTTACCCAGATCCATCAAGAATCCTAGGGGCC   15946
                    ************************************************************

w.t._745046         GGCTGTTTTGTAGATGATATCGTAAAAACAGATGGTACACTTATGATTGAACGGTTCGTG   16016
Alpha_737204        GGCTGTTTTGTAGATGATATCGTAAAAACAGATGGTACACTTATGATTGAACGGTTCGTG   16011
Delta_2183060       GGCTGTTTTGTAGATGATATCGTAAAAACAGATGGTACACTTATGATTGAACGGTTCGTG   16009
Omicron_7869197     GGCTGTTTTGTAGATGATATCGTAAAAACAGATGGTACACTTATGATTGAACGGTTCGTG   16006
                    ************************************************************

w.t._745046         TCTTTAGCTATAGATGCTTACCCACTTACTAAACATCCTAATCAGGAGTATGCTGATGTC   16076
Alpha_737204        TCTTTAGCTATAGATGCTTACCCACTTACTAAACATCCTAATCAGGAGTATGCTGATGTC   16071
Delta_2183060       TCTTTAGCTATAGATGCTTACCCACTTACTAAACATCCTAATCAGGAGTATGCTGATGTC   16069
Omicron_7869197     TCTTTAGCTATAGATGCTTACCCACTTACTAAACATCCTAATCAGGAGTATGCTGATGTC   16066
                    ************************************************************

w.t._745046         TTTCATTTGTACTTACAATACATAAGAAAGCTACATGATGAGTTAACAGGACACATGTTA   16136
Alpha_737204        TTTCATTTGTACTTACAATACATAAGAAAGCTACATGATGAGTTAACAGGACACATGTTA   16131
Delta_2183060       TTTCATTTGTACTTACAATACATAAGAAAGCTACATGATGAGTTAACAGGACACATGTTA   16129
Omicron_7869197     TTTCATTTGTACTTACAATACATAAGAAAGCTACATGATGAGTTAACAGGACACATGTTA   16126
                    ************************************************************

w.t._745046         GACATGTATTCTGTTATGCTTACTAATGATAACACCTCAAGGTATTGGGAACCTGAGTTT   16196
Alpha_737204        GACATGTATTCTGTTATGCTTACTAATGATAACACCTCAAGGTATTGGGAACCTGAGTTT   16191
Delta_2183060       GACATGTATTCTGTTATGCTTACTAATGATAACACCTCAAGGTATTGGGAACCTGAGTTT   16189
Omicron_7869197     GACATGTATTCTGTTATGCTTACTAATGATAACACCTCAAGGTATTGGGAACCTGAGTTT   16186
                    *********************************** ************************
```

```
w.t._745046         TATGAGGCTATGTACACACCGCATACAGTCTTACAGGCTGTTGGGGCTTGTGTTCTTTGC   16256
Alpha_737204        TATGAGGCTATGTACACACCGCATACAGTCTTACAGGCTGTTGGGGCTTGTGTTCTTTGC   16251
Delta_2183060       TATGAGGCTATGTACACACCGCATACAGTCTTACAGGCTGTTGGGGCTTGTGTTCTTTGC   16249
Omicron_7869197     TATGAGGCTATGTACACACCGCATACAGTCTTACAGGCTGTTGGGGCTTGTGTTCTTTGC   16246
                    ************************************************************

w.t._745046         AATTCACAGACCTCATTAAGATGTGGTGCTTGCATACGTAGACCATTCTTATGTTGTAAA   16316
Alpha_737204        AATTCACAGACCTCATTAAGATGTGGTGCTTGCATACGTAGACCATTCTTATGTTGTAAA   16311
Delta_2183060       AATTCACAGACCTCATTAAGATGTGGTGCTTGCATACGTAGACCATTCTTATGTTGTAAA   16309
Omicron_7869197     AATTCACAGACCTCATTAAGATGTGGTGCTTGCATACGTAGACCATTCTTATGTTGTAAA   16306
                    ************************************************************

w.t._745046         TGCTGTTACGACCATGTCATATCAACATCACATAAATTAGTCTTGTCTGTTAATCCGTAT   16376
Alpha_737204        TGCTGTTACGACCATGTCATATCAACATCACATAAATTAGTCTTGTCTGTTAATCCGTAT   16371
Delta_2183060       TGCTGTTACGACCATGTCATATCAACATCACATAAATTAGTCTTGTCTGTTAATCCGTAT   16369
Omicron_7869197     TGCTGTTACGACCATGTCATATCAACATCACATAAATTAGTCTTGTCTGTTAATCCGTAT   16366
                    ************************************************************

w.t._745046         GTTTGCAATGCTCCAGGTTGTGATGTCACAGATGTGACTCAACTTTACTTAGGAGGTATG   16436
Alpha_737204        GTTTGCAATGCTCCAGGTTGTGATGTCACAGATGTGACTCAACTTTACTTAGGAGGTATG   16431
Delta_2183060       GTTTGCAATGCTCCAGGTTGTGATGTCACAGATGTGACTCAACTTTACTTAGGAGGTATG   16429
Omicron_7869197     GTTTGCAATGCTCCAGGTTGTGATGTCACAGATGTGACTCAACTTTACTTAGGAGGTATG   16426
                    ************************************************************

w.t._745046         AGCTATTATTGTAAATCACATAAACCACCCATTAGTTTTCCATTGTGTGCTAATGGACAA   16496
Alpha_737204        AGCTATTATTGTAAATCACATAAACCACCCATTAGTTTTCCATTGTGTGCTAATGGACAA   16491
Delta_2183060       AGCTATTATTGTAAATCACATAAACTACCCATTAGTTTTCCATTGTGTGCTAATGGACAA   16489
Omicron_7869197     AGCTATTATTGTAAATCACATAAACCACCCATTAGTTTTCCATTGTGTGCTAATGGACAA   16486
                    ************************* **********************************

w.t._745046         GTTTTTGGTTTATATAAAAATACATGTGTTGGTAGCGATAATGTTACTGACTTTAATGCA   16556
Alpha_737204        GTTTTTGGTTTATATAAAAATACATGTGTTGGTAGCGATAATGTTACTGACTTTAATGCA   16551
Delta_2183060       GTTTTTGGTTTATATAAAAATACATGTGTTGGTAGCGATAATGTTACTGACTTTAATGCA   16549
Omicron_7869197     GTTTTTGGTTTATATAAAAATACATGTGTTGGTAGCGATAATGTTACTGACTTTAATGCA   16546
                    ************************************************************

w.t._745046         ATTGCAACATGTGACTGGACAAATGCTGGTGATTACATTTTAGCTAACACCTGTACTGAA   16616
Alpha_737204        ATTGCAACATGTGACTGGACAAATGCTGGTGATTACATTTTAGCTAACACCTGTACTGAA   16611
Delta_2183060       ATTGCAACATGTGACTGGACAAATGCTGGTGATTACATTTTAGCTAACACCTGTACTGAA   16609
Omicron_7869197     ATTGCAACATGTGACTGGACAAATGCTGGTGATTACATTTTAGCTAACACCTGTACTGAA   16606
                    ************************************************************

w.t._745046         AGACTCAAGCTTTTTGCAGCAGAAACGCTCAAAGCTACTGAGGAGACATTTAAACTGTCT   16676
Alpha_737204        AGACTCAAGCTTTTTGCAGCAGAAACGCTCAAAGCTACTGAGGAGACATTTAAACTGTCT   16671
Delta_2183060       AGACTCAAGCTTTTTGCAGCAGAAACGCTCAAAGCTACTGAGGAGACATTTAAACTGTCT   16669
Omicron_7869197     AGACTCAAGCTTTTTGCAGCAGAAACGCTCAAAGCTACTGAGGAGACATTTAAACTGTCT   16666
                    ************************************************************

w.t._745046         TATGGTATTGCTACTGTACGTGAAGTGCTGTCTGACAGAGAATTACATCTTTCATGGGAA   16736
Alpha_737204        TATGGTATTGCTACTGTACGTGAAGTGCTGTCTGACAGAGAATTACATCTTTCATGGGAA   16731
Delta_2183060       TATGGTATTGCTACTGTACGTGAAGTGCTGTCTGACAGAGAATTACATCTTTCATGGGAA   16729
Omicron_7869197     TATGGTATTGCTACTGTACGTGAAGTGCTGTCTGACAGAGAATTACATCTTTCATGGGAA   16726
                    ************************************************************

w.t._745046         GTTGGTAAACCTAGACCACCACTTAACCGAAATTATGTCTTTACTGGTTATCGTGTAACT   16796
Alpha_737204        GTTGGTAAACCTAGACCACCACTTAACCGAAATTATGTCTTTACTGGTTATCGTGTAACT   16791
Delta_2183060       GTTGGTAAACCTAGACCACCACTTAACCGAAATTATGTCTTTACTGGTTATCGTGTAACT   16789
Omicron_7869197     GTTGGTAAACCTAGACCACCACTTAACCGAAATTATGTCTTTACTGGTTATCGTGTAACT   16786
                    ************************************************************
```

```
w.t._745046        AAAAACAGTAAAGTACAAATAGGAGAGTACACCTTTGAAAAAGGTGACTATGGTGATGCT   16856
Alpha_737204       AAAAACAGTAAAGTACAAATAGGAGAGTACACCTTTGAAAAAGGTGACTATGGTGATGCT   16851
Delta_2183060      AAAAACAGTAAAGTACAAATAGGAGAGTACACCTTTGAAAAAGGTGACTATGGTGATGCT   16849
Omicron_7869197    AAAAACAGTAAAGTACAAATAGGAGAGTACACCTTTGAAAAAGGTGACTATGGTGATGCT   16846
                   ************************************************************

w.t._745046        GTTGTTTACCGAGGTATAACAACTTACAAATTAAATGTTGGTGATTATTTTGTGCTGACA   16916
Alpha_737204       GTTGTTTACCGAGGTACAACAACTTACAAATTAAATGTTGGTGATTATTTTGTGCTGACA   16911
Delta_2183060      GTTGTTTACCGAGGTACAACAACTTACAAATTAAATGTTGGTGATTATTTTGTGCTGACA   16909
Omicron_7869197    GTTGTTTACCGAGGTACAACAACTTACAAATTAAATGTTGGTGATTATTTTGTGCTGACA   16906
                   **************** *******************************************

w.t._745046        TCACATACAGTAATGCCATTAAGTGCACCTACACTAGTGCCACAAGAGCACTATGTTAGA   16976
Alpha_737204       TCACATACAGTAATGCCATTAAGTGCACCTACACTAGTGCCACAAGAGCACTATGTTAGA   16971
Delta_2183060      TCACATACAGTAATGCCATTAAGTGCACCTACACTAGTGCCACAAGAGCACTATGTTAGA   16969
Omicron_7869197    TCACATACAGTAATGCCATTAAGTGCACCTACACTAGTGCCACAAGAGCACTATGTTAGA   16966
                   ************************************************************

w.t._745046        ATTACTGGCTTATACCCAACACTCAATATCTCAGATGAGTTTTCTAGCAATGTTGCAAAT   17036
Alpha_737204       ATTACTGGCTTATACCCAACACTCAATATCTCAGATGAGTTTTCTAGCAATGTTGCAAAT   17031
Delta_2183060      ATTACTGGCTTATACCCAACACTCAATATCTCAGATGAGTTTTCTAGCAATGTTGCAAAT   17029
Omicron_7869197    ATTACTGGCTTATACCCAACACTCAATATCTCAGATGAGTTTTCTAGCAATGTTGCAAAT   17026
                   ************************************************************

w.t._745046        TATCAAAAGGTTGGTATGCAAAAGTACTCTACACTCCAGGGACCACCTGGTACTGGTAAG   17096
Alpha_737204       TATCAAAAGGTTGGTATGCAAAAGTATTCTACACTCCAGGGACCACCTGGTACTGGTAAG   17091
Delta_2183060      TATCAAAAGGTTGGTATGCAAAAGTATTCTACACTCCAGGGACCACCTGGTACTGGTAAG   17089
Omicron_7869197    TATCAAAAGGTTGGTATGCAAAAGTATTCTACACTCCAGGGACCACCTGGTACTGGTAAG   17086
                   ************************** *********************************

w.t._745046        AGTCATTTTGCTATTGGCCTAGCTCTCTACTACCCCTCTGCTCGCATAGTGTATACAGCT   17156
Alpha_737204       AGTCATTTTGCTATTGGCCTAGCTCTCTACTACCCCTCTGCTCGCATAGTGTATACAGCT   17151
Delta_2183060      AGTCATTTTGCTATTGGCCTAGCTCTCTACTACCCCTCTGCTCGCATAGTGTATACAGCT   17149
Omicron_7869197    AGTCATTTTGCTATTGGCCTAGCTCTCTACTACCCCTCTGCTCGCATAGTGTATACAGCT   17146
                   ************************************************************

w.t._745046        TGCTCTCATGCCGCTGTTGATGCACTATGTGAGAAGGCATTAAAATATTTGCCTATAGAT   17216
Alpha_737204       TGCTCTCATGCCGCTGTTGATGCACTATGTGAGAAGGCATTAAAATATTTGCCTATAGAT   17211
Delta_2183060      TGCTCTCATGCCGCTGTTGATGCACTATGTGAGAAGGCATTAAAATATTTGCCTATAGAT   17209
Omicron_7869197    TGCTCTCATGCCGCTGTTGATGCACTATGTGAGAAGGCATTAAAATATTTGCCTATAGAT   17206
                   ************************************************************

w.t._745046        AAATGTAGTAGAATTATACCTGCACGTGCTCGTGTAGAGTGTTTTGATAAATTCAAAGTG   17276
Alpha_737204       AAATGTAGTAGAATTATACCTGCACGTGCTCGTGTAGAGTGTTTTGATAAATTCAAAGTG   17271
Delta_2183060      AAATGTAGTAGAATTATACCTGCACGTGCTCGTGTAGAGTGTTTTGATAAATTCAAAGTG   17269
Omicron_7869197    AAATGTAGTAGAATTATACCTGCACGTGCTCGTGTAGAGTGTTTTGATAAATTCAAAGTG   17266
                   ************************************************************

w.t._745046        AATTCAACATTAGAACAGTATGTCTTTTGTACTGTAAATGCATTGCCTGAGACGACAGCA   17336
Alpha_737204       AATTCAACATTAGAACAGTATGTCTTTTGTACTGTAAATGCATTGCCTGAGACGACAGCA   17331
Delta_2183060      AATTCAACATTAGAACAGTATGTCTTTTGTACTGTAAATGCATTGCCTGAGACGACAGCA   17329
Omicron_7869197    AATTCAACATTAGAACAGTATGTCTTTTGTACTGTAAATGCATTGCCTGAGACGACAGCA   17326
                   ************************************************************

w.t._745046        GATATAGTTGTCTTTGATGAAATTTCAATGGCCACAAATTATGATTTGAGTGTTGTCAAT   17396
Alpha_737204       GATATAGTTGTCTTTGATGAAATTTCAATGGCCACAAATTATGATTTGAGTGTTGTCAAT   17391
Delta_2183060      GATATAGTTGTCTTTGATGAAATTTCAATGGCCACAAATTATGATTTGAGTGTTGTCAAT   17389
Omicron_7869197    GATATAGTTGTCTTTGATGAAATTTCAATGGCCACAAATTATGATTTGAGTGTTGTCAAT   17386
                   ************************************************************
```

```
w.t._745046        GCCAGATTACGTGCTAAGCACTATGTGTACATTGGCGACCCTGCTCAATTACCTGCACCA   17456
Alpha_737204       GCCAGATTACGTGCTAAGCACTATGTGTACATTGGCGACCCTGCTCAATTACCTGCACCA   17451
Delta_2183060      GCCAGATTACGTGCTAAGCACTATGTGTACATTGGCGACCCTGCTCAATTACCTGCACCA   17449
Omicron_7869197    GCCAGATTACGTGCTAAGCACTATGTGTACATTGGCGACCCTGCTCAATTACCTGCACCA   17446
                   ************************************************************

w.t._745046        CGCACATTGCTAACTAAGGGCACACTAGAACCAGAATATTTCAATTCAGTGTGTAGACTT   17516
Alpha_737204       CGCACATTGCTAACTAAGGGCACACTAGAACCAGAATATTTCAATTCAGTGTGTAGACTT   17511
Delta_2183060      CGCACATTGCTAACTAAGGGCACACTAGAACCAGAATATTTCAATTCAGTGTGTAGACTT   17509
Omicron_7869197    CGCACATTGCTAACTAAGGGCACACTAGAACCAGAATATTTCAATTCAGTGTGTAGACTT   17506
                   ************************************************************

w.t._745046        ATGAAAACTATAGGTCCAGACATGTTCCTCGGAACTTGTCGGCGTTGTCCTGCTGAAATT   17576
Alpha_737204       ATGAAAACTATAGGTCCAGACATGTTCCTCGGAACTTGTCGGCGTTGTCCTGCTGAAATT   17571
Delta_2183060      ATGAAAACTATAGGTCCAGACATGTTCCTCGGAACTTGTCGGCGTTGTCCTGCTGAAATT   17569
Omicron_7869197    ATGAAAACTATAGGTCCAGACATGTTCCTCGGAACTTGTCGGCGTTGTCCTGCTGAAATT   17566
                   ************************************************************

w.t._745046        GTTGACACTGTGAGTGCTTTGGTTTATGATAATAAGCTTAAAGCACATAAAGACAAATCA   17636
Alpha_737204       GTTGACACTGTGAGTGCTTTGGTTTATGATAATAGGCTTAAAGCACATAAAGACAAATCA   17631
Delta_2183060      GTTGACACTGTGAGTGCTTTGGTTTATGATAATAAGCTTAAAGCACATAAAGACAAATCA   17629
Omicron_7869197    GTTGACACTGTGAGTGCTTTGGTTTATGATAATAAGCTTAAAGCACATAAAGACAAATCA   17626
                   ********************************** *************************

w.t._745046        GCTCAATGCTTTAAAATGTTTTATAAGGGTGTTATCACGCATGATGTTTCATCTGCAATT   17696
Alpha_737204       GCTCAATGCTTTAAAATGTTTTATAAGGGTGTTATCACGCATGATGTTTCATCTGCAATT   17691
Delta_2183060      GCTCAATGCTTTAAAATGTTTTATAAGGGTGTTATCACGCATGATGTTTCATCTGCAATT   17689
Omicron_7869197    GCTCAATGCTTTAAAATGTTTTATAAGGGTGTTATCACGCATGATGTTTCATCTGCAATT   17686
                   ************************************************************

w.t._745046        AACAGGCCACAAATAGGCGTGGTAAGAGAATTCCTTACACGTAACCCTGCTTGGAGAAAA   17756
Alpha_737204       AACAGGCCACAAATAGGCGTGGTAAGAGAATTCCTTACACGTAACCCTGCTTGGAGAAAA   17751
Delta_2183060      AACAGGCCACAAATAGGCGTGGTAAGAGAATTCCTTACACGTAACCCTGCTTGGAGAAAA   17749
Omicron_7869197    AACAGGCCACAAATAGGCGTGGTAAGAGAATTCCTTACACGTAACCCTGCTTGGAGAAAA   17746
                   ************************************************************

w.t._745046        GCTGTCTTTATTTCACCTTATAATTCACAGAATGCTGTAGCCTCAAAGATTTTGGGACTA   17816
Alpha_737204       GCTGTCTTTATTTCACCTTATAATTCACAGAATGCTGTAGCCTCAAAGATTTTGGGACTA   17811
Delta_2183060      GCTGTCTTTATTTCACCTTATAATTCACAGAATGCTGTAGCCTCAAAGATTTTGGGACTA   17809
Omicron_7869197    GCTGTCTTTATTTCACCTTATAATTCACAGAATGCTGTAGCCTCAAAGATTTTGGGACTA   17806
                   ************************************************************

w.t._745046        CCAACTCAAACTGTTGATTCATCACAGGGCTCAGAATATGACTATGTCATATTCACTCAA   17876
Alpha_737204       CCAACTCAAACTGTTGATTCATCACAGGGCTCAGAATATGACTATGTCATATTCACTCAA   17871
Delta_2183060      CCAACTCAAACTGTTGATTCATCACAGGGCTCAGAATATGACTATGTCATATTCACTCAA   17869
Omicron_7869197    CCAACTCAAACTGTTGATTCATCACAGGGCTCAGAATATGACTATGTCATATTCACTCAA   17866
                   ************************************************************

w.t._745046        ACCACTGAAACAGCTCACTCTTGTAATGTAAACAGATTTAATGTTGCTATTACCAGAGCA   17936
Alpha_737204       ACCACTGAAACAGCTCACTCTTGTAATGTAAACAGATTTAATGTTGCTATTACCAGAGCA   17931
Delta_2183060      ACCACTGAAACAGCTCACTCTTGTAATGTAAACAGATTTAATGTTGCTATTACCAGAGCA   17929
Omicron_7869197    ACCACTGAAACAGCTCACTCTTGTAATGTAAACAGATTTAATGTTGCTATTACCAGAGCA   17926
                   ************************************************************

w.t._745046        AAAGTAGGCATACTTTGCATAATGTCTGATAGAGACCTTTATGACAAGTTGCAATTTACA   17996
Alpha_737204       AAAGTAGGCATACTTTGCATAATGTCTGATAGAGACCTTTATGACAAGTTGCAATTTACA   17991
Delta_2183060      AAAGTAGGCATACTTTGCATAATGTCTGATAGAGACCTTTATGACAAGTTGCAATTTACA   17989
Omicron_7869197    AAAGTAGGCATACTTTGCATAATGTCTGATAGAGACCTTTATGACAAGTTGCAATTTACA   17986
                   ************************************************************
```

```
w.t._745046         AGTCTTGAAATTCCACGTAGGAATGTGGCAACTTTACAAGCTGAAAATGTAACAGGACTC   18056
Alpha_737204        AGTCTTGAAATTCCACGTAGGAATGTGGCAACTTTACAAGCTGAAAATGTAACAGGACTC   18051
Delta_2183060       AGTCTTGAAATTCCACGTAGGAATGTGGCAACTTTACAAGCTGAAAATGTAACAGGACTC   18049
Omicron_7869197     AGTCTTGAAATTCCACGTAGGAATGTGGCAACTTTACAAGCTGAAAATGTAACAGGACTC   18046
                    ************************************************************

w.t._745046         TTTAAAGATTGTAGTAAGGTAATCACTGGGTTACATCCTACACAGGCACCTACACACCTC   18116
Alpha_737204        TTTAAAGATTGTAGTAAGGTAATCACTGGGTTACATCCTACACAGGCACCTACACACCTC   18111
Delta_2183060       TTTAAAGATTGTAGTAAGGTAATCACTGGGTTACATCCTACACAGGCACCTACACACCTC   18109
Omicron_7869197     TTTAAAGATTGTAGTAAGGTAATCACTGGGTTACATCCTACACAGGCACCTACACACCTC   18106
                    ************************************************************

w.t._745046         AGTGTTGACACTAAATTCAAAACTGAAGGTTTATGTGTTGACATACCTGGCATACCTAAG   18176
Alpha_737204        AGTGTTGACACTAAATTCAAAACTGAAGGTTTATGTGTTGACATACCTGGCATACCTAAG   18171
Delta_2183060       AGTGTTGACACTAAATTCAAAACTGAAGGTTTATGTGTTGACATACCTGGCATACCTAAG   18169
Omicron_7869197     AGTGTTGACACTAAATTCAAAACTGAAGGTTTATGTGTTGACGTACCTGGCATACCTAAG   18166
                    ****************************************** *****************

w.t._745046         GACATGACCTATAGAAGACTCATCTCTATGATGGGTTTTAAAATGAATTATCAAGTTAAT   18236
Alpha_737204        GACATGACCTATAGAAGACTCATCTCTATGATGGGTTTTAAAATGAATTATCAAGTTAAT   18231
Delta_2183060       GACATGACCTATAGAAGACTCATCTCTATGATGGGTTTTAAAATGAATTATCAAGTTAAT   18229
Omicron_7869197     GACATGACCTATAGAAGACTCATCTCTATGATGGGTTTTAAAATGAATTATCAAGTTAAT   18226
                    ************************************************************

w.t._745046         GGTTACCCTAACATGTTTATCACCCGCGAAGAAGCTATAAGACATGTACGTGCATGGATT   18296
Alpha_737204        GGTTACCCTAACATGTTTATCACCCGCGAAGAAGCTATAAGACATGTACGTGCATGGATT   18291
Delta_2183060       GGTTACCCTAACATGTTTATCACCCGCGAAGAAGCTATAAGACATGTACGTGCATGGATT   18289
Omicron_7869197     GGTTACCCTAACATGTTTATCACCCGCGAAGAAGCTATAAGACATGTACGTGCATGGATT   18286
                    ************************************************************

w.t._745046         GGCCTCGATGTCGAGGGGTGTCATGCTACTAGAGAAGCTGTTGGTACCAATTTACCTTTA   18356
Alpha_737204        GGCCTCGATGTCGAGGGGTGTCATGCTACTAGAGAAGCTGTTGGTACCAATTTACCTTTA   18351
Delta_2183060       GGCCTCGATGTCGAGGGGTGTCATGCTACTAGAGAAGCTGTTGGTACCAATTTACCTTTA   18349
Omicron_7869197     GGCCTCGATGTCGAGGGGTGTCATGCTACTAGAGAAGCTGTTGGTACCAATTTACCTTTA   18346
                    ************************************************************

w.t._745046         CAGCTAGGTTTTTCTACAGGTGTTAACCTAGTTGCTGTACCTACAGGTTATGTTGATACA   18416
Alpha_737204        CAGCTAGGTTTTTCTACAGGTGTTAACCTAGTTGCTGTACCTACAGGTTATGTTGATACA   18411
Delta_2183060       CAGCTAGGTTTTTCTACAGGTGTTAACCTAGTTGCTGTACCTACAGGTTATGTTGATACA   18409
Omicron_7869197     CAGCTAGGTTTTTCTACAGGTGTTAACCTAGTTGCTGTACCTACAGGTTATGTTGATACA   18406
                    ************************************************************

w.t._745046         CCTAATAATACAGATTTTTCCAGAGTTAGTGCTAAACCACCGCCTGGAGATCAATTTAAA   18476
Alpha_737204        CCTAATAATACAGATTTTTCCAGAGTTAGTGCTAAACCACCGCCTGGAGATCAATTTAAA   18471
Delta_2183060       CCTAATAATACAGATTTTTCCAGAGTTAGTGCTAAACCACCGCCTGGAGATCAATTTAAA   18469
Omicron_7869197     CCTAATAATACAGATTTTTCCAGAGTTAGTGCTAAACCACCGCCTGGAGATCAATTTAAA   18466
                    ************************************************************

w.t._745046         CACCTCATACCACTTATGTACAAAGGACCTCCTTGGAATGTAGTGCGTATAAAGATTGTA   18536
Alpha_737204        CACCTCATACCACTTATGTACAAAGGACCTCCTTGGAATGTAGTGCGTATAAAGATTGTA   18531
Delta_2183060       CACCTCATACCACTTATGTACAAAGGACCTCCTTGGAATGTAGTGCGTATAAAGATTGTA   18529
Omicron_7869197     CACCTCATACCACTTATGTACAAAGGACCTCCTTGGAATGTAGTGCGTATAAAGATTGTA   18526
                    ************************************************************

w.t._745046         CAAATGTTAAGTGACACACTTAAAAATCTCTCTGACAGAGTCGTATTTGTCTTATGGGCA   18596
Alpha_737204        CAAATGTTAAGTGACACACTTAAAAATCTCTCTGACAGAGTCGTATTTGTCTTATGGGCA   18591
Delta_2183060       CAAATGTTAAGTGACACACTTAAAAATCTCTCTGACAGAGTCGTATTTGTCTTATGGGCA   18589
Omicron_7869197     CAAATGTTAAGTGACACACTTAAAAATCTCTCTGACAGAGTCGTATTTGTCTTATGGGCA   18586
                    ************************************************************
```

```
w.t._745046          CATGGCTTTGAGTTGACATCTATGAAGTATTTTGTGAAAATAGGACCTGAGCGCACCTGT   18656
Alpha_737204         CATGGCTTTGAGTTGACATCTATGAAGTATTTTGTGAAAATAGGACCTGAGCGCACCTGT   18651
Delta_2183060        CATGGCTTTGAGTTGACATCTATGAAGTATTTTGTGAAAATAGGACCTGAGCGCACCTGT   18649
Omicron_7869197      CATGGCTTTGAGTTGACATCTATGAAGTATTTTGTGAAAATAGGACCTGAGCGCACCTGT   18646
                     ************************************************************

w.t._745046          TGTCTATGTGATAGACGTGCCACATGCTTTTCCACTGCCTCAGACACTTATGCCTGTTGG   18716
Alpha_737204         TGTCTATGTGATAGACGTGCCACATGCTTTTCCACTGCCTCAGACACTTATGCCTGTTGG   18711
Delta_2183060        TGTCTATGTGATAGACGTGCCACATGCTTTTCCACTGCCTCAGACACTTATGCCTGTTGG   18709
Omicron_7869197      TGTCTATGTGATAGACGTGCCACATGCTTTTCCACTGCCTCAGACACTTATGCCTGTTGG   18706
                     ************************************************************

w.t._745046          CATCATTCTATTGGATTTGATTACGTCTATAATCCGTTTATGATTGATGTTCAACAATGG   18776
Alpha_737204         CATCATTCTATTGGATTTGATTACGTCTATAATCCGTTTATGATTGATGTTCAACAATGG   18771
Delta_2183060        CATCATTCTATTGGATTTGATTACGTCTATAATCCGTTTATGATTGATGTTCAACAATGG   18769
Omicron_7869197      CATCATTCTATTGGATTTGATTACGTCTATAATCCGTTTATGATTGATGTTCAACAATGG   18766
                     ************************************************************

w.t._745046          GGTTTTACAGGTAACCTACAAAGCAACCATGATCTGTATTGTCAAGTCCATGGTAATGCA   18836
Alpha_737204         GGTTTTACAGGTAACCTACAAAGCAACCATGATCTGTATTGTCAAGTCCATGGTAATGCA   18831
Delta_2183060        GGTTTTACAGGTAACCTACAAAGCAACCATGATCTGTATTGTCAAGTCCATGGTAATGCA   18829
Omicron_7869197      GGTTTTACAGGTAACCTACAAAGCAACCATGATCTGTATTGTCAAGTCCATGGTAATGCA   18826
                     ************************************************************

w.t._745046          CATGTAGCTAGTTGTGATGCAATCATGACTAGGTGTCTAGCTGTCCACGAGTGCTTTGTT   18896
Alpha_737204         CATGTAGCTAGTTGTGATGCAATCATGACTAGGTGTCTAGCTGTCCACGAGTGCTTTGTT   18891
Delta_2183060        CATGTAGCTAGTTGTGATGCAATCATGACTAGGTGTCTAGCTGTCCACGAGTGCTTTGTT   18889
Omicron_7869197      CATGTAGCTAGTTGTGATGCAATCATGACTAGGTGTCTAGCTGTCCACGAGTGCTTTGTT   18886
                     ************************************************************

w.t._745046          AAGCGTGTTGACTGGACTATTGAATATCCTATAATTGGTGATGAACTGAAGATTAATGCG   18956
Alpha_737204         AAGCGTGTTGACTGGACTATTGAATATCCTATAATTGGTGATGAACTGAAGATTAATGCG   18951
Delta_2183060        AAGCGTGTTGACTGGACTATTGAATATCCTATAATTGGTGATGAACTGAAGATTAATGCG   18949
Omicron_7869197      AAGCGTGTTGACTGGACTATTGAATATCCTATAATTGGTGATGAACTGAAGATTAATGCG   18946
                     ************************************************************

w.t._745046          GCTTGTAGAAAGGTTCAACACATGGTTGTTAAAGCTGCATTATTAGCAGACAAATTCCCA   19016
Alpha_737204         GCTTGTAGAAAGGTTCAACACATGGTTGTTAAAGCTGCATTATTAGCAGACAAATTCCCA   19011
Delta_2183060        GCTTGTAGAAAGGTTCAACACATGGTTGTTAAAGCTGCATTATTAGCAGACAAATTCCCA   19009
Omicron_7869197      GCTTGTAGAAAGGTTCAACACATGGTTGTTAAAGCTGCATTATTAGCAGACAAATTCCCA   19006
                     ************************************************************

w.t._745046          GTTCCTCACGACATTGGTAACCCTAAAGCTATTAAGTGTGTACCTCAAGCTGATGTAGAA   19076
Alpha_737204         GTTCCTCACGACATTGGTAACCCTAAAGCTATTAAGTGTGTACCTCAAGCTGATGTAGAA   19071
Delta_2183060        GTTCCTCACGACATTGGTAACCCTAAAGCTATTAAGTGTGTACCTCAAGCTGATGTAGAA   19069
Omicron_7869197      GTTCCTCACGACATTGGTAACCCTAAAGCTATTAAGTGTGTACCTCAAGCTGATGTAGAA   19066
                     ************************************************************

w.t._745046          TGGAAGTTCTATGATGCACAGCCTTGTAGTGACAAAGCTTATAAAATAGAAGAATTATTC   19136
Alpha_737204         TGGAAGTTCTATGATGCACAGCCTTGTAGTGACAAAGCTTATAAAATAGAAGAATTATTC   19131
Delta_2183060        TGGAAGTTCTATGATGCACAGCCTTGTAGTGACAAAGCTTATAAAATAGAAGAATTATTC   19129
Omicron_7869197      TGGAAGTTCTATGATGCACAGCCTTGTAGTGACAAAGCTTATAAAATAGAAGAATTATTC   19126
                     ************************************************************

w.t._745046          TATTCTTATGCCACACATTCTGACAAATTCACAGATGGTGTATGCCTATTTTGGAATTGC   19196
Alpha_737204         TATTCTTATGCCACACATTCTGACAAATTCACAGATGGTGTATGCCTATTTTGGAATTGC   19191
Delta_2183060        TATTCTTATGCCACACATTCTGACAAATTCACAGATGGTGTATGCCTATTTTGGAATTGC   19189
Omicron_7869197      TATTCTTATGCCACACATTCTGACAAATTCACAGATGGTGTATGCCTATTTTGGAATTGC   19186
                     ************************************************************
```

```
w.t._745046          AATGTCGATAGATATCCTGCTAATTCCATTGTTTGTAGATTTGACACTAGAGTGCTATCT   19256
Alpha_737204         AATGTCGATAGATATCCTGCTAATTCCATTGTTTGTAGATTTGACACTAGAGTGCTATCT   19251
Delta_2183060        AATGTCGATAGATATCCTGCTAATTCCATTGTTTGTAGATTTGACACTAGAGTGCTATCT   19249
Omicron_7869197      AATGTCGATAGATATCCTGCTAATTCCATTGTTTGTAGATTTGACACTAGAGTGCTATCT   19246
                     ************************************************************

w.t._745046          AACCTTAACTTGCCTGGTTGTGATGGTGGCAGTTTGTATGTAAATAAACATGCATTCCAC   19316
Alpha_737204         AACCTTAACTTGCCTGGTTGTGATGGTGGCAGTTTGTATGTAAATAAACATGCATTCCAC   19311
Delta_2183060        AACCTTAACTTGCCTGGTTGTGATGGTGGCAGTTTGTATGTAAATAAACATGCATTCCAC   19309
Omicron_7869197      AACCTTAACTTGCCTGGTTGTGATGGTGGCAGTTTGTATGTAAATAAACATGCATTCCAC   19306
                     ************************************************************

w.t._745046          ACACCAGCTTTTGATAAAAGTGCTTTTGTTAATTTAAAACAATTACCATTTTTCTATTAC   19376
Alpha_737204         ACACCAGCTTTTGATAAAAGTGCTTTTGTTAATTTAAAACAATTACCATTTTTCTATTAC   19371
Delta_2183060        ACACCAGCTTTTGATAAAAGTGCTTTTGTTAATTTAAAACAATTACCATTTTTCTATTAC   19369
Omicron_7869197      ACACCAGCTTTTGATAAAAGTGCTTTTGTTAATTTAAAACAATTACCATTTTTCTATTAC   19366
                     ************************************************************

w.t._745046          TCTGACAGTCCATGTGAGTCTCATGGAAAACAAGTAGTGTCAGATATAGATTATGTACCA   19436
Alpha_737204         TCTGACAGTCCATGTGAGTCTCATGGAAAACAAGTAGTGTCAGATATAGATTATGTACCA   19431
Delta_2183060        TCTGACAGTCCATGTGAGTCTCATGGAAAACAAGTAGTGTCAGATATAGATTATGTACCA   19429
Omicron_7869197      TCTGACAGTCCATGTGAGTCTCATGGAAAACAAGTAGTGTCAGATATAGATTATGTACCA   19426
                     ************************************************************

w.t._745046          CTAAAGTCTGCTACGTGTATAACACGTTGCAATTTAGGTGGTGCTGTCTGTAGACATCAT   19496
Alpha_737204         CTAAAGTCTGCTACGTGTATAACACGTTGCAATTTAGGTGGTGCTGTCTGTAGACATCAT   19491
Delta_2183060        CTAAAGTCTGCTACGTGTATAACACGTTGCAATTTAGGTGGTGCTGTCTGTAGACATCAT   19489
Omicron_7869197      CTAAAGTCTGCTACGTGTATAACACGTTGCAATTTAGGTGGTGCTGTCTGTAGACATCAT   19486
                     ************************************************************

w.t._745046          GCTAATGAGTACAGATTGTATCTCGATGCTTATAACATGATGATCTCAGCTGGCTTTAGC   19556
Alpha_737204         GCTAATGAGTACAGATTGTATCTCGATGCTTATAACATGATGATCTCAGCTGGCTTTAGC   19551
Delta_2183060        GCTAATGAGTACAGATTGTATCTCGATGCTTATAACATGATGATCTCAGCTGGCTTTAGC   19549
Omicron_7869197      GCTAATGAGTACAGATTGTATCTCGATGCTTATAACATGATGATCTCAGCTGGCTTTAGC   19546
                     ************************************************************

w.t._745046          TTGTGGGTTTACAAACAATTTGATACTTATAACCTCTGGAACACTTTTACAAGACCTCAG   19616
Alpha_737204         TTGTGGGTTTACAAACAATTTGATACTTATAACCTCTGGAACACTTTTACAAGACCTCAG   19611
Delta_2183060        TTGTGGGTTTACAAACAATTTGATACTTATAACCTCTGGAACACTTTTACAAGACCTCAG   19609
Omicron_7869197      TTGTGGGTTTACAAACAATTTGATACTTATAACCTCTGGAACACTTTTACAAGACCTCAG   19606
                     ************************************************************

w.t._745046          AGTTTAGAAAATGTGGCTTTTAATGTTGTAAATAAGGGACACTTTGATGGACAACAGGGT   19676
Alpha_737204         AGTTTAGAAAATGTGGCTTTTAATGTTGTAAATAAGGGACACTTTGATGGACAACAGGGT   19671
Delta_2183060        AGTTTAGAAAATGTGGCTTTTAATGTTGTAAATAAGGGACACTTTGATGGACAACAGGGT   19669
Omicron_7869197      AGTTTAGAAAATGTGGCTTTTAATGTTGTAAATAAGGGACACTTTGATGGACAACAGGGT   19666
                     ************************************************************

w.t._745046          GAAGTACCAGTTTCTATCATTAATAACACTGTTTACACAAAAGTTGATGGTGTTGATGTA   19736
Alpha_737204         GAAGTACCAGTTTCTATCATTAATAACACTGTTTACACAAAAGTTGATGGTGTTGATGTA   19731
Delta_2183060        GAAGTACCAGTTTCTATCATTAATAACACTGTTTACACAAAAGTTGATGGTGTTGATGTA   19729
Omicron_7869197      GAAGTACCAGTTTCTATCATTAATAACACTGTTTACACAAAAGTTGATGGTGTTGATGTA   19726
                     ************************************************************

w.t._745046          GAATTGTTTGAAAATAAAACAACATTACCTGTTAATGTAGCATTTGAGCTTTGGGCTAAG   19796
Alpha_737204         GAATTGTTTGAAAATAAAACAACATTACCTGTTAATGTAGCATTTGAGCTTTGGGCTAAG   19791
Delta_2183060        GAATTGTTTGAAAATAAAACAACATTACCTGTTAATGTAGCATTTGAGCTTTGGGCTAAG   19789
Omicron_7869197      GAATTGTTTGAAAATAAAACAACATTACCTGTTAATGTAGCATTTGAGCTTTGGGCTAAG   19786
                     ************************************************************
```

```
w.t._745046        CGCAACATTAAACCAGTACCAGAGGTGAAAATACTCAATAATTTGGGTGTGGACATTGCT   19856
Alpha_737204       CGCAACATTAAACCAGTACCAGAGGTGAAAATACTCAATAATTTGGGTGTGGACATTGCT   19851
Delta_2183060      CGCAACATTAAACCAGTACCAGAGGTGAAAATACTCAATAATTTGGGTGTGGACATTGCT   19849
Omicron_7869197    CGCAACATTAAACCAGTACCAGAGGTGAAAATACTCAATAATTTGGGTGTGGACATTGCT   19846
                   ************************************************************

w.t._745046        GCTAATACTGTGATCTGGGACTACAAAAGAGATGCTCCAGCACATATATCTACTATTGGT   19916
Alpha_737204       GCTAATACTGTGATCTGGGACTACAAAAGAGATGCTCCAGCACATATATCTACTATTGGT   19911
Delta_2183060      GCTAATACTGTGATCTGGGACTACAAAAGAGATGCTCCAGCACATATATCTACTATTGGT   19909
Omicron_7869197    GCTAATACTGTGATCTGGGACTACAAAAGAGATGCTCCAGCACATATATCTACTATTGGT   19906
                   ************************************************************

w.t._745046        GTTTGTTCTATGACTGACATAGCCAAGAAACCAACTGAAACGATTTGTGCACCACTCACT   19976
Alpha_737204       GTTTGTTCTATGACTGACATAGCCAAGAAACCAACTGAAACGATTTGTGCACCACTCACT   19971
Delta_2183060      GTTTGTTCTATGACTGACATAGCCAAGAAACCAACTGAAACGATTTGTGCACCACTCACT   19969
Omicron_7869197    GTTTGTTCTATGACTGACATAGCCAAGAAACCAACTGAAACGATTTGTGCACCACTCACT   19966
                   ************************************************************

w.t._745046        GTCTTTTTTGATGGTAGAGTTGATGGTCAAGTAGACTTATTTAGAAATGCCCGTAATGGT   20036
Alpha_737204       GTCTTTTTTGATGGTAGAGTTGATGGTCAAGTAGACTTATTTAGAAATGCCCGTAATGGT   20031
Delta_2183060      GTCTTTTTTGATGGTAGAGTTGATGGTCAAGTAGACTTATTTAGAAATGCCCGTAATGGT   20029
Omicron_7869197    GTCTTTTTTGATGGTAGAGTTGATGGTCAAGTAGACTTATTTAGAAATGCCCGTAATGGT   20026
                   ************************************************************

w.t._745046        GTTCTTATTACAGAAGGTAGTGTTAAAGGTTTACAACCATCTGTAGGTCCCAAACAAGCT   20096
Alpha_737204       GTTCTTATTACAGAAGGTAGTGTTAAAGGTTTACAACCATCTGTAGGTCCCAAACAAGCT   20091
Delta_2183060      GTTCTTATTACAGAAGGTAGTGTTAAAGGTTTACAACCATCTGTAGGTCCCAAACAAGCT   20089
Omicron_7869197    GTTCTTATTACAGAAGGTAGTGTTAAAGGTTTACAACCATCTGTAGGTCCCAAACAAGCT   20086
                   ************************************************************

w.t._745046        AGTCTTAATGGAGTCACATTAATTGGAGAAGCCGTAAAAACACAGTTCAATTATTATAAG   20156
Alpha_737204       AGTCTTAATGGAGTCACATTAATTGGAGAAGCCGTAAAAACACAGTTCAATTATTATAAG   20151
Delta_2183060      AGTCTTAATGGAGTCACATTAATTGGAGAAGCCGTAAAAACACAGTTCAATTATTATAAG   20149
Omicron_7869197    AGTCTTAATGGAGTCACATTAATTGGAGAAGCCGTAAAAACACAGTTCAATTATTATAAG   20146
                   ************************************************************

w.t._745046        AAAGTTGATGGTGTTGTCCAACAATTACCTGAAACTTACTTTACTCAGAGTAGAAATTTA   20216
Alpha_737204       AAAGTTGATGGTGTTGTCCAACAATTACCTGAAACTTACTTTACTCAGAGTAGAAATTTA   20211
Delta_2183060      AAAGTTGATGGTGTTGTCCAACAATTACCTGAAACTTACTTTACTCAGAGTAGAAATTTA   20209
Omicron_7869197    AAAGTTGATGGTGTTGTCCAACAATTACCTGAAACTTACTTTACTCAGAGTAGAAATTTA   20206
                   ************************************************************

w.t._745046        CAAGAATTTAAACCCAGGAGTCAAATGGAAATTGATTTCTTAGAATTAGCTATGGATGAA   20276
Alpha_737204       CAAGAATTTAAACCCAGGAGTCAAATGGAAATTGATTTCTTAGAATTAGCTATGGATGAA   20271
Delta_2183060      CAAGAATTTAAACCCAGGAGTCAAATGGAAATTGATTTCTTGGAATTAGCTATGGATGAA   20269
Omicron_7869197    CAAGAATTTAAACCCAGGAGTCAAATGGAAATTGATTTCTTAGAATTAGCTATGGATGAA   20266
                   ***************************************** ******************

w.t._745046        TTCATTGAACGGTATAAATTAGAAGGCTATGCCCTCGAACATATCGTTTATGGAGATTTT   20336
Alpha_737204       TTCATTGAACGGTATAAATTAGAAGGCTATGCCCTCGAACATATCGTTTATGGAGATTTT   20331
Delta_2183060      TTCATTGAACGGTATAAATTAGAAGGCTATGCCCTCGAATATATCGTTTATGGAGATTTT   20329
Omicron_7869197    TTCATTGAACGGTATAAATTAGAAGGCTATGCCCTCGAACATATCGTTTATGGAGATTTT   20326
                   *************************************** ********************

w.t._745046        AGTCATAGTCAGTTAGGTGGTTTACATCTACTGATTGGACTAGCTAAACGTTTTAAGGAA   20396
Alpha_737204       AGTCATAGTCAGTTAGGTGGTTTACATCTACTGATTGGACTAGCTAAACGTTTTAAGGAA   20391
Delta_2183060      AGTCATAGTCAGTTAGGTGGTTTACATCTACTGATTGGACTAGCTAAACGTTTTAAGGAA   20389
Omicron_7869197    AGTCATAGTCAGTTAGGTGGTTTACATCTACTGATTGGACTAGCTAAACGTTTTAAGGAA   20386
                   ************************************************************
```

```
w.t._745046          TCACCTTTTGAATTAGAAGATTTTATTCCTATGGACAGTACAGTTAAAAACTATTTCATA   20456
Alpha_737204         TCACCTTTTGAATTAGAAGATTTTATTCCTATGGACAGTACAGTTAAAAACTATTTCATA   20451
Delta_2183060        TCACCTTTTGAATTAGAAGATTTTATTCCTATGGACAGTACAGTTAAAAACTATTTCATA   20449
Omicron_7869197      TCACCTTTTGAATTAGAAGATTTTATTCCTATGGACAGTACAGTTAAAAACTATTTCATA   20446
                     ************************************************************

w.t._745046          ACAGATGCGCAAACAGGTTCATCTAAGTGTGTGTGTTCTGTTATTGATTTATTACTTGAT   20516
Alpha_737204         ACAGATGCGCAAACAGGTTCATCTAAGTGTGTGTGTTCTGTTATTGATTTATTACTTGAT   20511
Delta_2183060        ACAGATGCGCAAACAGGTTCATCTAAGTGTGTGTGTTCTGTTATTGATTTATTACTTGAT   20509
Omicron_7869197      ACAGATGCGCAAACAGGTTCATCTAAGTGTGTGTGTTCTGTTATTGATTTATTACTTGAT   20506
                     ************************************************************

w.t._745046          GATTTTGTTGAAATAATAAAATCCCAAGATTTATCTGTAGTTTCTAAGGTTGTCAAAGTG   20576
Alpha_737204         GATTTTGTTGAAATAATAAAATCCCAAGATTTATCTGTAGTTTCTAAGGTTGTCAAAGTG   20571
Delta_2183060        GATTTTGTTGAAATAATAAAATCCCAAGATTTATCTGTAGTTTCTAAGGTTGTCAAAGTG   20569
Omicron_7869197      GATTTTGTTGAAATAATAAAATCCCAAGATTTATCTGTAGTTTCTAAGGTTGTCAAAGTG   20566
                     ************************************************************

w.t._745046          ACTATTGACTATACAGAAATTTCATTTATGCTTTGGTGTAAAGATGGCCATGTAGAAACA   20636
Alpha_737204         ACTATTGACTATACAGAAATTTCATTTATGCTTTGGTGTAAAGATGGCCATGTAGAAACA   20631
Delta_2183060        ACTATTGACTATACAGAAATTTCATTTATGCTTTGGTGTAAAGATGGCCATGTAGAAACA   20629
Omicron_7869197      ACTATTGACTATACAGAAATTTCATTTATGCTTTGGTGTAAAGATGGCCATGTAGAAACA   20626
                     ************************************************************

w.t._745046          TTTTACCCAAAATTACAATCTAGTCAAGCGTGGCAACCGGGTGTTGCTATGCCTAATCTT   20696
Alpha_737204         TTTTACCCAAAATTACAATCTAGTCAAGCGTGGCAACCGGGTGTTGCTATGCCTAATCTT   20691
Delta_2183060        TTTTACCCAAAATTACAATCTAGTCAAGCGTGGCAACCGGGTGTTGCTATGCCTAATCTT   20689
Omicron_7869197      TTTTACCCAAAATTACAATCTAGTCAAGCGTGGCAACCGGGTGTTGCTATGCCTAATCTT   20686
                     ************************************************************

w.t._745046          TACAAAATGCAAAGAATGCTATTAGAAAAGTGTGACCCTCAAAATTATGGTGATAGTGCA   20756
Alpha_737204         TACAAAATGCAAAGAATGCTATTAGAAAAGTGTGACCCTCAAAATTATGGTGATAGTGCA   20751
Delta_2183060        TACAAAATGCAAAGAATGCTATTAGAAAAGTGTGACCCTCAAAATTATGGTGATAGTGCA   20749
Omicron_7869197      TACAAAATGCAAAGAATGCTATTAGAAAAGTGTGACCCTCAAAATTATGGTGATAGTGCA   20746
                     ************************************************************

w.t._745046          ACATTACCTAAAGGCATAATGATGAATGTCGCAAAATATACTCAACTGTGTCAATATTTA   20816
Alpha_737204         ACATTACCTAAAGGCATAATGATGAATGTCGCAAAATATACTCAACTGTGTCAATATTTA   20811
Delta_2183060        ACATTACCTAAAGGCATAATGATGAATGTCGCAAAATATACTCAACTGTGTCAATATTTA   20809
Omicron_7869197      ACATTACCTAAAGGCATAATGATGAATGTCGCAAAATATACTCAACTGTGTCAATATTTA   20806
                     ************************************************************

w.t._745046          AACACATTAACATTAGCTGTACCCTATAATATGAGAGTTATACATTTTGGTGCTGGTTCT   20876
Alpha_737204         AACACATTAACATTAGCTGTACCCTATAATATGAGAGTTATACATTTTGGTGCTGGTTCT   20871
Delta_2183060        AACACATTAACATTAGCTGTACCCTATAATATGAGAGTTATACATTTTGGTGCTGGTTCT   20869
Omicron_7869197      AACACATTAACATTAGCTGTACCCTATAATATGAGAGTTATACATTTTGGTGCTGGTTCT   20866
                     ************************************************************

w.t._745046          GATAAAGGAGTTGCACCAGGTACAGCTGTTTTAAGACAGTGGTTGCCTACGGGTACGCTG   20936
Alpha_737204         GATAAAGGAGTTGCACCAGGTACAGCTGTTTTAAGACAGTGGTTGCCTACGGGTACGCTG   20931
Delta_2183060        GATAAAGGAGTTGCACCAGGTACAGCTGTTTTAAGACAGTGGTTGCCTACGGGTACGCTG   20929
Omicron_7869197      GATAAAGGAGTTGCACCAGGTACAGCTGTTTTAAGACAGTGGTTGCCTACGGGTACGCTG   20926
                     ************************************************************

w.t._745046          CTTGTCGATTCAGATCTTAATGACTTTGTCTCTGATGCAGATTCAACTTTGATTGGTGAT   20996
Alpha_737204         CTTGTCGATTCAGATCTTAATGACTTTGTCTCTGATGCAGATTCAACTTTGATTGGTGAT   20991
Delta_2183060        CTTGTCGATTCAGATCTTAATGACTTTGTCTCTGATGCAGATTCAACTTTGATTGGTGAT   20989
Omicron_7869197      CTTGTCGATTCAGATCTTAATGACTTTGTCTCTGATGCAGATTCAACTTTGATTGGTGAT   20986
                     ************************************************************
```

```
w.t._745046         TGTGCAACTGTACATACAGCTAATAAATGGGATCTCATTATTAGTGATATGTACGACCCT   21056
Alpha_737204        TGTGCAACTGTACATACAGCTAATAAATGGGATCTCATTATTAGTGATATGTACGACCCT   21051
Delta_2183060       TGTGCAACTGTACATACAGCTAATAAATGGGATCTCATTATTAGTGATATGTACGACCCT   21049
Omicron_7869197     TGTGCAACTGTACATACAGCTAATAAATGGGATCTCATTATTAGTGATATGTACGACCCT   21046
                    ************************************************************

w.t._745046         AAGACTAAAAATGTTACAAAAGAAAATGACTCTAAAGAGGGTTTTTTCACTTACATTTGT   21116
Alpha_737204        AAGACTAAAAATGTTACAAAAGAAAATGACTCTAAAGAGGGTTTTTTCACTTACATTTGT   21111
Delta_2183060       AAGACTAAAAATGTTACAAAAGAAAATGACTCTAAAGAGGGTTTTTTCACTTACATTTGT   21109
Omicron_7869197     AAGACTAAAAATGTTACAAAAGAAAATGACTCTAAAGAGGGTTTTTTCACTTACATTTGT   21106
                    ************************************************************

w.t._745046         GGGTTTATACAACAAAGGCTAGCTCTTGGAGGTTCCGTGGCTATAAAGATAACAGAACAT   21176
Alpha_737204        GGGTTTATACAACAAAAGCTAGCTCTTGGAGGTTCCGTGGCTATAAAGATAACAGAACAT   21171
Delta_2183060       GGGTTTATACAACAAAAGCTAGCTCTTGGAGGTTCCGTGGCTATAAAGATAACAGAACAT   21169
Omicron_7869197     GGGTTTATACAACAAAAGCTAGCTCTTGGAGGTTCCGTGGCTATAAAGATAACAGAACAT   21166
                    **************** *******************************************

w.t._745046         TCTTGGAATGCTGATCTTTATAAGCTCATGGGACACCTCGCATGGTGGACAGCCTTTGTT   21236
Alpha_737204        TCTTGGAATGCTGATCTTTATAAGCTCATGGGACACCTCGCATGGTGGACAGCCTTTGTT   21231
Delta_2183060       TCTTGGAATGCTGATCTTTATAAGCTCATGGGACACCTCGCATGGTGGACAGCCTTTGTT   21229
Omicron_7869197     TCTTGGAATGCTGATCTTTATAAGCTCATGGGACACCTCGCATGGTGGACAGCCTTTGTT   21226
                    ************************************************************

w.t._745046         ACTAATGTGAATGCGTCATCATCTGAAGCATTTTTAATTGGATGTAATTATCTTGGCAAA   21296
Alpha_737204        ACTAATGTGAATGCGTCATCATCTGAAGCATTTTTAATTGGATGTAATTATCTTGGCAAA   21291
Delta_2183060       ACTAATGTGAATGCGTCATCATCTGAAGCATTTTTAATTGGATGTAATTATCTTGGCAAA   21289
Omicron_7869197     ACTAATGTGAATGCGTCATCATCTGAAGCATTTTTAATTGGATGTAATTATCTTGGCAAA   21286
                    ************************************************************

w.t._745046         CCACGCGAACAAATAGATGGTTATGTCATGCATGCAAATTACATATTTTGGAGGAATACA   21356
Alpha_737204        CCACGCGAACAAATAGATGGTTATGTCATGCATGCAAATTACATATTTTGGAGGAATACA   21351
Delta_2183060       CCACGCGAACAAATAGATGGTTATGTCATGCATGCAAATTACATATTTTGGAGGAATACA   21349
Omicron_7869197     CCACGCGAACAAATAGATGGTTATGTCATGCATGCAAATTACATATTTTGGAGGAATACA   21346
                    ************************************************************

w.t._745046         AATCCAATTCAGTTGTCCTCCTATTCTTTATTTGACATGAGTAAATTTCCCCTTAAATTA   21416
Alpha_737204        AATCCAATTCAGTTGTCCTCCTATTCTTTATTTGACATGAGTAAATTTCCCCTTAAATTA   21411
Delta_2183060       AATCCAATTCAGTTGTCCTCCTATTCTTTATTTGACATGAGTAAATTTCCCCTTAAATTA   21409
Omicron_7869197     AATCCAATTCAGTTGTCCTCCTATTCTTTATTTGACATGAGTAAATTTCCCCTTAAATTA   21406
                    ************************************************************

w.t._745046         AGGGGTACTGCTGTTATGTCTTTAAAAGAAGGTCAAATCAATGATATGATTTTATCTCTT   21476
Alpha_737204        AGGGGTACTGCTGTTATGTCTTTAAAAGAAGGTCAAATCAATGATATGATTTTATCTCTT   21471
Delta_2183060       AGGGGTACTGCTGTTATGTCTTTAAAAGAAGGTCAAATCAATGATATGATTTTATCTCTT   21469
Omicron_7869197     AGGGGTACTGCTGTTATGTCTTTAAAAGAAGGTCAAATCAATGATATGATTTTATCTCTT   21466
                    ************************************************************

w.t._745046         CTTAGTAAAGGTAGACTTATAATTAGAGAAAACAACAGAGTTGTTATTTCTAGTGATGTT   21536
Alpha_737204        CTTAGTAAAGGTAGACTTATAATTAGAGAAAACAACAGAGTTGTTATTTCTAGTGATGTT   21531
Delta_2183060       CTTAGTAAAGGTAGACTTATAATTAGAGAAAACAACAGAGTTGTTATTTCTAGTGATGTT   21529
Omicron_7869197     CTTAGTAAAGGTAGACTTATAATTAGAGAAAACAACAGAGTTGTTATTTCTAGTGATGTT   21526
                    ************************************************************

w.t._745046         CTTGTTAACAACTAAACGAACAATGTTTGTTTTTCTTGTTTTATTGCCACTAGTCTCTAG   21596
Alpha_737204        CTTGTTAACAACTAAACGAACAATGTTTGTTTTTCTTGTTTTATTGCCACTAGTCTCTAG   21591
Delta_2183060       CTTGTTAACAACTAAACGAACAATGTTTGTTTTTCTTGTTTTATTGCCACTAGTCTCTAG   21589
Omicron_7869197     CTTGTTAACAACTAAACGAACAATGTTTGTTTTTCTTGTTTTATTGCCACTAGTCTCTAG   21586
                    ************************************************************
```

```
w.t._745046         TCAGTGTGTTAATCTTACAACCAGAACTCAATTACCCCCTGCATACACTAATTCTTTCAC   21656
Alpha_737204        TCAGTGTGTTAATCTTACAACCAGAACTCAATTACCCCCTGCATACACTAATTCTTTCAC   21651
Delta_2183060       TCAGTGTGTTAATCTTAGAACCAGAACTCAATTACCCCCTGCATACACTAATTCTTTCAC   21649
Omicron_7869197     TCAGTGTGTTAATCTTACAACCAGAACTCAATTACCCCCTGCATACACTAATTCTTTCAC   21646
                    ***************** ******************************************

w.t._745046         ACGTGGTGTTTATTACCCTGACAAAGTTTTCAGATCCTCAGTTTTACATTCAACTCAGGA   21716
Alpha_737204        ACGTGGTGTTTATTACCCTGACAAAGTTTTCAGATCCTCAGTTTTACATTCAACTCAGGA   21711
Delta_2183060       ACGTGGTGTTTATTACCCTGACAAAGTTTTCAGATCCTCAGTTTTACATTCAACTCAGGA   21709
Omicron_7869197     ACGTGGTGTTTATTACCCTGACAAAGTTTTCAGATCCTCAGTTTTACATTCAACTCAGGA   21706
                    ************************************************************

w.t._745046         CTTGTTCTTACCTTTCTTTTCCAATGTTACTTGGTTCCATGCTATACATGTCTCTGGGAC   21776
Alpha_737204        CTTGTTCTTACCTTTCTTTTCCAATGTTACTTGGTTCCATGCTA------TCTCTGGGAC   21765
Delta_2183060       CTTGTTCTTACCTTTCTTTTCCAATGTTACTTGGTTCCATGCTATACATGTCTCTGGGAC   21769
Omicron_7869197     CTTGTTCTTACCTTTCTTTTCCAATGTTACTTGGTTCCATGTTA------TCTCTGGGAC   21760
                    ***************************************** **      **********

w.t._745046         CAATGGTACTAAGAGGTTTGATAACCCTGTCCTACCATTTAATGATGGTGTTTATTTTGC   21836
Alpha_737204        CAATGGTACTAAGAGGTTTGATAACCCTGTCCTACCATTTAATGATGGTGTTTATTTTGC   21825
Delta_2183060       CAATGGTACTAAGAGGTTTGATAACCCTGTCCTACCATTTAATGATGGTGTTTATTTTGC   21829
Omicron_7869197     CAATGGTACTAAGAGGTTTGATAACCCTGTCCTACCATTTAATGATGGTGTTTATTTTGC   21820
                    ************************************************************

w.t._745046         TTCCACTGAGAAGTCTAACATAATAAGAGGCTGGATTTTTGGTACTACTTTAGATTCGAA   21896
Alpha_737204        TTCCACTGAGAAGTCTAACATAATAAGAGGCTGGATTTTTGGTACTACTTTAGATTCGAA   21885
Delta_2183060       TTCCACTGAGAAGTCTAACATAATAAGAGGCTGGATTTTTGGTACTACTTTAGATTCGAA   21889
Omicron_7869197     TTCCATTGAGAAGTCTAACATAATAAGAGGCTGGATTTTTGGTACTACTTTAGATTCGAA   21880
                    ***** ******************************************************

w.t._745046         GACCCAGTCCCTACTTATTGTTAATAACGCTACTAATGTTGTTATTAAAGTCTGTGAATT   21956
Alpha_737204        GACCCAGTCCCTACTTATTGTTAATAACGCTACTAATGTTGTTATTAAAGTCTGTGAATT   21945
Delta_2183060       GACCCAGTCCCTACTTATTGTTAATAACGCTACTAATGTTGTTATTAAAGTCTGTGAATT   21949
Omicron_7869197     GACCCAGTCCCTACTTATTGTTAATAACGCTACTAATGTTGTTATTAAAGTCTGTGAATT   21940
                    ************************************************************

w.t._745046         TCAATTTTGTAATGATCCATTTTTGGGTGTTTATTACCACAAAAACAACAAAAGTTGGAT   22016
Alpha_737204        TCAATTTTGTAATGATCCATTTTTGGGTGT---TTACCACAAAAACAACAAAAGTTGGAT   22002
Delta_2183060       TCAATTTTGTAATGATCCATTTTTGGGTGTTTATTACCACAAAAACAACAAAAGTTGGAT   22009
Omicron_7869197     TCAATTTTGTAATGATCCATTTTTGG---------ACCACAAAAACAACAAAAGTTGGAT   21991
                    **************************         *************************

w.t._745046         GGAAAGTGAGTTCAGAGTTTATTCTAGTGCGAATAATTGCACTTTTGAATATGTCTCTCA   22076
Alpha_737204        GGAAAGTGAGTTCAGAGTTTATTCTAGTGCGAATAATTGCACTTTTGAATATGTCTCTCA   22062
Delta_2183060       GGAAAGTG------GAGTTTATTCTAGTGCGAATAATTGCACTTTTGAATATGTCTCTCA   22063
Omicron_7869197     GGAAAGTGAGTTCAGAGTTTATTCTAGTGCGAATAATTGCACTTTTGAATATGTCTCTCA   22051
                    ********      **********************************************

w.t._745046         GCCTTTTCTTATGGACCTTGAAGGAAAACAGGGTAATTTCAAAAATCTTAGGGAATTTGT   22136
Alpha_737204        GCCTTTTCTTATGGACCTTGAAGGAAAACAGGGTAATTTCAAAAATCTTAGGGAATTTGT   22122
Delta_2183060       GCCTTTTCTTATGGACCTTGAAGGAAAACAGGGTAATTTCAAAAATCTTAGGGAATTTGT   22123
Omicron_7869197     GCCTTTTCTTATGGACCTTGAAGGAAAACAGGGTAATTTCAAAAATCTTAGGGAATTTGT   22111
                    ************************************************************

w.t._745046         GTTTAAGAATATTGATGGTTATTTTAAAATATATTCTAAGCACACGCCTATTAATTTAGT   22196
Alpha_737204        GTTTAAGAATATTGATGGTTATTTTAAAATATATTCTAAGCACACGCCTATTAATTTAGT   22182
Delta_2183060       GTTTAAGAATATTGATGGTTATTTTAAAATATATTCTAAGCACACGCCTATTAATTTAGT   22183
Omicron_7869197     GTTTAAGAATATTGATGGTTATTTTAAAATATATTCTAAGCACACGCCTATTA---TAGT   22168
                    *****************************************************   ****
```

```
w.t._745046         GCGTGATCTCCCTCAGGGTTTTTCGGCTTTAGAACCATTGGTAGATTTGCCAATAGGTAT   22256
Alpha_737204        GCGTGATCTCCCTCAGGGTTTTTCGGCTTTAGAACCATTGGTAGATTTGCCAATAGGTAT   22242
Delta_2183060       GCGTGATCTCCCTCAGGGTTTTTCGGCTTTAGAACCATTGGTAGATTTGCCAATAGGTAT   22243
Omicron_7869197     GCGTGATCTCCCTCAGGGTTTTTCGGCTTTAGAACCATTGGTAGATTTGCCAATAGGTAT   22228
                    ************************************************************

w.t._745046         TAACATCACTAGGTTTCAAACTTTACTTGCTTTACATAGAAGTTATTTGACTCCTGGTGA   22316
Alpha_737204        TAACATCACTAGGTTTCAAACTTTACTTGCTTTACATAGAAGTTATTTGACTCCTGGTGA   22302
Delta_2183060       TAACATCACTAGGTTTCAAACTTTACTTGCTTTACATAGAAGTTATTTGACTCCTGGTGA   22303
Omicron_7869197     TAACATCACTAGGTTTCAAACTTTACTTGCTTTACATAGAAGTTATTTGACTCCTGGTGA   22288
                    ************************************************************

w.t._745046         TTCCTCTTCAGGTTGGACAGCTGGTGCTGCAGCTTATTATGTGGGTTATCCTCAACCTAG   22376
Alpha_737204        TTCCTCTTCAGGTTGGACAGCTGGTGCTGCAGCTTATTATGTGGGTTATCCTCAACCTAG   22362
Delta_2183060       TTCCTCTTCAGGTTGGACAGCTGGTGCTGCAGCTTATTATGTGGGTTATCCTCAACCTAG   22363
Omicron_7869197     TTCCTCTTCAGGTTGGACAGCTGGTGCTGCAGCTTATTATGTGGGTTATCCTCAACCTAG   22348
                    ************************************************************

w.t._745046         GACTTTTCTATTAAAATATAATGAAAATGGAACCATTACAGATGCTGTAGACTGTGCACT   22436
Alpha_737204        GACTTTTCTATTAAAATATAATGAAAATGGAACCATTACAGATGCTGTAGACTGTGCACT   22422
Delta_2183060       GACTTTTCTATTAAAATATAATGAAAATGGAACCATTACAGATGCTGTAGACTGTGCACT   22423
Omicron_7869197     GACTTTTCTATTAAAATATAATGAAAATGGAACCATTACAGATGCTGTAGACTGTGCACT   22408
                    ************************************************************

w.t._745046         TGACCCTCTCTCAGAAACAAAGTGTACGTTGAAATCCCTCACTGTAGAAAAAGGAATCTA   22496
Alpha_737204        TGACCCTCTCTCAGAAACAAAGTGTACGTTGAAATCCCTCACTGTAGAAAAAGGAATCTA   22482
Delta_2183060       TGACCCTCTCTCAGAAACAAAGTGTACGTTGAAATCCCTCACTGTAGAAAAAGGAATCTA   22483
Omicron_7869197     TGACCCTCTCTCAGAAACAAAGTGTACGTTGAAATCCCTCACTGTAGAAAAAGGAATCTA   22468
                    ************************************************************

w.t._745046         TCAAACCTCTAACTTTAGAGTCCAACCAACAGAATCTATTGTTAGATTTCCTAATATTAC   22556
Alpha_737204        TCAAACCTCTAACTTTAGAGTCCAACCAACAGAATCTATTGTTAGATTTCCTAATATTAC   22542
Delta_2183060       TCAAACCTCTAACTTTAGAGTCCAACCAACAGAATCTATTGTTAGATTTCCTAATATTAC   22543
Omicron_7869197     TCAAACCTCTAACTTTAGAGTCCAACCAACAGAATCTATTGTTAGATTTCCTAATATTAC   22528
                    ************************************************************

w.t._745046         AAACTTGTGCCCTTTTGGTGAAGTTTTTAACGCCACCAGATTTGCATCTGTTTATGCTTG   22616
Alpha_737204        AAACTTGTGCCCTTTTGGTGAAGTTTTTAACGCCACCAGATTTGCATCTGTTTATGCTTG   22602
Delta_2183060       AAACTTGTGCCCTTTTGGTGAAGTTTTTAACGCCACCAGATTTGCATCTGTTTATGCTTG   22603
Omicron_7869197     AAACTTGTGCCCTTTTGATGAAGTTTTTAACGCCACCAGATTTGCATCTGTTTATGCTTG   22588
                    ***************** ******************************************

w.t._745046         GAACAGGAAGAGAATCAGCAACTGTGTTGCTGATTATTCTGTCCTATATAATTCCGCATC   22676
Alpha_737204        GAACAGGAAGAGAATCAGCAACTGTGTTGCTGATTATTCTGTCCTATATAATTCCGCATC   22662
Delta_2183060       GAACAGGAAGAGAATCAGCAACTGTGTTGCTGATTATTCTGTCCTATATAATTCCGCATC   22663
Omicron_7869197     GAACAGGAAGAGAATCAGCAACTGTGTTGCTGATTATTCTGTCCTATATAATCTCGCACC   22648
                    ****************************************************  **** *

w.t._745046         ATTTTCCACTTTTAAGTGTTATGGAGTGTCTCCTACTAAATTAAATGATCTCTGCTTTAC   22736
Alpha_737204        ATTTTCCACTTTTAAGTGTTATGGAGTGTCTCCTACTAAATTAAATGATCTCTGCTTTAC   22722
Delta_2183060       ATTTTCCACTTTTAAGTGTTATGGAGTGTCTCCTACTAAATTAAATGATCTCTGCTTTAC   22723
Omicron_7869197     ATTTTTCACTTTTAAGTGTTATGGAGTGTCTCCTACTAAATTAAATGATCTCTGCTTTAC   22708
                    ***** ******************************************************

w.t._745046         TAATGTCTATGCAGATTCATTTGTAATTAGAGGTGATGAAGTCAGACAAATCGCTCCAGG   22796
Alpha_737204        TAATGTCTATGCAGATTCATTTGTAATTAGAGGTGATGAAGTCAGACAAATCGCTCCAGG   22782
Delta_2183060       TAATGTCTATGCAGATTCATTTGTAATTAGAGGTGATGAAGTCAGACAAATCGCTCCAGG   22783
Omicron_7869197     TAATGTCTATGCAGATTCATTTGTAATTAGAGGTGATGAAGTCAGACAAATCGCTCCAGG   22768
                    ************************************************************
```

```
w.t._745046         GCAAACTGGAAAGATTGCTGATTATAATTATAAATTACCAGATGATTTTACAGGCTGCGT   22856
Alpha_737204        GCAAACTGGAAAGATTGCTGATTATAATTATAAATTACCAGATGATTTTACAGGCTGCGT   22842
Delta_2183060       GCAAACTGGAAAGATTGCTGATTATAATTATAAATTACCAGATGATTTTACAGGCTGCGT   22843
Omicron_7869197     GCAAACTGGAAATATTGCTGATTATAATTATAAATTACCAGATGATTTTACAGGCTGCGT   22828
                    ************ ***********************************************

w.t._745046         TATAGCTTGGAATTCTAACAATCTTGATTCTAAGGTTGGTGGTAATTATAATTACCTGTA   22916
Alpha_737204        TATAGCTTGGAATTCTAACAATCTTGATTCTAAGGTTGGTGGTAATTATAATTACCTGTA   22902
Delta_2183060       TATAGCTTGGAATTCTAACAATCTTGATTCTAAGGTTGGTGGTAATTATAATTACCGGTA   22903
Omicron_7869197     TATAGCTTGGAATTCTAACAAGCTTGATTCTAAGGTTAGTGGTAATTATAATTACCTGTA   22888
                    ********************* *************** ****************** ***

w.t._745046         TAGATTGTTTAGGAAGTCTAATCTCAAACCTTTTGAGAGAGATATTTCAACTGAAATCTA   22976
Alpha_737204        TAGATTGTTTAGGAAGTCTAATCTCAAACCTTTTGAGAGAGATATTTCAACTGAAATCTA   22962
Delta_2183060       TAGATTGTTTAGGAAGTCTAATCTCAAACCTTTTGAGAGAGATATTTCAACTGAAATCTA   22963
Omicron_7869197     TAGATTGTTTAGGAAGTCTAATCTCAAACCTTTTGAGAGAGATATTTCAACTGAAATCTA   22948
                    ************************************************************

w.t._745046         TCAGGCCGGTAGCACACCTTGTAATGGTGTTGAAGGTTTTAATTGTTACTTTCCTTTACA   23036
Alpha_737204        TCAGGCCGGTAGCACACCTTGTAATGGTGTTGAAGGTTTTAATTGTTACTTTCCTTTACA   23022
Delta_2183060       TCAGGCCGGTAGCAAACCTTGTAATGGTGTTGAAGGTTTTAATTGTTACTTTCCTTTACA   23023
Omicron_7869197     TCAGGCCGGTAACAAACCTTGTAATGGTGTTGCAGGTTTTAATTGTTACTTTCCTTTACG   23008
                    *********** ** ***************** ************************** 

w.t._745046         ATCATATGGTTTCCAACCCACTAATGGTGTTGGTTACCAACCATACAGAGTAGTAGTACT   23096
Alpha_737204        ATCATATGGTTTCCAACCCACTTATGGTGTTGGTTACCAACCATACAGAGTAGTAGTACT   23082
Delta_2183060       ATCATATGGTTTCCAACCCACTAATGGTGTTGGTTACCAACCATACAGAGTAGTAGTACT   23083
Omicron_7869197     ATCATATAGTTTCCGACCCACTTATGGTGTTGGTCACCAACCATACAGAGTAGTAGTACT   23068
                    ******* ****** ******* *********** *************************

w.t._745046         TTCTTTTGAACCTCTACATGCACCAGCAACTGTTTGTGGACCTAAAAAGTCTACTAATTT   23156
Alpha_737204        TTCTTTTGAACCTCTACATGCACCAGCAACTGTTTGTGGACCTAAAAAGTCTACTAATTT   23142
Delta_2183060       TTCTTTTGAACCTCTACATGCACCAGCAACTGTTTGTGGACCTAAAAAGTCTACTAATTT   23143
Omicron_7869197     TTCTTTTGAACCTCTACATGCACCAGCAACTGTTTGTGGACCTAAAAAGTCTACTAATTT   23128
                    ************************************************************

w.t._745046         GGTTAAAAACAAATGTGTCAATTTCAACCTCAATGGTTTAACAGGCACAGGTGTTCTTAC   23216
Alpha_737204        GGTTAAAAACAAATGTGTCAATTTCAACCTCAATGGTTTAACAGGCACAGGTGTTCTTAC   23202
Delta_2183060       GGTTAAAAACAAATGTGTCAATTTCAACCTCAATGGTTTAACAGGCACAGGTGTTCTTAC   23203
Omicron_7869197     GGTTAAAAACAAATGTGTCAATTTCAACCTCAATGGTTTAAAAGGCACAGGTGTTCTTAC   23188
                    ***************************************** ******************

w.t._745046         TGAGTCTAACAAAAAGTTTCTGCCTTTCCAACAATTTGGCAGAGACATTGCTGACACTAC   23276
Alpha_737204        TGAGTCTAACAAAAAGTTTCTGCCTTTCCAACAATTTGGCAGAGACATTGATGACACTAC   23262
Delta_2183060       TGAGTCTAACAAAAAGTTTCTGCCTTTCCAACAATTTGGCAGAGACATTGCTGACACTAC   23263
Omicron_7869197     TGAGTCTAACAAAAAGTTTCTGCCTTTCCAACAATTTGGCAGAGACATTGCTGACACTAC   23248
                    ************************************************** *********

w.t._745046         TGATGCTGTCCGTGATCCACAGACACTTGAGATTCTTGACATTACACCATGTTCTTTTGG   23336
Alpha_737204        TGATGCTGTCCGTGATCCACAGACACTTGAGATTCTTGACATTACACCATGTTCTTTTGG   23322
Delta_2183060       TGATGCTGTCCGTGATCCACAGACACTTGAGATTCTTGACATTACACCATGTTCTTTTGG   23323
Omicron_7869197     TGATGCTGTCCGTGATCCACAGACACTTGAGATTCTTGACATTACACCATGTTCTTTTGG   23308
                    ************************************************************

w.t._745046         TGGTGTCAGTGTTATAACACCAGGAACAAATACCTCTAACCAGGTTGCTGTTCTTTATCA   23396
Alpha_737204        TGGTGTCAGTGTTATAACACCAGGAACAAATACCTCTAACCAGGTTGCTGTTCTTTATCA   23382
Delta_2183060       TGGTGTCAGTGTTATAACACCAGGAACAAATACCTCTAACCAGGTTGCTGTTCTTTATCA   23383
Omicron_7869197     TGGTGTCAGTGTTATAACACCAGGAACAAATACCTCTAACCAGGTTGCTGTTCTTTATCA   23368
                    ************************************************************
```

```
w.t._745046         GGGTGTTAACTGCACAGAAGTCCCTGTTGCTATTCATGCAGATCAACTTACTCCTACTTG   23456
Alpha_737204        GGGTGTTAACTGCACAGAAGTCCCTGTTGCTATTCATGCAGATCAACTTACTCCTACTTG   23442
Delta_2183060       GGGTGTTAACTGCACAGAAGTCCCTGTTGCTATTCATGCAGATCAACTTACTCCTACTTG   23443
Omicron_7869197     GGGTGTTAACTGCACAGAAGTCCCTGTTGCTATTCATGCAGATCAACTTACTCCTACTTG   23428
                    ************************************************************

w.t._745046         GCGTGTTTATTCTACAGGTTCTAATGTTTTTCAAACACGTGCAGGCTGTTTAATAGGGGC   23516
Alpha_737204        GCGTGTTTATTCTACAGGTTCTAATGTTTTTCAAACACGTGCAGGCTGTTTAATAGGGGC   23502
Delta_2183060       GCGTGTTTATTCTACAGGTTCTAATGTTTTTCAAACACGTGCAGGCTGTTTAATAGGGGC   23503
Omicron_7869197     GCGTGTTTATTCTACAGGTTCTAATGTTTTTCAAACACGTGCAGGCTGTTTAATAGGGGC   23488
                    ************************************************************

w.t._745046         TGAACATGTCAACAACTCATATGAGTGTGACATACCCATTGGTGCAGGTATATGCGCTAG   23576
Alpha_737204        TGAACATGTCAACAACTCATATGAGTGTGACATACCCATTGGTGCAGGTATATGCGCTAG   23562
Delta_2183060       TGAACATGTCAACAACTCATATGAGTGTGACATACCCATTGGTGCAGGTATATGCGCTAG   23563
Omicron_7869197     TGAATATGTCAACAACTCATATGAGTGTGACATACCCATTGGTGCAGGTATATGCGCTAG   23548
                    **** *******************************************************

w.t._745046         TTATCAGACTCAGACTAATTCTCCTCGGCGGGCACGTAGTGTAGCTAGTCAATCCATCAT   23636
Alpha_737204        TTATCAGACTCAGACTAATTCTCATCGGCGGGCACGTAGTGTAGCTAGTCAATCCATCAT   23622
Delta_2183060       TTATCAGACTCAGACTAATTCTCGTCGGCGGGCACGTAGTGTAGCTAGTCAATCCATCAT   23623
Omicron_7869197     TTATCAGACTCAGACTAAGTCTCATCGGCGGGCACGTAGTGTAGCTAGTCAATCCATCAT   23608
                    ****************** **** ************************************

w.t._745046         TGCCTACACTATGTCACTTGGTGCAGAAAATTCAGTTGCTTACTCTAATAACTCTATTGC   23696
Alpha_737204        TGCCTACACTATGTCACTTGGTGCAGAAAATTCAGTTGCTTACTCTAATAACTCTATTGC   23682
Delta_2183060       TGCCTACACTATGTCACTTGGTGCAGAAAATTCAGTTGCTTACTCTAATAACTCTATTGC   23683
Omicron_7869197     TGCCTACACTATGTCACTTGGTGCAGAAAATTCAGTTGCTTACTCTAATAACTCTATTGC   23668
                    ************************************************************

w.t._745046         CATACCCACAAATTTTACTATTAGTGTTACCACAGAAATTCTACCAGTGTCTATGACCAA   23756
Alpha_737204        CATACCCATAAATTTTACTATTAGTGTTACCACAGAAATTCTACCAGTGTCTATGACCAA   23742
Delta_2183060       CATACCCACAAATTTTACTATTAGTGTTACCACAGAAATTCTACCAGTGTCTATGACCAA   23743
Omicron_7869197     CATACCCACAAATTTTACTATTAGTGTTACCACAGAAATTCTACCAGTGTCTATGACCAA   23728
                    ******** ***************************************************

w.t._745046         GACATCAGTAGATTGTACAATGTACATTTGTGGTGATTCAACTGAATGCAGCAATCTTTT   23816
Alpha_737204        GACATCAGTAGATTGTACAATGTACATTTGTGGTGATTCAACTGAATGCAGCAATCTTTT   23802
Delta_2183060       GACATCAGTAGATTGTACAATGTACATTTGTGGTGATTCAACTGAATGCAGCAATCTTTT   23803
Omicron_7869197     GACATCAGTAGATTGTACAATGTACATTTGTGGTGATTCAACTGAATGCAGCAATCTTTT   23788
                    ************************************************************

w.t._745046         GTTGCAATATGGCAGTTTTTGTACACAATTAAACCGTGCTTTAACTGGAATAGCTGTTGA   23876
Alpha_737204        GTTGCAATATGGCAGTTTTTGTACACAATTAAACCGTGCTTTAACTGGAATAGCTGTTGA   23862
Delta_2183060       GTTGCAATATGGCAGTTTTTGTACACAATTAAACCGTGCTTTAACTGGAATAGCTGTTGA   23863
Omicron_7869197     GTTGCAATATGGCAGTTTTTGTACACAATTAAAACGTGCTTTAACTGGAATAGCTGTTGA   23848
                    ********************************* **************************

w.t._745046         ACAAGACAAAAACACCCAAGAAGTTTTTGCACAAGTCAAACAAATTTACAAAACACCACC   23936
Alpha_737204        ACAAGACAAAAACACCCAAGAAGTTTTTGCACAAGTCAAACAAATTTACAAAACACCACC   23922
Delta_2183060       ACAAGACAAAAACACCCAAGAAGTTTTTGCACAAGTCAAACAAATTTACAAAACACCACC   23923
Omicron_7869197     ACAAGACAAAAACACCCAAGAAGTTTTTGCACAAGTCAAACAAATTTACAAAACACCACC   23908
                    ************************************************************

w.t._745046         AATTAAAGATTTTGGTGGTTTTAATTTTTCACAAATATTACCAGATCCATCAAAACCAAG   23996
Alpha_737204        AATTAAAGATTTTGGTGGTTTTAATTTTTCACAAATATTACCAGATCCATCAAAACCAAG   23982
Delta_2183060       AATTAAAGATTTTGGTGGTTTTAATTTTTCACAAATATTACCAGATCCATCAAAACCAAG   23983
Omicron_7869197     AATTAAATATTTTGGTGGTTTTAATTTTTCACAAATATTACCAGATCCATCAAAACCAAG   23968
                    ******* ****************************************************
```

```
w.t._745046         CAAGAGGTCATTTATTGAAGATCTACTTTTCAACAAAGTGACACTTGCAGATGCTGGCTT   24056
Alpha_737204        CAAGAGGTCATTTATTGAAGATCTACTTTTCAACAAAGTGACACTTGCAGATGCTGGCTT   24042
Delta_2183060       CAAGAGGTCATTTATTGAAGATCTACTTTTCAACAAAGTGACACTTGCAGATGCTGGCTT   24043
Omicron_7869197     CAAGAGGTCATTTATTGAAGATCTACTTTTCAACAAAGTGACACTTGCAGATGCTGGCTT   24028
                    ************************************************************

w.t._745046         CATCAAACAATATGGTGATTGCCTTGGTGATATTGCTGCTAGAGACCTCATTTGTGCACA   24116
Alpha_737204        CATCAAACAATATGGTGATTGCCTTGGTGATATTGCTGCTAGAGACCTCATTTGTGCACA   24102
Delta_2183060       CATCAAACAATATGGTGATTGCCTTGGTGATATTGCTGCTAGAGACCTCATTTGTGCACA   24103
Omicron_7869197     CATCAAACAATATGGTGATTGCCTTGGTGATATTGCTGCTAGAGACCTCATTTGTGCACA   24088
                    ************************************************************

w.t._745046         AAAGTTTAACGGCCTTACTGTTTTGCCACCTTTGCTCACAGATGAAATGATTGCTCAATA   24176
Alpha_737204        AAAGTTTAACGGCCTTACTGTTTTGCCACCTTTGCTCACAGATGAAATGATTGCTCAATA   24162
Delta_2183060       AAAGTTTAACGGCCTTACTGTTTTGCCACCTTTGCTCACAGATGAAATGATTGCTCAATA   24163
Omicron_7869197     AAAGTTTAAAGGCCTTACTGTTTTGCCACCTTTGCTCACAGATGAAATGATTGCTCAATA   24148
                    ********* **************************************************

w.t._745046         CACCTCTGCACTGTTAGCGGGTACAATCACCTCTGGTTGGACCTTTGGTGCAGGTGCTGC   24236
Alpha_737204        CACCTCTGCACTGTTAGCGGGTACAATCACCTCTGGTTGGACCTTTGGTGCAGGTGCTGC   24222
Delta_2183060       CACCTCTGCACTGTTAGCGGGTACAATCACCTCTGGTTGGACCTTTGGTGCAGGTGCTGC   24223
Omicron_7869197     CACCTCTGCACTGTTAGCGGGTACAATCACCTCTGGTTGGACCTTTGGTGCAGGTGCTGC   24208
                    ************************************************************

w.t._745046         ATTACAAATACCATTTGCTATGCAAATGGCTTATAGGTTTAATGGTATTGGAGTTACACA   24296
Alpha_737204        ATTACAAATACCATTTGCTATGCAAATGGCTTATAGGTTTAATGGTATTGGAGTTACACA   24282
Delta_2183060       ATTACAAATACCATTTGCTATGCAAATGGCTTATAGGTTTAATGGTATTGGAGTTACACA   24283
Omicron_7869197     ATTACAAATACCATTTGCTATGCAAATGGCTTATAGGTTTAATGGTATTGGAGTTACACA   24268
                    ************************************************************

w.t._745046         GAATGTTCTCTATGAGAACCAAAAATTGATTGCCAACCAATTTAATAGTGCTATTGGCAA   24356
Alpha_737204        GAATGTTCTCTATGAGAACCAAAAATTGATTGCCAACCAATTTAATAGTGCTATTGGCAA   24342
Delta_2183060       GAATGTTCTCTATGAGAACCAAAAATTGATTGCCAACCAATTTAATAGTGCTATTGGCAA   24343
Omicron_7869197     GAATGTTCTCTATGAGAACCAAAAATTGATTGCCAACCAATTTAATAGTGCTATTGGCAA   24328
                    ************************************************************

w.t._745046         AATTCAAGACTCACTTTCCTCCACAGCAAGTGCACTTGGAAAACCTCAAGATGTGGTCAA   24416
Alpha_737204        AATTCAAGACTCACTTTCCTCCACAGCAAGTGCACTTGGAAAACCTCAAGATGTGGTCAA   24402
Delta_2183060       AATTCAAGACTCACTTTCCTCCACAGCAAGTGCACTTGGAAAACCTCAAAATGTGGTCAA   24403
Omicron_7869197     AATTCAAGACTCACTTTCCTCCACAGCAAGTGCACTTGGAAAACCTCAAGATGTGGTCAA   24388
                    ************************************************* **********

w.t._745046         CCAAAATGCACAAGCTTTAAACACGCTTGTTAAACAACTTAGCTCCAATTTTGGTGCAAT   24476
Alpha_737204        CCAAAATGCACAAGCTTTAAACACGCTTGTTAAACAACTTAGCTCCAATTTTGGTGCAAT   24462
Delta_2183060       CCAAAATGCACAAGCTTTAAACACGCTTGTTAAACAACTTAGCTCCAATTTTGGTGCAAT   24463
Omicron_7869197     CCATAATGCACAAGCTTTAAACACGCTTGTTAAACAACTTAGCTCCAAATTTGGTGCAAT   24448
                    *** ******************************************** ***********

w.t._745046         TTCAAGTGTTTTAAATGATATCCTTTCACGTCTTGACAAAGTTGAGGCTGAAGTGCAAAT   24536
Alpha_737204        TTCAAGTGTTTTAAATGATATCCTTGCACGTCTTGACAAAGTTGAGGCTGAAGTGCAAAT   24522
Delta_2183060       TTCAAGTGTTTTAAATGATATCCTTTCACGTCTTGACAAAGTTGAGGCTGAAGTGCAAAT   24523
Omicron_7869197     TTCAAGTGTTTTAAATGATATCTTTTCACGTCTTGACAAAGTTGAGGCTGAAGTGCAAAT   24508
                    ********************** ** **********************************

w.t._745046         TGATAGGTTGATCACAGGCAGACCTCAAAGTTTGCAGACATATGTGACTCAACAATTAAT   24596
Alpha_737204        TGATAGGTTGATCACAGGCAGACCTCAAAGTTTGCAGACATATGTGACTCAACAATTAAT   24582
Delta_2183060       TGATAGGTTGATCACAGGCAGACCTCAAAGTTTGCAGACATATGTGACTCAACAATTAAT   24583
Omicron_7869197     TGATAGGTTGATCACAGGCAGACCTCAAAGTTTGCAGACATATGTGACTCAACAATTAAT   24568
                    ************************************************************
```

```
w.t._745046         TAGAGCTGCAGAAATCAGAGCCTCTGCTAATCTTGCTGCTACTAAAATGTCAGAGTGTGT   24656
Alpha_737204        TAGAGCTGCAGAAATCAGAGCCTCTGCTAATCTTGCTGCTACTAAAATGTCAGAGTGTGT   24642
Delta_2183060       TAGAGCTGCAGAAATCAGAGCCTCTGCTAATCTTGCTGCTACTAAAATGTCAGAGTGTGT   24643
Omicron_7869197     TAGAGCTGCAGAAATCAGAGCCTCTGCTAATCTTGCTGCTACTAAAATGTCAGAGTGTGT   24628
                    ************************************************************

w.t._745046         ACTTGGACAATCAAAAAGAGTTGATTTTTGTGGAAAGGGCTATCATCTTATGTCCCTCCC   24716
Alpha_737204        ACTTGGACAATCAAAAAGAGTTGATTTTTGTGGAAAGGGCTATCATCTTATGTCCCTCCC   24702
Delta_2183060       ACTTGGACAATCAAAAAGAGTTGATTTTTGTGGAAAGGGCTATCATCTTATGTCCCTCCC   24703
Omicron_7869197     ACTTGGACAATCAAAAAGAGTTGATTTTTGTGGAAAGGGCTATCATCTTATGTCCCTCCC   24688
                    ************************************************************

w.t._745046         TCAGTCAGCACCTCATGGTGTAGTCCTCTTGCATGTGACTTATGTCCCTGCACAAGAAAA   24776
Alpha_737204        TCAGTCAGCACCTCATGGTGTAGTCCTCTTGCATGTGACTTATGTCCCTGCACAAGAAAA   24762
Delta_2183060       TCAGTCAGCACCTCATGGTGTAGTTTTCTTGCATGTGACTTATGTCCCTGCACAAGAAAA   24763
Omicron_7869197     TCAGTCAGCACCTCATGGTGTAGTCCTCTTGCATGTGACTTATGTCCCTGCACAAGAAAA   24748
                    ************************  **********************************

w.t._745046         GAACCTCACAACTGCTCCTGCCATTTGTCATGATGGAAAAGCACACTTTCCTCGTGAAGG   24836
Alpha_737204        GAACCTCACAACTGCTCCTGCCATTTGTCATGATGGAAAAGCACACTTTCCTCGTGAAGG   24822
Delta_2183060       GAACCTCACAACTGCTCCTGCCATTTGTCATGATGGAAAAGCACACTTTCCTCGTGAAGG   24823
Omicron_7869197     GAACCTCACAACTGCTCCTGCCATTTGTCATGATGGAAAAGCACACTTTCCTCGTGAAGG   24808
                    ************************************************************

w.t._745046         TGTCTTTGTTTCAAATGGCACACACTGGTTTGTAACACAAAGGAATTTTTATGAACCACA   24896
Alpha_737204        TGTCTTTGTTTCAAATGGCACACACTGGTTTGTAACACAAAGGAATTTTTATGAACCACA   24882
Delta_2183060       TGTCTTTGTTTCAAATGGCACACACTGGTTTGTAACACAAAGGAATTTTTATGAACCACA   24883
Omicron_7869197     TGTCTTTGTTTCAAATGGCACACACTGGTTTGTAACACAAAGGAATTTTTATGAACCACA   24868
                    ************************************************************

w.t._745046         AATCATTACTACAGACAACACATTTGTGTCTGGTAACTGTGATGTTGTAATAGGAATTGT   24956
Alpha_737204        AATCATTACTACACACAACACATTTGTGTCTGGTAACTGTGATGTTGTAATAGGAATTGT   24942
Delta_2183060       AATCATTACTACAGACAACACATTTGTGTCTGGTAACTGTGATGTTGTAATAGGAATTGT   24943
Omicron_7869197     AATCATTACTACAGACAACACATTTGTGTCTGGTAACTGTGATGTTGTAATAGGAATTGT   24928
                    ************* **********************************************

w.t._745046         CAACAACACAGTTTATGATCCTTTGCAACCTGAATTAGACTCATTCAAGGAGGAGTTAGA   25016
Alpha_737204        CAACAACACAGTTTATGATCCTTTGCAACCTGAATTAGACTCATTCAAGGAGGAGTTAGA   25002
Delta_2183060       CAACAACACAGTTTATGATCCTTTGCAACCTGAATTAGACTCATTCAAGGAGGAGTTAGA   25003
Omicron_7869197     CAACAACACAGTTTATGATCCTTTGCAACCTGAATTAGATTCATTCAAGGAGGAGTTAGA   24988
                    *************************************** ********************

w.t._745046         TAAATATTTTAAGAATCATACATCACCAGATGTTGATTTAGGTGACATCTCTGGCATTAA   25076
Alpha_737204        TAAATATTTTAAGAATCATACATCACCAGATGTTGATTTAGGTGACATCTCTGGCATTAA   25062
Delta_2183060       TAAATATTTTAAGAATCATACATCACCAGATGTTGATTTAGGTGACATCTCTGGCATTAA   25063
Omicron_7869197     TAAATATTTTAAGAATCATACATCACCAGATGTTGATTTAGGTGACATCTCTGGCATTAA   25048
                    ************************************************************

w.t._745046         TGCCTCAGTTGTAAACATTCAAAAAGAAATTGACCGCCTCAATGAGGTTGCCAAGAATTT   25136
Alpha_737204        TGCCTCAGTTGTAAACATTCAAAAAGAAATTGACCGCCTCAATGAGGTTGCCAAGAATTT   25122
Delta_2183060       TGCCTCAGTTGTAAACATTCAAAAAGAAATTGACCGCCTCAATGAGGTTGCCAAGAATTT   25123
Omicron_7869197     TGCCTCAGTTGTAAACATTCAAAAAGAAATTGACCGCCTCAATGAGGTTGCCAAGAATTT   25108
                    ************************************************************

w.t._745046         AAATGAATCTCTCATCGATCTCCAAGAACTTGGAAAGTATGAGCAGTATATAAAATGGCC   25196
Alpha_737204        AAATGAATCTCTCATCGATCTCCAAGAACTTGGAAAGTATGAGCAGTATATAAAATGGCC   25182
Delta_2183060       AAATGAATCTCTCATCGATCTCCAAGAACTTGGAAAGTATGAGCAGTATATAAAATGGCC   25183
Omicron_7869197     AAATGAATCTCTCATCGATCTCCAAGAACTTGGAAAGTATGAGCAGTATATAAAATGGCC   25168
                    ************************************************************
```

```
w.t._745046         ATGGTACATTTGGCTAGGTTTTATAGCTGGCTTGATTGCCATAGTAATGGTGACAATTAT   25256
Alpha_737204        ATGGTACATTTGGCTAGGTTTTATAGCTGGCTTGATTGCCATAGTAATGGTGACAATTAT   25242
Delta_2183060       ATGGTACATTTGGCTAGGTTTTATAGCTGGCTTGATTGCCATAGTAATGGTGACAATTAT   25243
Omicron_7869197     ATGGTACATTTGGCTAGGTTTTATAGCTGGCTTGATTGCCATAGTAATGGTGACAATTAT   25228
                    ************************************************************

w.t._745046         GCTTTGCTGTATGACCAGTTGCTGTAGTTGTCTCAAGGGCTGTTGTTCTTGTGGATCCTG   25316
Alpha_737204        GCTTTGCTGTATGACCAGTTGCTGTAGTTGTCTCAAGGGCTGTTGTTCTTGTGGATCCTG   25302
Delta_2183060       GCTTTGCTGTATGACCAGTTGCTGTAGTTGTCTCAAGGGCTGTTGTTCTTGTGGATCCTG   25303
Omicron_7869197     GCTTTGCTGTATGACCAGTTGCTGTAGTTGTCTCAAGGGCTGTTGTTCTTGTGGATCCTG   25288
                    ************************************************************

w.t._745046         CTGCAAATTTGATGAAGACGACTCTGAGCCAGTGCTCAAAGGAGTCAAATTACATTACAC   25376
Alpha_737204        CTGCAAATTTGATGAAGACGACTCTGAGCCAGTGCTCAAAGGAGTCAAATTACATTACAC   25362
Delta_2183060       CTGCAAATTTGATGAAGACGACTCTGAGCCAGTGCTCAAAGGAGTCAAATTACATTACAC   25363
Omicron_7869197     CTGCAAATTTGATGAAGACGACTCTGAGCCAGTGCTCAAAGGAGTCAAATTACATTACAC   25348
                    ************************************************************

w.t._745046         ATAAACGAACTTATGGATTTGTTTATGAGAATCCTCACAATTGGAACTGTAACTTTGAAG   25436
Alpha_737204        ATAAACGAACTTATGGATTTGTTTATGAGAATCCTCACAATTGGAACTGTAACTTTTAAG   25422
Delta_2183060       ATAAACGAACTTATGGATTTGTTTATGAGAATCCTCACAATTGGAACTGTAACTTTGAAG   25423
Omicron_7869197     ATAAACGAACTTATGGATTTGTTTATGAGAATCCTCACAATTGGAACTGTAACTTTGAAG   25408
                    ******************************************************** ***

w.t._745046         CAAGGTGAAATCAAGGATGCTACTCCCTCAGATTTTGTTCGCGCTACTGCAACGATACCG   25496
Alpha_737204        CAAGGTGAAATCAAGGATGCTACTCCCTCAGATTTTGTTCGCGCTACTGCAACGATACCG   25482
Delta_2183060       CAAGGTGAAATCAAGGATGCTACTCCTTTAGATTTTGTTCGCGCTACTGCAACGATACCG   25483
Omicron_7869197     CAAGGTGAAATCAAGGATGCTACTCCCTCAGATTTTGTTCGCGCTACTGCAACGATACCG   25468
                    **************************** *******************************

w.t._745046         ATACAAGCCTCACTCCCTTTCGGATGGCTTATTGTTGGCGTTGCACCTCTTGCTGTTTTT   25556
Alpha_737204        ATACAAGCCTCACTCCCTTTCGGATGGCTTATTGTTGGCGTTGCACCTCTTGCTGTTTTT   25542
Delta_2183060       ATACAAGCCTCACTCCCTTTCGGATGGCTTATTGTTGGCGTTGCACCTCTTGCTGTTTTT   25543
Omicron_7869197     ATACAAGCCTCACTCCCTTTCGGATGGCTTATTGTTGGCGTTGCACCTCTTGCTGTTTTT   25528
                    ************************************************************

w.t._745046         CAGAGCGCCTCCAAAATCATAACCCTCAAAAAGAGATGGCAACTAGCACTCTCCAAGGGT   25616
Alpha_737204        CAGAGCGCCTCCAAAATCATAACCCTCAAAAAGAGATGGCAACTAGCACTCTCCAAGGGT   25602
Delta_2183060       CAGAGCGCCTCCAAAATCATAACCCTCAAAAAGAGATGGCAACTAGCACTCTCCAAGGGT   25603
Omicron_7869197     CAGAGCGCCTCCAAAATCATAACTCTCAAAAAGAGATGGCAACTAGCACTCTCCAAGGGT   25588
                    *********************** ************************************

w.t._745046         GTTCACTTTGTTTGCAACTTGCTGTTGTTGTTTGTAACAGTTTACTCACACCTTTTGCTC   25676
Alpha_737204        GTTCACTTTGTTTGCAACTTGCTGTTGTTGTTTGTAACAGTTTACTCACACCTTTTGCTC   25662
Delta_2183060       GTTCACTTTGTTTGCAACTTGCTGTTGTTGTTTGTAACAGTTTACTCACACCTTTTGCTC   25663
Omicron_7869197     GTTCACTTTGTTTGCAACTTGCTGTTGTTGTTTGTAACAGTTTACTCACACCTTTTGCTC   25648
                    ************************************************************

w.t._745046         GTTGCTGCTGGCCTTGAAGCCCCTTTTCTCTATCTTTATGCTTTAGTCTACCTCTTGCAG   25736
Alpha_737204        GTTGCTGCTGGCCTTGAAGCCCCTTTTCTCTATCTTTATGCTTTAGTCTACCTCTTGCAG   25722
Delta_2183060       GTTGCTGCTGGCCTTGAAGCCCCTTTTCTCTATCTTTATGCTTTAGTCTACCTCTTGCAG   25723
Omicron_7869197     GTTGCTGCTGGCCTTGAAGCCCCTTTTCTCTATCTTTATGCTTTAGTCTACCTCTTGCAG   25708
                    ************************************************************

w.t._745046         AGTATAAACTTTGTAAGAATAATAATGAGGCTTTGGCTTTGCTGTAAATGCCGTTCCAAA   25796
Alpha_737204        AGTATAAACTTTGTAAGAATAATAATGAGGCTTTGGCTTTGCTGGAAATGCCGTTCCAAA   25782
Delta_2183060       AGTATAAACTTTGTAAGAATAATAATGAGGCTTTGGCTTTGCTGGAAATGCCGTTCCAAA   25783
Omicron_7869197     AGTATAAACTTTGTAAGAATAATAATGAGGCTTTGGCTTTGCTGGAAATGCCGTTCCAAA   25768
                    ******************************************** ***************
```

```
w.t._745046         AACCCATTACTTTATGATGCCAACTATTTTCTTTGCTGGCATACTAATTGTTACGACTAT   25856
Alpha_737204        AACCCATTACTTTATGATGCCAACTATTTTCTTTGCTGGCATACTAATTGTTACGACTAT   25842
Delta_2183060       AACCCATTACTTTATGATGCCAACTATTTTCTTTGCTGGCATACTAATTGTTACGACTAT   25843
Omicron_7869197     AACCCATTACTTTATGATGCCAACTATTTTCTTTGCTGGCATACTAATTGTTACGACTAT   25828
                    ************************************************************

w.t._745046         TGTATACCTTACAATAGTGTAACCTCTTCAATTGTCATTACCTCAGGTGATGGCACAACA   25916
Alpha_737204        TGTATACCTTACAATAGTGTAACCTCTTCAATTGTCATTACCTCAGGTGATGGCACAACA   25902
Delta_2183060       TGTATACCTTACAATAGTGTAACCTCTTCAATTGTCATTACCTCAGGTGATGGCACAACA   25903
Omicron_7869197     TGTATACCTTACAATAGTGTAACCTCTTCAATTGTCATTACCTCAGGTGATGGCACAACA   25888
                    ************************************************************

w.t._745046         AGTCCTATTTCTGAACATGACTACCAGATTGGTGGTTATACTGAAAAATGGGAATCTGGA   25976
Alpha_737204        AGTCCTATTTCTGAACATGACTACCAGATTGGTGGTTATACTGAAAAATGGGAATCTGGA   25962
Delta_2183060       AGTCCTATTTCTGAACATGACTACCAGATTGGTGGTTATACTGAAAAATGGGAATCTGGA   25963
Omicron_7869197     AGTCCTATTTCTGAACATGACTACCAGATTGGTGGTTATACTGAAAAATGGGAATCTGGA   25948
                    ************************************************************

w.t._745046         GTAAAAGACTGTGTTGTATTACACAGTTACCTCACCTCAGACTATTACCAGCTGTACTCA   26036
Alpha_737204        GTAAAAGACTGTGTTGTATTACACAGTTACCTCACCTCAGACTATTACCAGCTGTACTCA   26022
Delta_2183060       GTAAAAGACTGTGTTGTATTACACAGTTACCTCACCTCAGACTATTACCAGCTGTACTCA   26023
Omicron_7869197     GTAAAAGACTGTGTTGTATTACACAGTTACCTCACCTCAGACTATTACCAGCTGTACTCA   26008
                    ************************************************************

w.t._745046         ACTCAATTGAGTACAGACACTGGTGTTGAACATGTTACCCTCTTCATCTACAATAAAATT   26096
Alpha_737204        ACTCAATTGAGTACAGACACTGGTGTTGAACATGTTACCCTCTTCATCTACAATAAAATT   26082
Delta_2183060       ACTCAATTGAGTACAGACACTGGTGTTGAACATGTTACCCTCTTCATCTACAATAAAATT   26083
Omicron_7869197     ACTCAATTGAGTACAGACACTGGTGTTGAACATGTTACCCTCTTCATCTACAATAAAATT   26068
                    ************************************************************

w.t._745046         GTTGATGAGCCTGAAGAACATGTCCAAATTCACACAATCGACGGTTCATCCGGAGTTGTT   26156
Alpha_737204        GTTGATGAGCCTGAAGAACATGTCCAAATTCACACAATCGACGGTTCATCCGGAGTTGTT   26142
Delta_2183060       GTTGATGAGCCTGAAGAACATGTCCAAATTCACACAATCGACGGTTCATCCGGAGTTGTT   26143
Omicron_7869197     GTTGATGAGCCTGAAGAACATGTCCAAATTCACACAATCGACGGTTCATCCGGAGTTGTT   26128
                    ************************************************************

w.t._745046         AATCCAGTAATGGAACCAATTTATGATGAACCGACGACGACTACTAGCGTGCCTTTGTAA   26216
Alpha_737204        AATCCAGTAATGGAACCAATTTATGATGAACCGACGACGACTACTAGCGTGCCTTTGTAA   26202
Delta_2183060       AATCCAGTAATGGAACCAATTTATGATGAACCGACGACGACTACTAGCGTGCCTTTGTAA   26203
Omicron_7869197     AATCCAGTAATGGAACCAATTTATGATGAACCGACGACGACTACTAGCGTGCCTTTGTAA   26188
                    ************************************************************

w.t._745046         GCACAAGCTGATGAGTACGAACTTATGTACTCATTCGTTTCGGAAGAGACAGGTACGTTA   26276
Alpha_737204        GCACAAGCTGATGAGTACGAACTTATGTACTCATTCGTTTCGGAAGAGACAGGTACGTTA   26262
Delta_2183060       GCACAAGCTGATGAGTACGAACTTATGTACTCATTCGTTTCGGAAGAGACAGGTACGTTA   26263
Omicron_7869197     GCACAAGCTGATGAGTACGAACTTATGTACTCATTCGTTTCGGAAGAGATAGGTACGTTA   26248
                    ************************************************* **********

w.t._745046         ATAGTTAATAGCGTACCTCTTTTTCTTGCTTTCGTGGTATTCTTGCTAGTTACACTAGCC   26336
Alpha_737204        ATAGTTAATAGCGTACCTCTTTTTCTTGCTTTCGTGGTATTCTTGCTAGTTACACTAGCC   26322
Delta_2183060       ATAGTTAATAGCGTACCTCTTTTTCTTGCTTTCGTGGTATTCTTGCTAGTTACACTAGCC   26323
Omicron_7869197     ATAGTTAATAGCGTACCTCTTTTTCTTGCTTTCGTGGTATTCTTGCTAGTTACACTAGCC   26308
                    ************************************************************

w.t._745046         ATCCTTACTGCGCCTCGATTGTGTGCGTACTGCTGCAATATTGTTAACGTGAGTCTTGTA   26396
Alpha_737204        ATCCTTACTGCGCCTCGATTGTGTGCGTACTGCTGCAATATTGTTAACGTGAGTCTTGTA   26382
Delta_2183060       ATCCTTACTGCGCCTCGATTGTGTGCGTACTGCTGCAATATTGTTAACGTGAGTCTTGTA   26383
Omicron_7869197     ATCCTTACTGCGCCTCGATTGTGTGCGTACTGCTGCAATATTGTTAACGTGAGTCTTGTA   26368
                    ************************************************************
```

```
w.t._745046        AAACCCTCTTTTTACGTTTACTCTCGTGTTAAAAATCTGAATTCCTCTAGAGTTCCTGAT   26456
Alpha_737204       AAACCCTCTTTTTACGTTTACTCTCGTGTTAAAAATCTGAATTCCTCTAGAGTTCCTGAT   26442
Delta_2183060      AAACCCTCTTTTTACGTTTACTCTCGTGTTAAAAATCTGAATTCCTCTAGAGTTCCTGAT   26443
Omicron_7869197    AAACCCTCTTTTTACGTTTACTCTCGTGTTAAAAATCTGAATTCCTCTAGAGTTCCTGAT   26428
                   ************************************************************

w.t._745046        CCTCTGGTCTAAACGAACTAAATATTATATTAGTTTTTCTGTTTGGAACTTTAATTTTAG   26516
Alpha_737204       CCTCTGGTCTAAACGAACTAAATATTATATTAGTTTTTCTGTTTGGAACTTTAATTTTAG   26502
Delta_2183060      CCTCTGGTCTAAACGAACTAAATATTATATTAGTTTTTCTGTTTGGAACTTTAATTTTAG   26503
Omicron_7869197    CCTCTGGTCTAAACGAACTAAATATTATATTAGTTTTTCTGTTTGGAACTTTAATTTTAG   26488
                   ************************************************************

w.t._745046        CCATGGCAGATTCCAACGGTACTATTACCGTTGAAGAGCTTAAAAAGCTCCTTGAACAAT   26576
Alpha_737204       CCATGGCAGATTCCAACGGTACTATTACCGTTGAAGAGCTTAAAAAGCTCCTTGAACAAT   26562
Delta_2183060      CCATGGCAGATTCCAACGGTACTATTACCGTTGAAGAGCTTAAAAAGCTCCTTGAACAAT   26563
Omicron_7869197    CCATGGCAGRTTCCAACGGTACTATTACCGTTGAAGAGCTTAAAAAGCTCCTTGAAGAAT   26548
                   ********* ********************************************** ***

w.t._745046        GGAACCTAGTAATAGGTTTCCTATTCCTTACATGGATTTGTCCTCTACAATTTGCCTATG   26636
Alpha_737204       GGAACCTAGTAATAGGTTTCCTATTCCTTACATGGATTTGTCCTCTACAATTTGCCTATG   26622
Delta_2183060      GGAACCTAGTAATAGGTTTCCTATTCCTTACATGGATTTGTCCTCTACAATTTGCCTATG   26623
Omicron_7869197    GGAACCTAGTAATAGGTTTCCTATTCCTTACATGGATTTGTCCTCTACAATTTGCCTATG   26608
                   ************************************************************

w.t._745046        CCAACAGGAATAGGTTTTTGTATATAATTAAGTTAATTTTCCTCTGGCTGTTATGGCCAG   26696
Alpha_737204       CCAACAGGAATAGGTTTTTGTATATAATTAAGTTAATTTTCCTCTGGCTGTTATGGCCAG   26682
Delta_2183060      CCAACAGGAATAGGTTTTTGTATATAATTAAGTTAATTTTCCTCTGGCTGTTATGGCCAG   26683
Omicron_7869197    CCAACAGGAATAGGTTTTTGTATATAATTAAGTTAATTTTCCTCTGGCTGTTATGGCCAG   26668
                   ************************************************************

w.t._745046        TAACTTTAGCTTGTTTTGTGCTTGCTGCTGTTTACAGAATAAATTGGATCACCGGTGGAA   26756
Alpha_737204       TAACTTTAGCTTGTTTTGTGCTTGCTGCTGTTTACAGAATAAATTGGATCACCGGTGGAA   26742
Delta_2183060      TAACTTTAGCTTGTTTTGTGCTTGCTGCTGTTTACAGAATAAATTGGATCACCGGTGGAA   26743
Omicron_7869197    TAACTTTAACTTGTTTTGTGCTTGCTGCTGTTTACAGAATAAATTGGATCACCGGTGGAA   26728
                   ******** ***************************************************

w.t._745046        TTGCTATCGCAATGGCTTGTCTTGTAGGCTTGATGTGGCTCAGCTACCTCATTGCCTCTT   26816
Alpha_737204       TTGCTATCGCAATGGCTTGTCTTGTAGGCTTGATGTGGCTCAGCTACCTCATTGCCTCTT   26802
Delta_2183060      TTGCTACCGCAATGGCTTGTCTTGTAGGCTTGATGTGGCTCAGCTACCTCATTGCCTCTT   26803
Omicron_7869197    TTGCTATCGCAATGGCTTGTCTTGTAGGCTTGATGTGGCTCAGCTACCTCATTGCCTCTT   26788
                   ****** *****************************************************

w.t._745046        TCAGACTGTTTGCGCGTACGCGTTCCATGTGGTCATTCAATCCAGAAACTAACATTCCTC   26876
Alpha_737204       TCAGACTGTTTGCGCGTACGCGTTCCATGTGGTCATTCAATCCAGAAACTAACATTCCTC   26862
Delta_2183060      TCAGACTGTTTGCGCGTACGCGTTCCATGTGGTCATTCAATCCAGAAACTAACATTCCTC   26863
Omicron_7869197    TCAGACTGTTTGCGCGTACGCGTTCCATGTGGTCATTCAATCCAGAAACTAACATTCCTC   26848
                   ************************************************************

w.t._745046        TCAACGTGCCACTCCATGGCACTATTCTGACCAGACCGCCTCTAGAAAGTGAACTCGTAA   26936
Alpha_737204       TCAACGTGCCACTCCATGGCACTATTCTGACCAGACCGCCTCTAGAAAGTGAACTCGTAA   26922
Delta_2183060      TCAACGTGCCACTCCATGGCACTATTCTGACCAGACCGCCTCTAGAAAGTGAACTCGTAA   26923
Omicron_7869197    TCAACGTGCCACTCCATGGCACTATTCTGACCAGACCGCCTCTAGAAAGTGAACTCGTAA   26908
                   ************************************************************

w.t._745046        TCGGAGCTGTGATCCCTCGTGGACATCCTCGTATTGCTGGACACCATCTAGGACGCTGTG   26996
Alpha_737204       TCGGAGCTGTGATCCCTCGTGGACATCCTCGTATTGCTGGACACCATCTAGGACGCTGTG   26982
Delta_2183060      TCGGAGCTGTGATCCCTCGTGGACATCCTCGTATTGCTGGACACCATCTAGGACGCTGTG   26983
Omicron_7869197    TCGGAGCTGTGATCCCTCGTGGACATCCTCGTATTGCTGGACACCATCTAGGACGCTGTG   26968
                   ************************************************************
```

```
w.t._745046        ACATCAAGGACCTGCCTAAAGAAATCACTGTTGCTACATCACGAACGCTTTCTTATTACA   27056
Alpha_737204       ACATCAAGGACCTGCCTAAAGAAATCACTGTTGCTACATCACGAACGCTTTCTTATTACA   27042
Delta_2183060      ACATCAAGGACCTGCCTAAAGAAATCACTGTTGCTACATCACGAACGCTTTCTTATTACA   27043
Omicron_7869197    ACATCAAGGACCTGCCTAAAGAAATCACTGTTGCTACATCACGAACGCTTTCTTATTACA   27028
                   ************************************************************

w.t._745046        AATTGGGAGCCTCGCAGCGTGTAGCAGGTGACTCAGGTTTTGCTGCATACAGTCGCTACA   27116
Alpha_737204       AATTGGGAGCCTCGCAGCGTGTAGCAGGTGACTCAGGTTTTGCTGCATACAGTCGCTACA   27102
Delta_2183060      AATTGGGAGCCTCGCAGCGTGTAGCAGGTGACTCAGGTTTTGCTGCATACAGTCGCTACA   27103
Omicron_7869197    AATTGGGAGCCTCGCAGCGTGTAGCAGGTGACTCAGGTTTTGCTGCATACAGTCGCTACA   27088
                   ************************************************************

w.t._745046        GGATTGGCAACTATAAATTAAACACAGACCATTCCAGTAGCAGTGACAATATTGCTTTGC   27176
Alpha_737204       GGATTGGCAACTATAAATTAAACACAGACCATTCCAGTAGCAGTGACAATATTGCTTTGC   27162
Delta_2183060      GGATTGGCAACTATAAATTAAACACAGACCATTCCAGTAGCAGTGACAATATTGCTTTGC   27163
Omicron_7869197    GGATTGGCAACTATAAATTAAACACAGACCATTCCAGTAGCAGTGACAATATTGCTTTGC   27148
                   ************************************************************

w.t._745046        TTGTACAGTAAGTGACAACAGATGTTTCATCTCGTTGACTTTCAGGTTACTATAGCAGAG   27236
Alpha_737204       TTGTACAGTAAGTGACAACAGATGTTTCATCTCGTTGACTTTCAGGTTACTATAGCAGAG   27222
Delta_2183060      TTGTACAGTAAGTGACAACAGATGTTTCATCTCGTTGACTTTCAGGTTACTATAGCAGAG   27223
Omicron_7869197    TTGTACAGTAAGTGACAACAGATGTTTCATCTCGTTGACTTTCAGGTTACTATAGCAGAG   27208
                   ************************************************************

w.t._745046        ATATTACTAATTATTATGAGGACTTTTAAAGTTTCCATTTGGAATCTTGATTACATCATA   27296
Alpha_737204       ATATTACTAATTATTATGAGGACTTTTAAAGTTTCCATTTGGAATCTTGATTACATCATA   27282
Delta_2183060      ATATTACTAATTATTATGAGGACTTTTAAAGTTTCCATTTGGAATCTTGATTACATCATA   27283
Omicron_7869197    ATATTACTAATTATTATGCGGACTTTTAAAGTTTCCATTTGGAATCTTGATTACATCATA   27268
                   ****************** *****************************************

w.t._745046        AACCTCATAATTAAAAATTTATCTAAGTCACTAACTGAGAATAAATATTCTCAATTAGAT   27356
Alpha_737204       AACCTCATAATTAAAAATTTATCTAAGTCACTAACTGAGAATAAATATTCTCAATTAGAT   27342
Delta_2183060      AACCTCATAATTAAAAATTTATCTAAGTCACTAACTGAGAATAAATATTCTCAATTAGAT   27343
Omicron_7869197    AACCTCATAATTAAAAATTTATCTAAGTCACTAACTGAGAATAAATATTCTCAATTAGAT   27328
                   ************************************************************

w.t._745046        GAAGAGCAACCAATGGAGATTGATTAAACGAACATGAAAATTATTCTTTTCTTGGCACTG   27416
Alpha_737204       GAAGAGCAACCAATGGAGATTGATTAAACGAACATGAAAATTATTCTTTTCTTGGCACTG   27402
Delta_2183060      GAAGAGCAACCAATGGAGATTGATTAAACGAACATGAAAATTATTCTTTTCTTGGCACTG   27403
Omicron_7869197    GAAGAGCAACCAATGGAGATTGATTAAACGAACATGAAAATTATTCTTTTCTTGGCACTG   27388
                   ************************************************************

w.t._745046        ATAACACTCGCTACTTGTGAGCTTTATCACTACCAAGAGTGTGTTAGAGGTACAACAGTA   27476
Alpha_737204       ATAACACTCGCTACTTGTGAGCTTTATCACTACCAAGAGTGTGTTAGAGGTACAACAGTA   27462
Delta_2183060      ATAACACTCGCTACTTGTGAGCTTTATCACTACCAAGAGTGTGTTAGAGGTACAACAGTA   27463
Omicron_7869197    ATAACACTCGCTACTTGTGAGCTTTATCACTACCAAGAGTGTGTTAGAGGTACAACAGTA   27448
                   ************************************************************

w.t._745046        CTTTTAAAAGAACCTTGCTCCTCTGGAACATACGAGGGCAATTCACCATTTCATCCTCTA   27536
Alpha_737204       CTTTTAAAAGAACCTTGCTCCTCTGGAACATACGAGGGCAATTCACCATTTCATCCTCTA   27522
Delta_2183060      CTTTTAAAAGAACCTTGCTCCTCTGGAACATACGAGGGCAATTCACCATTTCATCCTCTA   27523
Omicron_7869197    CTTTTAAAAGAACCTTGCTCCTCTGGAACATACGAGGGCAATTCACCATTTCATCCTCTA   27508
                   ************************************************************

w.t._745046        GCTGATAACAAATTTGCACTGACTTGCTTTAGCACTCAATTTGCTTTTGCTTGTCCTGAC   27596
Alpha_737204       GCTGATAACAAATTTGCACTGACTTGCTTTAGCACTCAATTTGCTTTTGCTTGTCCTGAC   27582
Delta_2183060      GCTGATAACAAATTTGCACTGACTTGCTTTAGCACTCAATTTGCTTTTGCTTGTCCTGAC   27583
Omicron_7869197    GCTGATAACAAATTTGCACTGACTTGCTTTAGCACTCAATTTGCTTTTGCTTGTCCTGAC   27568
                   ************************************************************
```

```
w.t._745046        GGCGTAAAACACGTCTATCAGTTACGTGCCAGATCAGTTTCACCTAAACTGTTCATCAGA   27656
Alpha_737204       GGCGTAAAACACGTCTATCAGTTACGTGCCAGATCAGTTTCACCTAAACTGTTCATCAGA   27642
Delta_2183060      GGCGTAAAACACGTCTATCAGTTACGTGCCAGATCAGCCTCACCTAAACTGTTCATCAGA   27643
Omicron_7869197    GGCGTAAAACACGTCTATCAGTTACGTGCCAGATCAGTTTCACCTAAACTGTTCATCAGA   27628
                   *************************************  *********************

w.t._745046        CAAGAGGAAGTTCAAGAACTTTACTCTCCAATTTTTCTTATTGTTGCGGCAATAGTGTTT   27716
Alpha_737204       CAAGAGGAAGTTCAAGAACTTTACTCTCCAATTTTTCTTATTGTTGCGGCAATAGTGTTT   27702
Delta_2183060      CAAGAGGAAGTTCAAGAACTTTACTCTCCAATTTTTCTTATTGTTGCGGCAATAGTGTTT   27703
Omicron_7869197    CAAGAGGAAGTTCAAGAACTTTACTCTCCAATTTTTCTTATTGTTGCGGCAATAGTGTTT   27688
                   ************************************************************

w.t._745046        ATAACACTTTGCCTCACACTCAAAAGAAAGACAGAATGATTGAACTTTCATTAATTGACT   27776
Alpha_737204       ATAACACTTTGCCTCACACTCAAAAGAAAGACAGAATGATTGAACTTTCATTAATTGACT   27762
Delta_2183060      ATAACACTTTGCCTCACATTCAAAAGAAAGATAGAATGATTGAACTTTCATTAATTGACT   27763
Omicron_7869197    ATAACACTTTGCCTCACACTCAAAAGAAAGACAGAATGATTGAACTTTCATTAATTGACT   27748
                   ****************** ************ ****************************

w.t._745046        TCTATTTGTGCTTTTTAGCCTTTCTGCTATTCCTTGTTTTAATTATGCTTATTATCTTTT   27836
Alpha_737204       TCTATTTGTGCTTTTTAGCCTTTCTGCTATTCCTTGTTTTAATTATGCTTATTATCTTTT   27822
Delta_2183060      TCTATTTGTGCTTTTTAGCCTTTCTGCTATTCCTTGTTTTAATTATGCTTATTATCTTTT   27823
Omicron_7869197    TCTATTTGTGCTTTTTAGCCTTTCTGTTATTCCTTGTTTTAATTATGCTTATTATCTTTT   27808
                   ************************** *********************************

w.t._745046        GGTTCTCACTTGAACTGCAAGATCATAATGAAACTTGTCACGCCTAAACGAACATGAAAT   27896
Alpha_737204       GGTTCTCACTTGAACTGCAAGATCATAATGAAACTTGTCACGCCTAAACGAACATGAAAT   27882
Delta_2183060      GGTTCTCACTTGAACTGCAAGATCATAATGAAACTTGTCACGCCTAAACGAACATGAAAT   27883
Omicron_7869197    GGTTCTCACTTGAACTGCAAGATCATAATGAAACTTGTCACGCCTAAACGAACATGAAAT   27868
                   ************************************************************

w.t._745046        TTCTTGTTTTCTTAGGAATCATCACAACTGTAGCTGCATTTCACCAAGAATGTAGTTTAC   27956
Alpha_737204       TTCTTGTTTTCTTAGGAATCATCACAACTGTAGCTGCATTTCACCAAGAATGTAGTTTAC   27942
Delta_2183060      TTCTTGTTTTCTTAGGAATCATCACAACTGTAGCTGCATTTCACCAAGAATGTAGTTTAC   27943
Omicron_7869197    TTCTTGTTTTCTTAGGAATCATCACAACTGTAGCTGCATTTCACCAAGAATGTAGTTTAC   27928
                   ************************************************************

w.t._745046        AGTCATGTACTCAACATCAACCATATGTAGTTGATGACCCGTGTCCTATTCACCTCTATT   28016
Alpha_737204       AGTCATGTACTTAACATCAACCATATGTAGTTGATGACCCGTGTCCTATTCACCTCTATT   28002
Delta_2183060      AGTCATGTACTCAACATCAACCATATGTAGTTGATGACCCTTGTCCTATTCACCTCTATT   28003
Omicron_7869197    AGTCATGTACTCAACATCAACCATATGTAGTTGATGACCCGTGTCCTATTCACCTCTATT   27988
                   *********** **************************** *******************

w.t._745046        CTAAATGGTATATTAGAGTAGGAGCTAGAAAATTAGCACCTTTAATTGAATTGTGCGTGG   28076
Alpha_737204       CTAAATGGTATATTAGAGTAGGAGCTATAAAATCAGCACCTTTAATTGAATTGTGCGTGG   28062
Delta_2183060      CTAAATGGTATATTAGAGTAGGAGCTAGAAAATCAGCACCTTTAATTGAATTGTGCGTGG   28063
Omicron_7869197    CTAAATGGTATATTAGAGTAGGAGCTAGAAAATCAGCACCTTTAATTGAATTGTGCGTGG   28048
                   *************************** ***** **************************

w.t._745046        ATGAGGCTGGTTCTAAATCACCCATTCAGTACATCGATATCGGTAATTATACAGTTTCCT   28136
Alpha_737204       ATGAGGCTGGTTCTAAATCACCCATTCAGTGCATCGATATCGGTAATTATACAGTTTCCT   28122
Delta_2183060      ATGAGGCTGGTTCTAAATCACCCATTCAGTACATCGATATCGGTAATTATACAGTTTCCT   28123
Omicron_7869197    ATGAGGCTGGTTCTAAATCACCCATTCAGTACATCGATATCGGTAATTATACAGTTTCCT   28108
                   ****************************** *****************************

w.t._745046        GTTTACCTTTTACAATTAATTGCCAGGAACCTAAATTGGGTAGTCTTGTAGTGCGTTGTT   28196
Alpha_737204       GTTTACCTTTTACAATTAATTGCCAGGAACCTAAATTGGGTAGTCTTGTAGTGCGTTGTT   28182
Delta_2183060      GTTTACCTTTTACAATTAATTGCCAGGAACCTAAATTGGGTAGTCTTGTAGTGCGTTGTT   28183
Omicron_7869197    GTTTACCTTTTACAATTAATTGCCAGGAACCTAAATTGGGTAGTCTTGTAGTGCGTTGTT   28168
                   ************************************************************
```

```
w.t._745046        CGTTCTATGAAGACTTTTTAGAGTATCATGACGTTCGTGTTGTTTTAGATTTCATCTAAA   28256
Alpha_737204       CGTTCTATGAAGACTTTTTAGAGTATCATGACGTTCGTGTTGTTTTAGATTTCATCTAAA   28242
Delta_2183060      CGTTCTATGAAGACTTTTTAGAGTATCATGACGTTCGTGTTGTTTTA------ATCTAAA   28237
Omicron_7869197    CGTTCTATGAAGACTTTTTAGAGTATCATGACGTTCGTGTTGTTTTAGATTTCATCTAAA   28228
                   ***********************************************      *******

w.t._745046        CGAACAAACTAAAATGTCTGATAATGGACCCCAAAATCAGCGAAATGCACCCCGCATTAC   28316
Alpha_737204       CGAACAAACT-AAATGTCTCTAAATGGACCCCAAAATCAGCGAAATGCACCCCGCATTAC   28301
Delta_2183060      CGAACAAACT-AAATGTCTGATAATGGACCCCAAAATCAGCGAAATGCACCCCGCATTAC   28296
Omicron_7869197    CGAACAAACTTAAATGTCTGATAATGGACCCCAAAATCAGCGAAATGCACTCCGCATTAC   28288
                   ********** ********   **************************** *********

w.t._745046        GTTTGGTGGACCCTCAGATTCAACTGGCAGTAACCAGAATGGAGAACGCAGTGGGGCGCG   28376
Alpha_737204       GTTTGGTGGACCCTCAGATTCAACTGGCAGTAACCAGAATGGAGAACGCAGTGGGGCGCG   28361
Delta_2183060      GTTTGGTGGACCCTCAGATTCAACTGGCAGTAACCAGAATGGAGAACGCAGTGGGGCGCG   28356
Omicron_7869197    GTTTGGTGGACCCTCAGATTCAACTGGCAGTAACCAGAATG---------GTGGGGCGCG   28339
                   *****************************************         **********

w.t._745046        ATCAAAACAACGTCGGCCCCAAGGTTTACCCAATAATACTGCGTCTTGGTTCACCGCTCT   28436
Alpha_737204       ATCAAAACAACGTCGGCCCCAAGGTTTACCCAATAATACTGCGTCTTGGTTCACCGCTCT   28421
Delta_2183060      ATCAAAACAACGTCGGCCCCAAGGTTTACCCAATAATACTGCGTCTTGGTTCACCGCTCT   28416
Omicron_7869197    ATCAAAACAACGTCGGCCCCAAGGTTTACCCAATAATACTGCGTCTTGGTTCACCGCTCT   28399
                   ************************************************************

w.t._745046        CACTCAACATGGCAAGGAAGACCTTAAATTCCCTCGAGGACAAGGCGTTCCAATTAACAC   28496
Alpha_737204       CACTCAACATGGCAAGGAAGACCTTAAATTCCCTCGAGGACAAGGCGTTCCAATTAACAC   28481
Delta_2183060      CACTCAACATGGCAAGGAAGGCCTTAAATTCCCTCGAGGACAAGGCGTTCCAATTAACAC   28476
Omicron_7869197    CACTCAACATGGCAAGGAAGACCTTAAATTCCCTCGAGGACAAGGCGTTCCAATTAACAC   28459
                   ******************** ***************************************

w.t._745046        CAATAGCAGTCCAGATGACCAAATTGGCTACTACCGAAGAGCTACCAGACGAATTCGTGG   28556
Alpha_737204       CAATAGCAGTCCAGATGACCAAATTGGCTACTACCGAAGAGCTACCAGACGAATTCGTGG   28541
Delta_2183060      CAATAGCAGTCCAGATGACCAAATTGGCTACTACCGAAGAGCTACCAGACGAATTCGTGG   28536
Omicron_7869197    CAATAGCAGTCCAGATGACCAAATTGGCTACTACCGAAGAGCTACCAGACGAATTCGTGG   28519
                   ************************************************************

w.t._745046        TGGTGACGGTAAAATGAAAGATCTCAGTCCAAGATGGTATTTCTACTACCTAGGAACTGG   28616
Alpha_737204       TGGTGACGGTAAAATGAAAGATCTCAGTCCAAGATGGTATTTCTACTACCTAGGAACTGG   28601
Delta_2183060      TGGTGACGGTAAAATGAAAGATCTCAGTCCAAGATGGTATTTCTACTACCTAGGAACTGG   28596
Omicron_7869197    TGGTGACGGTAAAATGAAAGATCTCAGTCCAAGATGGTATTTCTACTACCTAGGAACTGG   28579
                   ************************************************************

w.t._745046        GCCAGAAGCTGGACCTCCCTATGGTGCTAACAAAGACGGCATCATATGGGTTGCAACTGA   28676
Alpha_737204       GCCAGAAGCTGGACCTCCCTATGGTGCTAACAAAGACGGCATCATATGGGTTGCAACTGA   28661
Delta_2183060      GCCAGAAGCTGGACCTCCCTATGGTGCTAACAAAGACGGCATCATATGGGTTGCAACTGA   28656
Omicron_7869197    GCCAGAAGCTGGACCTCCCTATGGTGCTAACAAAGACGGCATCATATGGGTTGCAACTGA   28639
                   ************************************************************

w.t._745046        GGGAGCCTTGAATACACCAAAAGATCACATTGGCACCCGCAATCCTGCTAACAATGCTGC   28736
Alpha_737204       GGGAGCCTTGAATACACCAAAAGATCACATTGGCACCCGCAATCCTGCTAACAATGCTGC   28721
Delta_2183060      GGGAGCCTTGAATACACCAAAAGATCACATTGGCACCCGCAATCCTGCTAACAATGCTGC   28716
Omicron_7869197    GGGAGCCTTGAATACACCAAAAGATCACATTGGCACCCGCAATCCTGCTAACAATGCTGC   28699
                   ************************************************************

w.t._745046        AATCGTGCTACAACCTCCTCAAGGAACAACATTGCCAAAAGGCCTCTACGCAGAAGGGAG   28796
Alpha_737204       AATCGTGCTACAACCTCCTCAAGGAACAACATTGCCAAAAGGCCTCTACGCAGAAGGGAG   28781
Delta_2183060      AATCGTGCTACAACCTCCTCAAGGAACAACATTGCCAAAAGGCCTCTACGCAGAAGGGAG   28776
Omicron_7869197    AATCGTGCTACAACCTCCTCAAGGAACAACATTGCCAAAAGGCCTCTACGCAGAAGGGAG   28759
                   ************************************************************
```

```
w.t._745046        CAGAGGCGGCAGTCAAGCCTCCTCTCGTTCCTCATCACGTAGTCGCAACAGTTCAAGAAA   28856
Alpha_737204       CAGAGGCGGCAGTCAAGCCTCCTCTCGTTCCTCATCACGTAGTCGCAACAGTTCAAGAAA   28841
Delta_2183060      CAGAGGCGGCAGTCAAGCCTCCTCTCGTTCCTCATCACGTAGTCGCAACAGTTCAAGAAA   28836
Omicron_7869197    CAGAGGCGGCAGTCAAGCCTCCTCTCGTTCCTCATCACGTAGTCGCAACAGTTCAAGAAA   28819
                   ************************************************************

w.t._745046        TTCAACTCCAGGCAGCAGTAAACGAACCTCTCCTGCTAGAATGGCTGGCAATGGCGGTGA   28916
Alpha_737204       TTCAACTCCAGGCAGCAGTAAACGAACCTCTCCTGCTAGAATGGCTGGCAATGGCGGTGA   28901
Delta_2183060      TTCAACTCCAGGCAGCAGTATGGGAACCTCTCCTGCTAGAATGGCTGGCAATGGCGGTGA   28896
Omicron_7869197    TTCAACTCCAGGCAGCAGTAAACGAACCTCTCCTGCTAGAATGGCTGGCAATGGCGGTGA   28879
                   ********************   *************************************

w.t._745046        TGCTGCTCTTGCTTTGCTGCTGCTTGACAGATTGAACCAGCTTGAGAGCAAAATGTCTGG   28976
Alpha_737204       TGCTGCTCTTGCTTTGCTGCTGCTTGACAGATTGAACCAGCTTGAGAGCAAAATGTTTGG   28961
Delta_2183060      TGCTGCTCTTGCTTTGCTGCTGCTTGACAGATTGAACCAGCTTGAGAGCAAAATGTCTGG   28956
Omicron_7869197    TGCTGCTCTTGCTTTGCTGCTGCTTGACAGATTGAACCAGCTTGAGAGCAAAATGTCTGG   28939
                   ******************************************************** ***

w.t._745046        TAAAGGCCAACAACAACAAGGCCAAACTGTCACTAAGAAATCTGCTGCTGAGGCCTCTAA   29036
Alpha_737204       TAAAGGCCAACAACAACAAGGCCAAACTGTCACTAAGAAATCTGCTGCTGAGGCCTCTAA   29021
Delta_2183060      TAAAGGCCAACAACAACAAGGCCAAACTGTCACTAAGAAATCTGCTGCTGAGGCCTCTAA   29016
Omicron_7869197    TAAAGGCCAACAACAACAAGGCCAAACTGTCACTAAGAAATCTGCTGCTGAGGCCTCTAA   28999
                   ************************************************************

w.t._745046        GAAGCCTCGGCAAAAACGTACTGCCACTAAAGCATACAATGTAACACAAGCTTTCGGCAG   29096
Alpha_737204       GAAGCCTCGGCAAAAACGTACTGCCACTAAAGCATACAATGTAACACAAGCTTTCGGCAG   29081
Delta_2183060      GAAGCCTCGGCAAAAACGTACTGCCACTAAAGCATACAATGTAACACAAGCTTTCGGCAG   29076
Omicron_7869197    GAAGCCTCGGCAAAAACGTACTGCCACTAAAGCATACAATGTAACACAAGCTTTCGGCAG   29059
                   ************************************************************

w.t._745046        ACGTGGTCCAGAACAAACCCAAGGAAATTTTGGGGACCAGGAACTAATCAGACAAGGAAC   29156
Alpha_737204       ACGTGGTCCAGAACAAACCCAAGGAAATTTTGGGGACCAGGAACTAATCAGACAAGGAAC   29141
Delta_2183060      ACGTGGTCCAGAACAAACCCAAGGAAATTTTGGGGACCAGGAACTAATCAGACAAGGAAC   29136
Omicron_7869197    ACGTGGTCCAGAACAAACCCAAGGAAATTTTGGGGACCAGGAACTAATCAGACAAGGAAC   29119
                   ************************************************************

w.t._745046        TGATTACAAACATTGGCCTCAAATTGCACAATTTGCCCCCAGCGCCTCAGCGTTCCTCGG   29216
Alpha_737204       TGATTACAAACATTGGCCGCAAATTGCACAATTTGCCCCCAGCGCCTCAGCGTTCCTCGG   29201
Delta_2183060      TGATTACAAACATTGGCCGCAAATTGCACAATTTGCCCCCAGCGCCTCAGCGTTCCTCGG   29196
Omicron_7869197    TGATTACAAACATTGGCCGCAAATTGCACAATTTGCCCCCAGCGCCTCAGCGTTCCTCGG   29179
                   ****************** *****************************************

w.t._745046        AATGTCGCGCATTGGCATGGAAGTCACACCCTCGGGAACTTGGTTGACCTACACAGGTGC   29276
Alpha_737204       AATGTCGCGCATTGGCATGGAAGTCACACCCTCGGGAACGTGGTTGACCTACACAGGTGC   29261
Delta_2183060      AATGTCGCGCATTGGCATGGAAGTCACACCCTCGGGAACGTGGTTGACCTACACAGGTGC   29256
Omicron_7869197    AATGTCGCGCATTGGCATGGAAGTCACACCCTCGGGAACGTGGTTGACCTACACAGGTGC   29239
                   *************************************** ********************

w.t._745046        CATCAAATTGGATGACAAAGATCCAAATTTCAAAGATCAAGTCATTTTGCTGAATAAGCA   29336
Alpha_737204       CATCAAATTGGATGACAAAGATCCAAATTTCAAAGATCAAGTCATTTTGCTGAATAAGCA   29321
Delta_2183060      CATCAAATTGGATGACAAAGATCCAAATTTCAAAGATCAAGTCATTTTGCTGAATAAGCA   29316
Omicron_7869197    CATCAAATTGGATGACAAAGATCCAAATTTCAAAGATCAAGTCATTTTGCTGAATAAGCA   29299
                   ************************************************************

w.t._745046        TATTGACGCATACAAAACATTCCCACCAACAGAGCCTAAAAAGGACAAAAAGAAGAAGGC   29396
Alpha_737204       TATTGACGCATACAAAACATTCCCACCAACAGAGCCTAAAAAGGACAAAAAGAAGAAGGC   29381
Delta_2183060      TATTGACGCATACAAAACATTCCCACCAACAGAGCCTAAAAAGGACAAAAAGAAGAAGGC   29376
Omicron_7869197    TATTGACGCATACAAAACATTCCCACCAACAGAGCCTAAAAAGGACAAAAAGAAGAAGGC   29359
                   ************************************************************
```

```
w.t._745046        TGATGAAACTCAAGCCTTACCGCAGAGACAGAAGAAACAGCAAACTGTGACTCCTCTTCC   29456
Alpha_737204       TGATGAAACTCAAGCCTTACCGCAGAGACAGAAGAAACAGCAAACTGTGACTCCTCTTCC   29441
Delta_2183060      TTATGAAACTCAAGCCTTACCGCAGAAACAGAAGAAACAGCAAACTGTGACTCCTCTTCC   29436
Omicron_7869197    TGATGAAACTCAAGCCTTACCGCAGAGACAGAAGAAACAGCAAACTGTGACTCCTCTTCC   29419
                   * ************************ *********************************

w.t._745046        TGCTGCAGATTTGGATGATTTCTCCAAACAATTGCAACAATCCATGAGCAGTGCTGACTC   29516
Alpha_737204       TGCTGCAGATTTGGATGATTTCTCCAAACAATTGCAACAATCCATGAGCAGTGCTGACTC   29501
Delta_2183060      TGCTGCAGATTTGGATGATTTCTCCAAACAATTGCAACAATCCATGAGCAGTGCTGACTC   29496
Omicron_7869197    TGCTGCAGATTTGGATGATTTCTCCAAACAATTGCAACAATCCATGAGCAGTGCTGACTC   29479
                   ************************************************************

w.t._745046        AACTCAGGCCTAAACTCATGCAGACCACACAAGGCAGATGGGCTATATAAACGTTTTCGC   29576
Alpha_737204       AACTCAGGCCTAAACTCATGCAGACCACACAAGGCAGATGGGCTATATAAACGTTTTCGC   29561
Delta_2183060      AACTCAGGCCTAAACTCATGCAGACCACACAAGGCAGATGGGCTATATAAACGTTTTCGC   29556
Omicron_7869197    AACTCAGGCCTAAACTCATGCAGACCACACAAGGCAGATGGGCTATATAAACGTTTTCGC   29539
                   ************************************************************

w.t._745046        TTTTCCGTTTACGATATATAGTCTACTCTTGTGCAGAATGAATTCTCGTAACTACATAGC   29636
Alpha_737204       TTTTCCGTTTACGATATATAGTCTACTCTTGTGCAGAATGAATTCTCGTAACTACATAGC   29621
Delta_2183060      TTTTCCGTTTACGATATATAGTCTACTCTTGTGCAGAATGAATTCTCGTAACTACATAGC   29616
Omicron_7869197    TTTTCCGTTTACGATATATAGTCTACTCTTGTGCAGAATGAATTCTCGTAACTACATAGC   29599
                   ************************************************************

w.t._745046        ACAAGTAGATGTAGTTAACTTTAATCTCACATAGCAATCTTTAATCAGTGTGTAACATTA   29696
Alpha_737204       ACAAGTAGATGTAGTTAACTTTAATCTCACATAGCAATCTTTAATCAGTGTGTAACATTA   29681
Delta_2183060      ACAAGTAGATGTAGTTAACTTTAATCTCACATAGCAATCTTTAATCAGTGTGTAACATTA   29676
Omicron_7869197    ACAAGTAGATGTAGTTAACTTTAATCTCACATAGCAATCTTTAATCAGTGTGTAACATTA   29659
                   ************************************************************

w.t._745046        GGGAGGACTTGAAAGAGCCACCACATTTTCACCGAGGCCACGCGGAGTACGATCGAGTGT   29756
Alpha_737204       GGGAGGACTTGAAAGAGCCACCACATTTTCACCGAGGCCACGCGGAGTACGATCGAGTGT   29741
Delta_2183060      GGGAGGACTTGAAAGAGCCACCACATTTTCACCGAGGCCACTCGGAGTACGATCGAGTGT   29736
Omicron_7869197    GGGAGGACTTGAAAGAGCCACCACATTTTCACCGAGGCCACGCGGAGTACGATCGAGTGT   29719
                   ***************************************** ******************

w.t._745046        ACAGTGAACAATGCTAGGGAGAGCTGCCTATATGGAAGAGCCCTAATGTGTAAAATTAAT   29816
Alpha_737204       ACAGTGAACAATGCTAGGGAGAGCTGCCTATATGGAAGAGCCCTAATGTGTAAAATTAAT   29801
Delta_2183060      ACAGTGAACAATGCTAGGGAGAGCTGCCTATATGGAAGAGCCCTAATGTGTAAAATTAAT   29796
Omicron_7869197    ACAGTGAACAATGCTAGGGAGAGCTGCCTATATGGAAGAGCCCTAATGTGTAAAATTAAT   29779
                   ************************************************************

w.t._745046        TTTAGTAGTGCTATCCCCATGTGATTTTAATAGCCTCTT---------------------   29855
Alpha_737204       TTTAGTAGTGCTATCCCCATGTGATTTTAATAGC--------------------------   29835
Delta_2183060      TTTAGTAGTGCTATCCCCATGTGATTTTAATAGCCTCTTAGGAGAATGACAAAAAAAAAA   29856
Omicron_7869197    TTTAGTAGTGCTATCCCCATGTGATTTTAATAGCTTNNNNNNNNNNNNNACAAAAAAAAAA   29839
                   **********************************

w.t._745046        -----------------------  29855
Alpha_737204       -----------------------  29835
Delta_2183060      AAAAAAAAAAAAAAAAAAAAAAA  29879
Omicron_7869197    AAAAAAAAAAAAAAAAAAAAAAA  29862
```
